# Differential growth and flowering capacity of tulip bulbs and the potential involvement of *PHOSPHATIDYLETHANOLAMINE-BINDING PROTEINS (PEBPs)*

**DOI:** 10.1186/s13062-025-00625-y

**Published:** 2025-03-10

**Authors:** Francesca Bellinazzo, Irene Manders, Bas Heidemann, Manuel Aguirre Bolanos, Evelien Stouten, Jacqueline Busscher, Dolores Abarca, Froukje van der Wal, Marcelo Carnier Dornelas, Gerco C. Angenent, Marcel Proveniers, Harm Nijveen, Richard G. H. Immink

**Affiliations:** 1 Laboratory of Molecular Biology, Cluster Plant Developmental Biology, Wageningen University and Research, 6708 PB Wageningen, The Netherlands; 2 Bioscience, Wageningen Plant Research, Wageningen University and Research, 6708 PB Wageningen, The Netherlands; 3 Laboratory of Cell and Developmental Biology, Cluster Plant Developmental Biology, Wageningen University and Research, 6708 PB Wageningen, The Netherlands; 4 Translational Plant Biology, Department of Biology, Science4Life, Utrecht University, Padualaan 8, 3584 CH Utrecht, The Netherlands; 5 Plant Stress Resilience, Department of Biology, Science4Life, Utrecht University, Padualaan 8, 3584 CH Utrecht, The Netherlands; 6 Department of Life Sciences, University of Alcalá, Alcalá de Henares, 28805 Madrid, Spain; 7 Departamento de Biologia Vegetal, Instituto de Biologia, Universidade Estadual de Campinas, Campinas, SP CEP 13083-862 Brazil; 8 Bioinformatics Group, Wageningen University and Research, PO Box 633, 6700 AP Wageningen, The Netherlands

**Keywords:** Tulip, Flowering, Reproduction, Bulbing, Dormancy, *PEBP* genes, Transcriptional regulation

## Abstract

**Background:**

*Tulipa gesneriana* reproduces vegetatively by the development of bulb clusters from axillary meristems in the scales of a mother bulb. While part of the daughter bulbs in a cluster develop into large, flowering bulbs, others stay small and vegetative under the same environmental conditions. This study aims to investigate how these different developmental fates are orchestrated.

**Results:**

RNA-seq analysis revealed that the overall transcriptomic landscape of the two types of daughter bulbs does not differ substantially, but follows a similar trajectory over time. Nonetheless, the expression levels of genes related to proliferation already differ at early development stages. Surprisingly, at a later stage, transcriptomic changes related to flower induction are detectable in flowering as well as non-flowering bulbs, with some quantitative differences. However, genes linked with floral organ development are differentially expressed, as well as negative regulators of flowering and more basal metabolic processes. In search for the molecular determinants of daughter bulb size and developmental fate, we investigated members of the *PHOSPHATIDYLETHANOLAMINE-BINDING PROTEIN (PEBP)* gene family as candidates. Tulip *FLOWERING LOCUS T1 (TgFT1), TgFT2,* and *TgFT3* are expressed in leaves and leaf-like organs of the mother plant, and their encoded proteins interact with the TCP transcription factor TEOSINTE BRANCHED1 (TgTB1). Therefore, we suggest that these three genes act as ‘bulbigens’, meaning regulators of axillary meristem outgrowth and hence, daughter bulb size. Furthermore, we found that *TgFT2* and *TgFT4* could constitute the main florigens in tulips, because of their expression pattern and the binding of their encoding proteins to the bZIP transcription factor FD (TgFD). Moreover, Arabidopsis lines ectopically expressing *TgFT2* or *TgFT4* flower significantly earlier than the wild type.

**Conclusions:**

Differences in the developmental fate of tulip daughter bulbs are established early during development and are linked with differences in cell division and metabolism. The activity of members of the PEBP family, known for their role in flowering and storage organ formation in geophytes, appeared to be associated with the transcriptional switches observed during daughter bulb development. This points towards a functional role of these proteins in governing developmental trajectories underlying the mode of reproduction.

**Supplementary Information:**

The online version contains supplementary material available at 10.1186/s13062-025-00625-y.

## Background

**Fig. 1:**
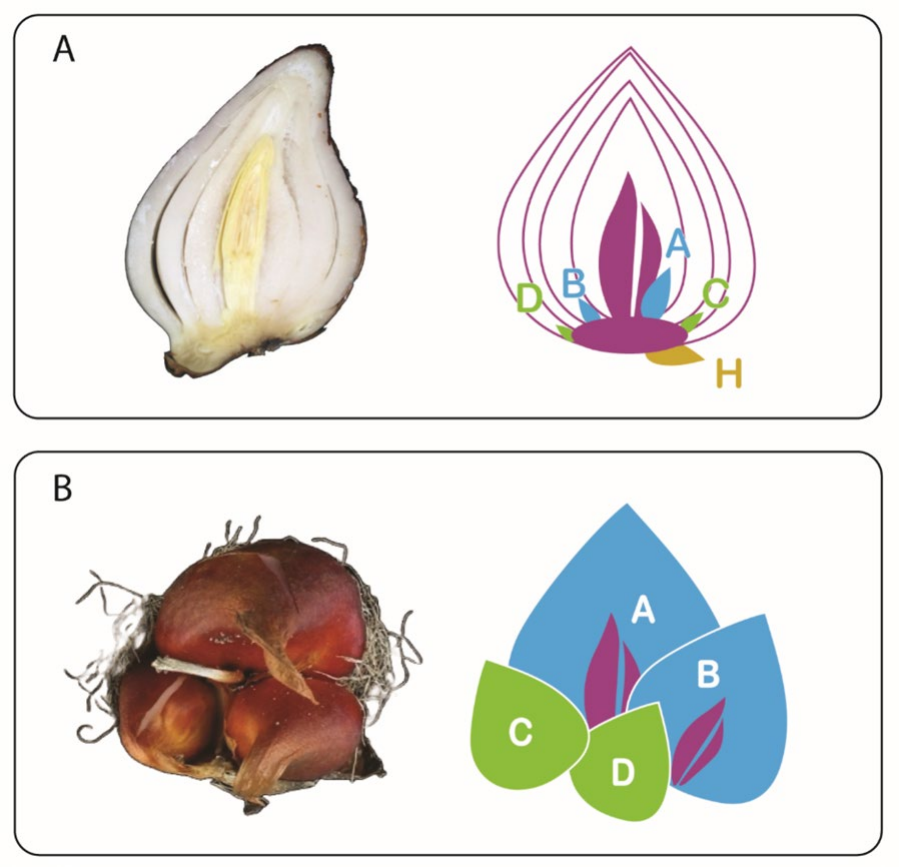
Schematic representation of daughter bulb development in tulip. (**A**) Cross section of a mother bulb at the moment of planting in the field just before winter (left). The yellow structure in the centre is the flower. On the right a schematic representation is depicted showing the daughter bud positions with alphabetic letters and the basal plate with central flower bud in purple. (**B**) Top view of a daughter bulb cluster at the end of the growth season, in summer (left) and its schematic depiction (right). Daughter bulb D is small and not visible in the picture. Each daughter bud develops into a daughter bulb. The bigger bulbs, in light blue, contain a flower bud (purple) that will bloom in the next growing season, in spring. In this study, they are referred to as ‘flowering’ daughter bulbs (F). The smaller bulbs, in green, remain vegetative and produce one flat leaf. Here, they are referred to as ‘non-flowering’ daughter bulbs (NF). Both F and NF bulbs can produce a new generation of daughter bulbs following the same principle

The notorious ornamental crop tulip (*Tulipa gesneriana*) is a bulbous plant species, which propagates sexually through flowers and seeds, and asexually through the formation of daughter bulbs. To date, the molecular regulators of tulip reproduction are largely unknown. Adult tulip bulbs are made of concentric whorls of fleshy leaves, called scales, which accumulate nutrients to sustain the future growth of a stalk, leaves, and a flower. Furthermore, axillary buds are present at the base of each scale on the adaxial side, and are connected to the basal plate, a compressed stem-like structure at the bottom of the bulb. An adult bulb usually contains four or five scales and an equal number of axillary buds, which are named alphabetically, starting from the internal whorl ‘A’ and ending with the external one, which can be e.g., ‘D’ or ‘E’, depending on the total number of scales. One additional bud, called ‘H’, is formed outside the outermost scale, attached to the basal plate. While the mother bulb decays at the end of the growth cycle, these axillary buds grow out into a cluster of mature daughter bulbs of different sizes, held together by the dried organs of the mother bulb (Fig. 1) [9].

Tulip flowers are initiated inside mature daughter bulbs, soon after they reach a critical size [29], De Hertogh, Aung and Benschop, [13]. The final size of daughter bulbs is associated with their relative position inside the mother bulb and correlates with the expression level of *TgTB1*, the potential tulip ortholog of the TCP transcription factor *BRANCHED 1* (*BRC1*)/ *TEOSINTE BRANCHED 1 (TB1). BRC1/TB1* is one of the main regulators of axillary bud outgrowth in plants [2, 27, 40]. In tulip, *TgTB1* expression is low in A-buds and increases in peripheral buds, following a gradient culminating at D-buds and decreasing again in E-buds until being almost undetectable in H-buds [27]. At the end of the growing season, A- and B- daughter bulbs normally form a flower bud under inductive high temperatures. These flower buds will reach anthesis only in the following spring. Contrarily, C-, D- and E- daughter bulbs, which are smaller in size, usually remain in a vegetative stage, even when receiving flower-inducive environmental signals [29], De Hertogh, Aung and Benschop, [13].

Flower induction in plants is regulated by multiple molecular pathways, each one responding to a different environmental or internal signal. These pathways converge onto a small number of central regulators which mediate a simple on/off flowering response when a particular threshold is reached. This infers that flower induction is a highly dynamic and quantitative trait. It is known from studies in *Arabidopsis thaliana* that among the flowering integrators, *SUPPRESSOR OF OVEREXPRESSION OF CONSTANS 1 (SOC1)* and *FLOWERING LOCUS T (FT)* work as molecular hubs, receiving signals from the multiple upstream pathways and inducing downstream expression of flower-meristem identity genes such as *APETALA 1 (AP1)* and *LEAFY (LFY)* [5]. *FT*, also known as florigen, is a member of the *PHOSPHATIDYLETHANOLAMINE-BINDING PROTEINS (PEBP)* gene family and is generally expressed and translated in leaf vasculature in response to favourable day-length conditions. After being loaded into the phloem, FT is systemically transported and enters the shoot apical meristem (SAM), where it binds to the bZIP transcription factor FD to activate the expression of downstream genes (Turck, Fornara and Coupland, 2008). The function of FT as florigen, defined as a transmissible inducive flowering signal, is highly conserved among angiosperms. Nevertheless, expression of *FT* genes with florigenic activity has also been found in the SAM itself under flowering-stimulating conditions in e.g., rice [11]. Another member of the PEBP family, TERMINAL FLOWER 1 (TFL1), is produced at the SAM and functions as a flower repressor by competing with FT for binding to FD and blocking the flowering cascade. The balance between FT and TFL1 regulates the timing of floral induction and the degree of meristem determinacy, ultimately affecting inflorescence development [10, 14, 19, 43].

The relationship between outgrowth and flower induction in axillary buds has been described in the model species Arabidopsis, where BRC1 interacts with FT to suppress flower induction in axillary shoots [31]. Moreover, FT has been shown to have a direct effect on axillary shoot outgrowth [15, 17]. Finally, the concerted action of *FT* and *BRC1* regulates bud outgrowth and flowering in the axillary branches of hybrid aspen in response to day-length signals [25]. Therefore, it seems that BRC1 plays a double role as a repressor of outgrowth and flowering, working at least partially in concert with FT to fulfil such roles.

Additionally, *FT* genes are known for regulating storage organ formation in geophytes, the plants that produce storage organs such as tubers, rhizomes, corms, and bulbs [18]. For example, potato StSP6A interacts with an FD-like protein to promote tuber formation under Short Day (SD) conditions, while StSP5G is a repressor of tuberization under Long Day (LD) conditions [1, 34, 37]. Onion *AcFT1* is a bulb inducer, or ‘bulbigen’, while *AcFT4* is a bulb repressor, or ‘anti-bulbigen’ [20]. In analogy to Arabidopsis and hybrid aspen [25], potato PEBPs and BRC1*-*like proteins show interaction,this is the case for a specialized BRC1-like protein, BRC1b, which is produced in leaf axils to repress the formation of aerial tubers in response to StSP6A [28].

For tulips, an increase in temperature is a very strong flowering trigger (De Hertogh, Aung and Benschop, [13, 22], and it is associated with a decrease in the expression of *TgTFL1* followed by an increase in the expression of *TgFT-like* [22] (renamed *TgFT4* in this study) in meristem-rich samples of flowering daughter bulbs. Meristematic origin of putative florigenic signals has been previously reported in other geophytes, such as *Narcissus tazetta* [32] and *Lilium* [41], both in response to a temperature cue. *TgFT4*, however, is not the only candidate gene for the role of florigen in tulip: in fact, multiple *FT* genes are present of which *TgFT1*, *TgFT2,* and *TgFT3* have been partially characterized previously. Among these, *TgFT2* induced early flowering when overexpressed in Arabidopsis. Additionally, *TgFT2* is expressed in tulip leaves, in analogy to florigen expression in Arabidopsis and rice. Therefore, *TgFT2* is considered another good florigen candidate in tulip [23].

This study aims to identify the molecular determinants that underlie the differential development of tulip daughter bulbs, by comparing large, flowering daughter bulbs (F) and small, non-flowering daughter bulbs (NF). Furthermore, we hypothesize that genes of the *PEBP* family are potentially good candidates as regulators of bulb size and flowering capacity of tulip daughter bulbs and therefore members of this family were investigated. Comparing the transcriptomic landscape of F versus NF daughter bulbs revealed that genes associated with floral induction are similarly expressed; while genes linked to floral organ development are significantly upregulated in F daughter bulbs. Further characterization of tulip *PEBP* genes pointed to *TgFT1* and *TgFT3* as putative regulators of daughter bulb outgrowth, *TgFT4* as a positive regulator of flower induction, and *TgFT2* could potentially be involved in both processes. In conclusion, tulip *PEBPs* seem to act in a partially specialized fashion to orchestrate the outgrowth of daughter bulbs and their capacity to switch to the flowering state.

## Methods

### Plant material and growth conditions

*Tulipa gesneriana* bulbs, cultivar Dynasty, size 9–10 (Gulik tulips, De Goorn, NL) were cultivated in the experimental fields of Wageningen University (Wageningen, NL). On 6th December 2018, all bulbs were planted in the same plot, in rows of 10, at approximately 30 cm distance from one another. On 26th April 2019, flowers were cut, as it is common practice in tulip cultivation for bulb production. On the 9th of September, tulip bulbs were lifted from the ground.

Tulip bulbs from the same cultivar were cold-stored (4–10 °C) in the dark for Western blot experiments.

*Arabidopsis thaliana* Col-0 (NASC) and the generated transgenic plants using this background were cultivated in growth chambers at 70% relative humidity at 20 °C in Long Day conditions (16/8 h light/dark). LED light (150 μmol m^−2^ s^−1^). Cultivation in hydroponics on rockwool blocks (Grodan) and watered with a solution of Hyponex fertilizer (~ 1 g/L) twice a week.

### SDS-PAGE and Western blotting

Frozen, finely cryo-ground bulb samples were suspended in 200 µL Laemmli buffer with Pierce protease and phosphatase inhibitor (Thermo Scientific), boiled for 15 min, and spun for 5 min at 13,000 rpm. Supernatant protein concentrations were quantified using the Pierce™ BCA Protein Assay Kit (Thermo Scientific). Ten micrograms of protein in LDS sample buffer with Bolt/NuPAGE reducing agent (ThermoFisher) were heated for 10 min at 70ºC, separated in SDS-PAGE gels, and transferred to polyvinylidene difluoride membranes (PVDF, Invitrogen) by electroblotting. Membranes were blocked with Blocker™ BSA (ThermoFisher) plus 0,05% Tween20 and probed with rabbit anti-RPS6A (Agrisera AS19 4292), anti-RPS6A-P240 (Agrisera AS19 4302) affinity purified or ant-tubulin alpha chain (Agrisera AS10 680) polyclonal antisera diluted 1:1000. Anti-rabbit IgG-HRP (horseradish peroxidase, Agrisera) 1:100,000 was used as secondary antibody. Immunodetection was performed using SuperSignal™ West Pico PLUS Chemiluminescent Substrate (ThermoFisher).

### Sampling for RNA isolation

Tulip plants were grown in the experimental fields of Wageningen University (NL). Different tissue types (meristem-enriched, leaves, scales) were sampled every two weeks until 29th June 2019 (28 weeks after planting (WAP)), and subsequently every week until the end of the experiment. For each time point, two consecutive rows were harvested. Plants were divided into 4 biological replicates containing 5 individuals each. Leaf samples were collected starting from 27th February 2019 (12 WAP), when the leaves started to unfold above ground, until 22nd May 2019 (24 WAP) when the leaves were starting to senesce. A middle horizontal section of every leaf was harvested and pooled together. Scale material was harvested from the start of the experiment until 22nd May 2019 (24 WAP), whereafter it was not possible to sample because of senescence. From the scales, only a vertical section surrounding the axillary bud was collected. Last, as meristem-enriched samples, full buds were harvested until 10th April 2019 (18 WAP). Subsequently, because of the bigger size of the forming bulbs, only the region around the SAM was harvested. All material was flash-frozen in liquid nitrogen and stored at − 80 °C.

Arabidopsis leaves (2 leaves per plant) were harvested from young vegetative seedlings at the 6-leave stage. Leaves from 4 individual plants were pooled together in one biological replicate, for a total of 3 replicates. The collected samples were immediately frozen in liquid nitrogen and stored at − 80 °C.

### RNA isolation and sequencing

Tulip material was finely cryo-ground using a mortar and pestle, or an electric mill for bigger amounts of material (IKA-A11 basic Analytical mill). Total RNA from scales and meristem-rich samples was isolated using the Tripure protocol (Roche) with an additional 2% w/v Polyvinylpyrrolidone (PVP) and 2% v/v β-mercaptoethanol (β-ME) in the extraction buffer. DNA was removed using the Turbo DNase kit (Thermofisher). The obtained RNA was additionally purified using the RNeasy PowerClean Pro CleanUp Kit (Qiagen). Total RNA from tulip leaves was extracted using the InviTrap® Spin Plant RNA Mini Kit (Invitek) and subsequently treated as indicated above. Library preparation and RNA sequencing (150 nt paired ends, Illumina NovaSeq 6000) were outsourced to Novogene. The raw sequencing data are available from NCBI’s Sequence Read Archive (BioProject PRJNA777886).

Arabidopsis samples were ground using a tissue lyser (3 M ESPE, CapMix). Total RNA isolation was performed with the InviTrap® Spin Plant RNA Mini Kit (Invitek) and DNase treatment was conducted with the Turbo DNA-free kit (Invitrogen). From the obtained RNA, cDNA was synthesized using the iScript Select kit (Bio-rad, The Netherlands) using a custom oligo-dT primer (5’-TTTTTTTTTTTTTTTTTTTTVN-3’).

### RNA-seq data analysis

The sequenced reads were pseudo-aligned to the available tulip transcriptome [26] using kallisto (version 0.46.2) with 50 bootstrap samples. Transcript counts were aggregated to gene level using tximport for differential expression analysis with DESeq2. Differential gene expression was performed using the Wald test with a significance threshold of 0.05.

### GO analysis

The enrichGO function of the R package clusterProfiler was used for GO enrichment analysis of the differentially expressed genes. Each tulip gene was mapped to the best-matching Arabidopsis gene using Blastp, with an e-value cut-off of 10^–5^ [3]. For the Arabidopsis GO annotation the org.At.tair.db package was used. As background, all Arabidopsis gene IDs were included with a tulip match in the results. The Benjamini–Hochberg method was selected for multiple testing corrections, with a q-value cut-off of 0.05.

### Identification of tulip transcripts and cloning

The novel tulip *PEBP* transcripts (*TgFT5 andTgFT6*) were identified by Blastx search on different transcriptomic data (Dummen Orange, unpublished; current RNA-seq data SRA submission number SUB10618278 and SUB11963046 [26].

To identify the putative TgFD, sequences of Tulip bZIPs were obtained from the same datasets by first predicting open reading frames using TransDecoder [https://github.com/TransDecoder/TransDecoder] with default settings and searching for the HMM profiles of bZIP_1 (PF00170), bZIP_2 (PF07716), and bZIP_Maf (PF03131) [https://pfam.xfam.org/] with hmmsearch [hmmer.org] using the following parameters – domtblout -E 0.01 –domeE 0.01. Subsequently, the protein sequences corresponding to the bZIP domain were used to construct a phylogenetic analysis together with sequences of Arabidopsis, potato, and Rice. These sequences were collected from TAIR, Rice Genome Annotation Project, and Spud DB [30, 42]. The collected sequences were aligned using the MUSCLE algorithm in MEGA11 and subsequently used for constructing a maximum likelihood tree using IQTREE 2 (default parameters) and visualized and annotated using FigTree (v1.4.4) (Supplementary Fig. 5).

Obtainment of full-length *TgTB1* sequence, from the partial, 406 bp *TgTB1* fragment [27]) was achieved by BLAST + . The identified fragments were aligned with Clustal Omega and based on that alignment reconstructed to one transcript. Open reading frames were detected using ORFfinder [https://www.ncbi.nlm.nih.gov/orffinder/]. To determine whether the reconstructed transcript was full length, an alignment was made using the *Lilium Longiflorum TB1* using Clustal Omega (Supplementary Fig. 2).

*TgTB1* was amplified using a cDNA expression library from a pool of different Tulip tissues as a template, with Q5® High-Fidelity DNA Polymerase (for the primers used, see Supplementary Table 1). Fragments showing the expected size were purified using the Macherey–Nagel NucleoSpin Gel and PCR Clean-up, according to manufactures specifications. The purified product was A-tailed and ligated in the pGEM®-T Vector and checked by Sanger sequencing. Once obtained, the pGEM®-T-*TgTB1* plasmid was used as template to construct an entry clone. AttB sites were added to the *TgTB1* fragment using PCR with Q5® High-Fidelity DNA Polymerase and the resulting product was cloned into pDONR201 through a BP reaction (Gateway).

### PEBP Phylogenetic analysis

PEBP protein sequences from tulip (*Tulipa gesneriana*), Arabidopsis (*Arabidopsis thaliana*), potato (*Solanum tuberosum)*, grape *(Vitis vinifera*), onion *(Allium cepa)*, lily *(Lilium sativum*) and rice (*Oryza sativa*) were retrieved by Blastp using the Arabidopsis FT (AT1G65480), TFL1 (AT5G03840) and MFT (AT1G18100) as a query. Sequence alignments were performed with the software MEGA-X; the Muscle algorithm was applied with default settings. Phylogenetic analyses were conducted with the software MEGA-X using the Maximum Likelihood (ML) method. Statistical analysis has been implemented with the bootstraps’ method with 500 repetitions.

### qPCR

RT-qPCR was performed using the iQ SYBR Green Supermix (Bio-rad) with a CFX6 instrument. The amplification protocol was as follows: 3 min 95 °C followed by 45 cycles of 10 s 95 °C, 30 s 60 °C. qPCR primers can be found in Supplementary Table 1. Expression was quantified against the reference gene *TgACT* using the 2^− Δct^ method [24].

### Ectopic overexpression lines in Arabidopsis thaliana

The fragments of interest, cloned into pDONR201 as described above, were transferred to the gateway expression vector pGD625 through an LR reaction (Gateway) and transformed into the *Agrobacterium tumefaciens* C58 strain via electroporation. Arabidopsis Col-0 plants were transformed with the transformed C58 strains containing the overexpression constructs using the floral dip method, with modifications [6]. Primary transformants were selected on 0.5 MS medium with Kanamycin (25 mg/L) and subsequently selfed until the S2 generation.

### Yeast two-hybrid

Yeast two-hybrid (Y2H) was executed as described previously [8]. No bait showed autoactivation and therefore, protein–protein interaction was tested on SD-LWH + 1 mM 3-AT.

### Phenotyping

Arabidopsis overexpression lines were scored for flowering time and the percentage of active axillary buds. Plants were randomized into 8 trays, which constitute the biological replicates. Each biological replicate contained 4 or sometimes 3 plants of each line. In the case of *35S::TgFT4*, the tray information was lost, so 8 groups were randomly generated during data analysis. Flowering time was scored by counting the number of rosette leaves at visual bolting. The percentage of active axillary buds was measured by counting the number of shoots originating from the axils of rosette leaves, normalized by the number of rosette leaves (as assessed to score for flowering time). A pairwise T-test assuming equal variance was used to assess statistical significance between Col-0 and each overexpression line.

## Results

### The transcriptome of F and NF daughter bulbs

Although exposed to the same environmental conditions, tulip axillary daughter bulbs have different growth and flowering capacities according to their spatial origin from inside the adult mother bulb, indicating the existence of an intrinsic regulatory mechanism. The biggest, flowering bulb (F) is produced from the first axil near the apex of the mother bulb, and the smallest, non-flowering bulb (NF) grows from the axillary bud in the fourth whorl towards the periphery of the mother bulb (Rees, [36],Natalia M. [27] (Fig. 1).

**Fig. 2:**
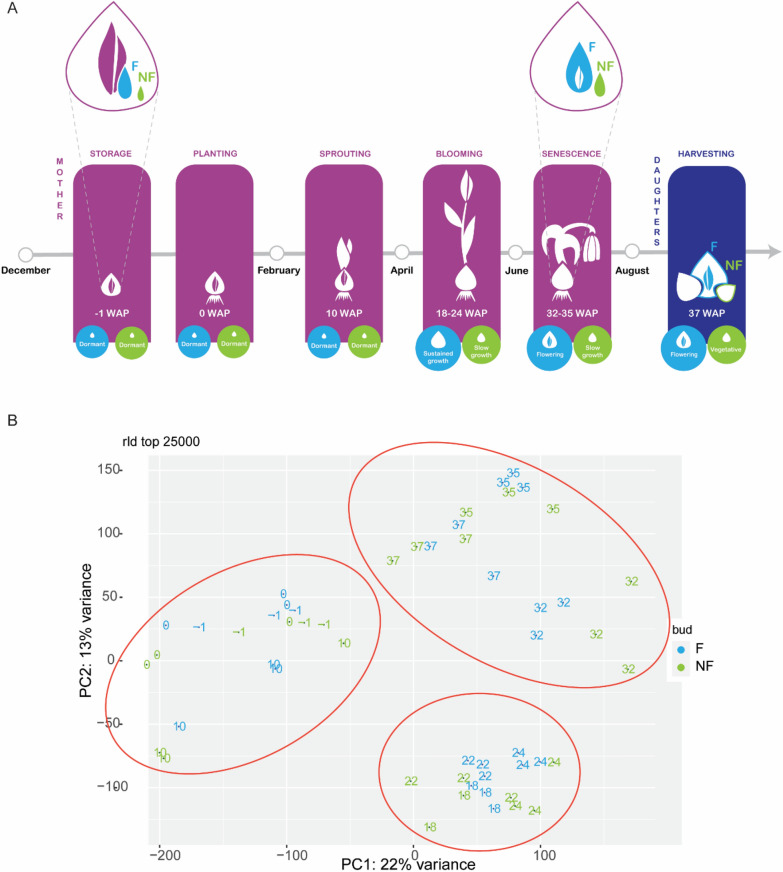
Transcriptomic comparison of meristem-enriched samples from F and NF axillary meristems and tulip daughter bulbs during the growth season. (**A**) Graphic representation of the simultaneous development of mother plant, F, and NF daughter bulbs during a growth season. Sampling times are indicated as WAP = Weeks After Planting. Mother bulbs were planted in early December (0 WAP). During winter, a shoot formed by three or four leaves slowly unfolds (10 WAP) and, in spring, a flower stalk elongates until the blooming stage (18 WAP). Subsequently, the mother plant starts to senesce, transferring its nutrients to the growing daughter bulbs (32 WAP). After a period of dormancy lasting for the whole autumn and winter, future F buds (in blue) undergo sustained growth until maturity, forming large bulbs. In late spring, flower induction occurs (24 WAP), and a flower bud is formed at the SAM. NF buds (in green) resume their growth more slowly. This results in a smaller bulb which, at maturity, does not form a flower, but one flat leaf instead. (**B**) Principal Component Analysis (PCA) visualizing the transcriptomic changes of F and NF daughter bulbs over time. Numbers indicate the sampling points in WAP

**Fig. 3:**
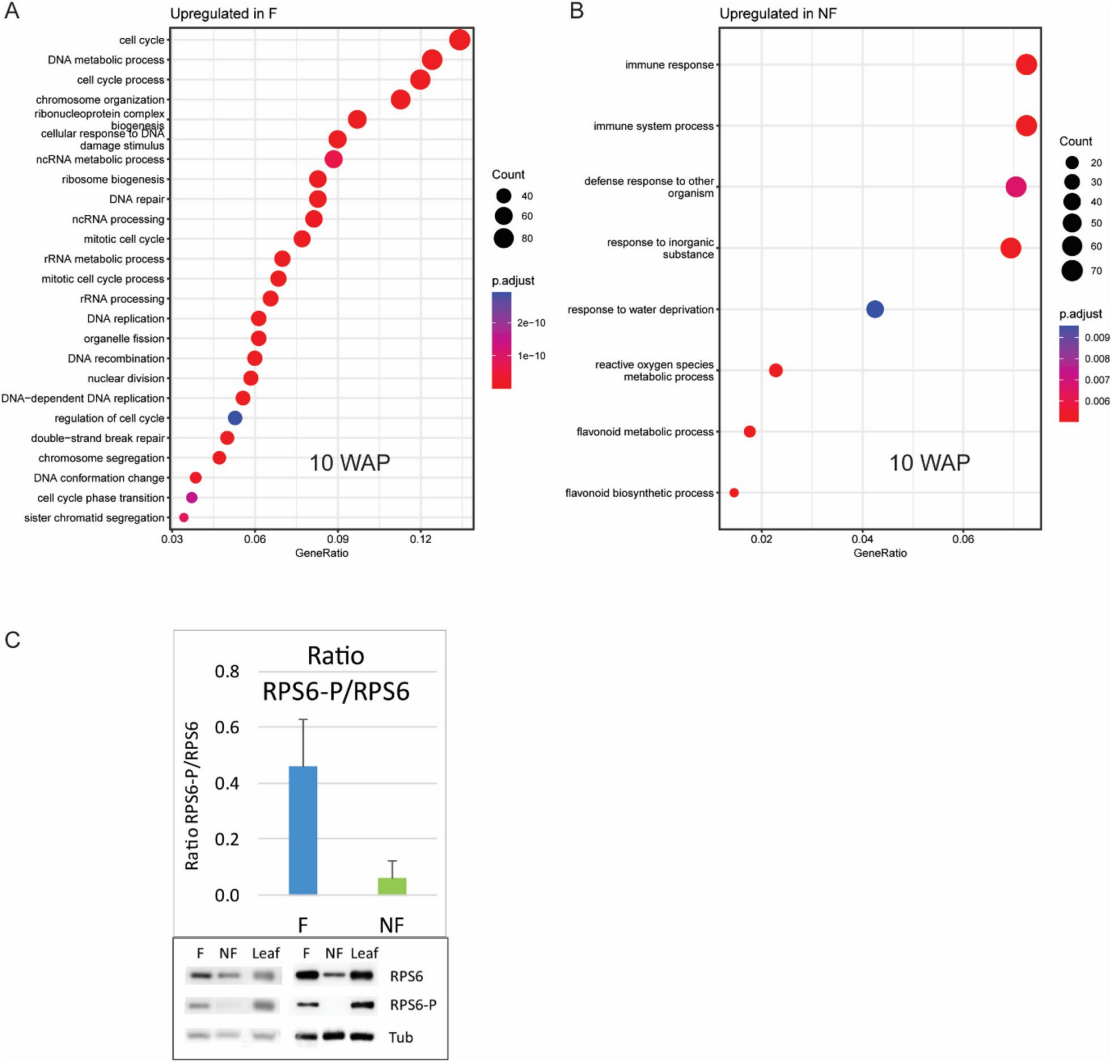
Differentially Expressed Genes (DEGs) and RPS6 phosphorylation in tulip F and NF axillary buds and daughter bulbs. (**A**) Top 25 GO enrichment terms of upregulated genes in F daughter bulbs at 10 weeks after planting (WAP). (**B**) GO enrichment analysis of upregulated genes in NF daughter bulbs at 10 WAP. (**A-B**) At the x-axis, GeneRatio indicates the fraction of genes associated with a specific term, over the total number of DEGs. (**C**) Relative levels of RIBOSOMAL PROTEIN S6 (RPS6) phosphorylation in F and NF cold-stored bulbs. Ratio’s calculated based on the quantification of Western blots. Bottom: two representative Western blots. Tubulin (Tub) was used as a loading control and for normalization

To identify the molecular players associated with the divergent developmental fate of F and NF daughter bulbs, their transcriptome was compared during an entire growth season (Fig. 2A). An overview of the transcriptomic changes in meristem-rich samples from F and NF axillary buds developing into daughter bulbs is displayed in Fig. 2B. Three main groups with more equal overall expression signatures are distinguishable: samples collected between -1 WAP and 10 WAP are grouped, and so are the samples collected between 18 and 24 WAP, and the ones collected between 32 and 37 WAP. Surprisingly, despite the clear differences in outgrowth and development, samples from F and NF axillary buds and bulbs are included in the same clusters, indicating similar overall transcriptional changes over time (Fig. 2B, Supplementary Fig. 1A-B). Nevertheless, a more detailed comparison between the transcriptomes of F and NF axillary buds and bulbs showed that already at 10 WAP multiple genes were differentially expressed (Supplementary Fig. 1C). GO enrichment analysis revealed that numerous genes associated with the terms ‘cell cycle’, ‘chromosome organization’, or ‘nuclear division’ are upregulated in developing F daughter bulbs (Fig. 3A). Instead, genes related to stress responses such as ‘response to water deprivation’, ‘reactive oxygen species metabolic processes’, and ‘flavonoid biosynthetic processes’ are upregulated in NF daughter bulbs (Fig. 3B), representing genes previously associated with negative regulation of cell proliferation [35]. All these enriched GO-terms point to a strong difference in cell proliferation and growth which is in line with the differential mass gain between the two types of daughter bulbs that we observed and that was previously described [27]. In accordance with this previous study, expression of the putative axillary bud growth inhibitor *TgTB1* appears to be higher in NF buds at early stages (Supplementary Fig. 2A). Altogether, these observations prompted us to follow the activity of F and NF daughter buds during cold storage in an independent experiment by measuring the level of RIBOSOMAL PROTEIN S6 (RPS6) phosphorylation. This analysis showed that upon cold treatment, the levels of RPS6 phosphorylation are higher in F than in NF daughter buds (Fig. 3C), indicating that F buds are much more responsive to dormancy-breaking and meristem activating cold conditions than NF bulbs.

**Fig. 4:**
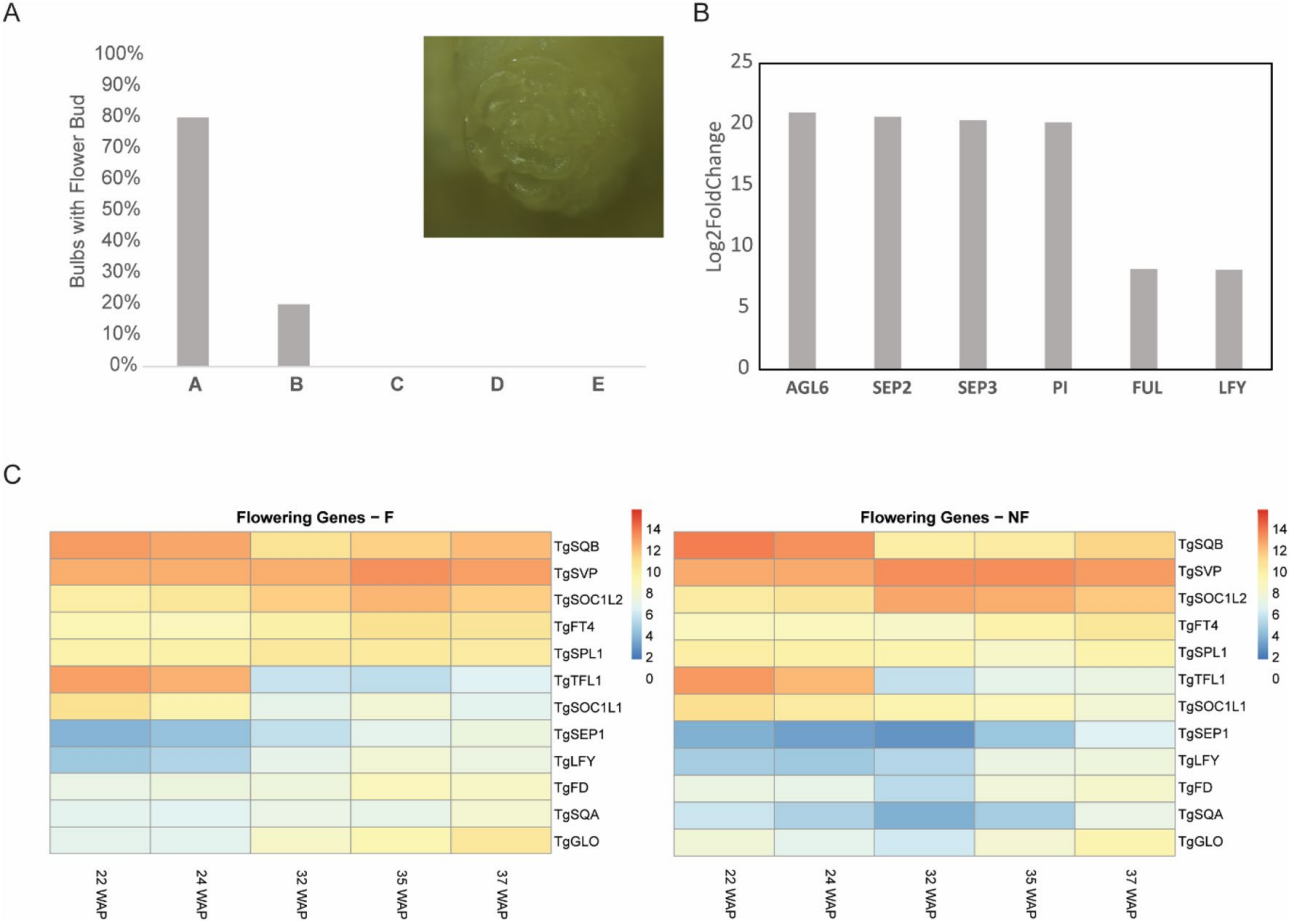
Differentially Expressed Genes (DEGs) in tulip F and NF axillary buds and daughter bulbs before the flowering stage. (**A**) Quantification of flower bud presence in daughter bulbs at 32 WAP. Each letter represents the corresponding daughter bulb category. Inset: Stereomicroscopic image of a flower bud at the apex of an F daughter bulb clearly showing the whorled-organized floral organ primordia including the central female organs or gynoecium. (**B**) Relative expression of potential tulip flower-meristem identity genes and floral homeotic genes at 32 WAP in F daughter bulbs compared to 32 WAP in vegetative NF daughter bulbs. (**C**) Heatmaps showing the expression profile of putative flowering-related genes in F and NF daughter bulbs. Colour scales indicate the regularized logarithm (rlog) transformed count values

At 32 WAP, flower organ primordia were visually detectable in the daughter bulbs originating from the centre of the mother bulb (F; Fig. 4A). On the contrary, the peripheral daughter bulbs remained vegetative and did not show any sign of flowering (NF, Fig. 4A). At the same time, multiple putative homologs of flower and flower organ identity genes were significantly and intensely upregulated in the transcriptome of F daughter bulbs in comparison to NF daughter bulbs (Fig. 4B). These genes are *FRUITFULL (TgFUL),* the putative floral meristem identity gene Tg*LFY,* and the potential flower organ identity determining homeotic genes *PISTILLATA (TgPI), SEPALLATA 2 and 3 (TgSEP2* and *TgSEP3)*. Accordingly, a transcriptomic switch between 24 and 32 WAP can be observed, signifying the moment of molecular transition to flowering in F daughter bulbs (Fig. 2B). Comparing the expression of putative flowering-time-controlling genes in F and NF daughter bulbs revealed no strong, qualitative differences (presence/absence), but rather quantitative differences that are in line with the bulb’s observable behaviour. Indeed, putative flowering inhibitors show prolonged or higher expression in NF bulbs. Such genes are for example *SHORT VEGETATIVE PHASE* (*TgSVP*), and *SOC1-like1* (*TgSOC1L1*), which because of its expression profile, contrary to that of its close homolog *SOC1-like2* (*TgSOC1L2*), is predicted to fulfil an antagonistic role in flowering [22]. Accordingly, putative flowering inducers such as *TgFD, TgFT4* and *SQUAMOSA-PROMOTER BINDING PROTEIN-LIKE1* (*TgSPL1*)*,* and markers of the flower transition such as *TgLFY* [22] are moderately higher expressed, or appear earlier, in F bulbs (Fig. 4C). Putative homologs of flower development genes such as *TgSEP1, SQUAMOSA (TgSQA)* and *GLOBOSA* (*TgGLO)* [22] are instead more strongly differential (Fig. 4B). The GO analysis revealed that from 35 WAP onward, genes associated with flowering and flower development are also found to be upregulated in NF bulbs (Supplementary File 1). However, among these, negative regulators are overrepresented (Supplementary File 2). Accordingly, GO terms related to the negative regulation of cellular metabolism and gene expression indicate a more general repressive state of NF meristems (Supplementary File 1). Based on these results, we can indicate that the difference in flowering capacity between daughter bulbs could be due to the repression of flowering capacity and metabolism in NF bulbs, rather than a specific flower induction in F bulbs.

### The potential role of tulip PEBPs in bulbing and flower induction

In plants, PEBP proteins regulate flower induction and storage organ formation by binding to the bZIP transcription factor FD and the TCP transcription factor TB1/BRC1 [7, 18, 28]. The latter interaction is of direct importance for the outgrowth of axillary buds in Arabidopsis [31], which is a process analogous to daughter bulb outgrowth in tulip, where an inverse correlation between bulb size and *TgTB1* expression has been reported (Natalia M. [27]. Inspired by this knowledge and the outcome of our comparative transcriptomics, we investigated the potential involvement of tulip *PEBPs* as regulators of bulb size and flowering in daughter bulbs by measuring their spatiotemporal expression and correlating it with changes in daughter-bulb morphology.

**Fig. 5:**
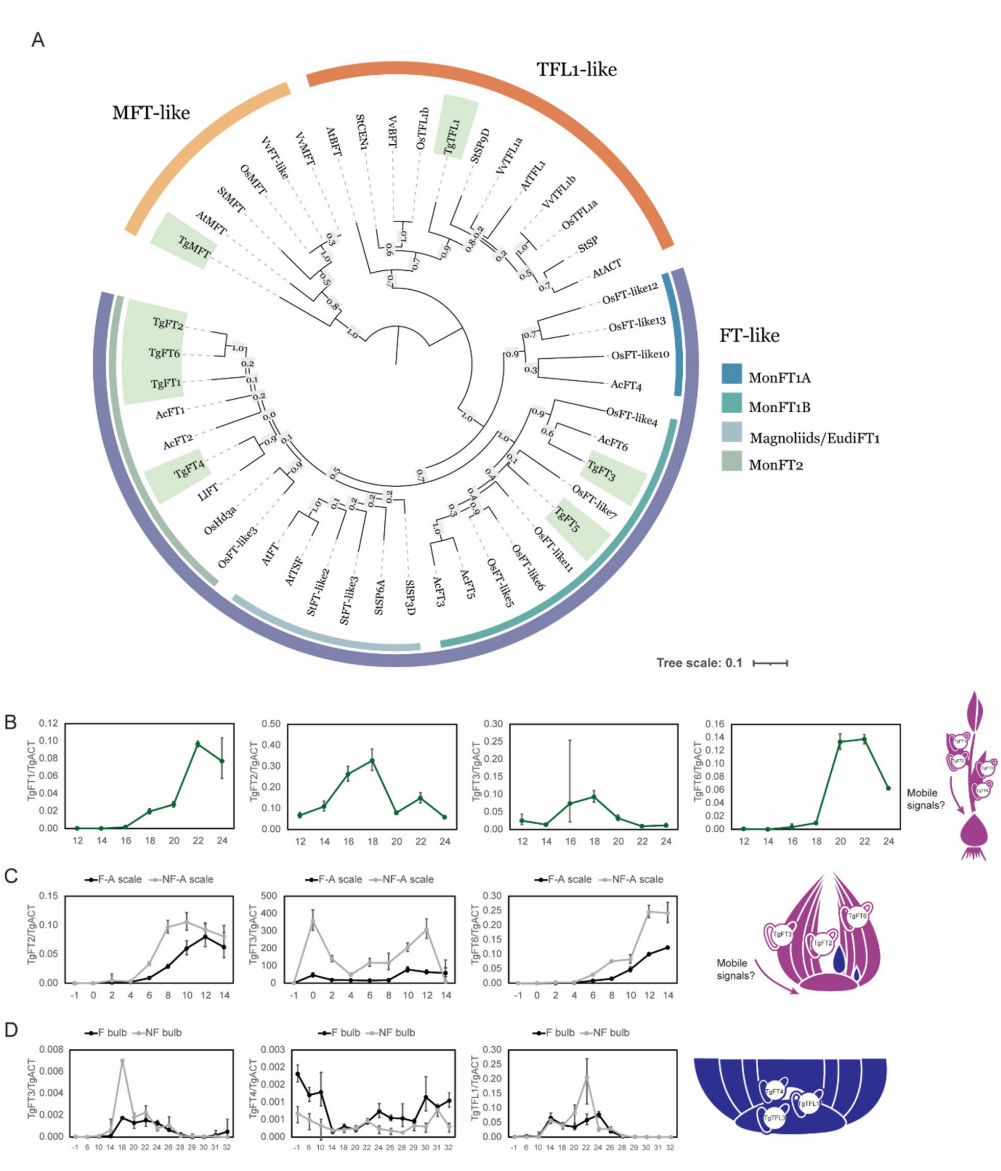
The PEBP family in *Tulipa gesneriana*. (**A**) ML phylogenetic tree of tulip PEBPs (highlighted in green) in comparison to other PEBP proteins of flowering plants. Abbreviations for the species names are as follows: *Ac* (*Allium cepa*), *At* (*Arabidopsis thaliana*), *Os* (*Oryza sativa*), *Sl* (*Solanum lycopersicum*), *St* (*Solanum tuberosum*), *Tg* (*Tulipa gesneriana*) and *Vv* (*Vitis vinifera*). Relative expression of *PEBP* genes in (**B**) leaves of the mother plant, (**C**) scales of the mother plant (F-A is the scale associated with the flowering bud; NF-A is the scale associated with the non-flowering bud), or (**D**) the meristematic area of daughter bulbs, which refers to the blue bulblets depicted in C. In the illustrations, the purple color indicates the mother plant, while blue indicates the daughter bulbs. (**B**-**D**) x-axis indicates the time in Weeks After Planting (WAP). Sampling of leaves (**B**) and scales (**C**) from the mother plant did not proceed after the indicated time points (24 and 14 WAP, respectively) because of the senescence of the material

The tulip *PEBP* genes *TgFT1, TgFT2, TgFT3, TgFT4* (previously *TgFT-like*), and *TgTFL1* have been partially characterized in relation to flowering and bulbing [22, 23]. Our detailed mining of available transcriptomic and genomic data allowed the identification of a few additional and previously unknown members of the tulip *PEBP* family. The updated PEBP family and phylogenetic tree counts six FT-like copies, TgFT1, TgFT2, TgFT3, TgFT4, TgFT5, TgFT6,one TFL1-like, TgTFL1, and one MFT-like protein, TgMFT (Fig. 5A). The novel sequence TgFT6 is part of the monocot-specific ‘Mon FT2’ division [33] with TgFT1, TgFT2, and TgFT4, and appears to be very similar to TgFT2 in terms of protein sequence (Supplementary Fig. 3). TgFT5, instead, falls into the ‘Mon FT1B’ division [33], together with TgFT3. Surprisingly, TgFT5 contains a histidine (H) in correspondence to the functionally relevant amino acid position that determines a positive or negative effect on flowering time for the Arabidopsis FT (Y85) and TFL1 (H88) proteins, respectively [12]. Therefore, TgFT5 is predicted to work as a repressor of flowering, like TFL1. Based on similarity and functional conservation within the *PEBP-gene* family, *TgMFT* is expected to be seed-specific. Indeed, this transcript could not be identified in transcriptomes that don’t include seed mRNA, while we were able to amplify and clone the supposed full-length *TgMFT* coding sequence from tulip seed cDNA (Supplementary Fig. 4). Therefore, we deemed its further characterization outside of the scope of this study.

Since these signals usually originate in the leaves, *PEBP* expression in tissues with leaf identity (leaves and scales) was investigated by qPCR. From the different identified *TgFT*-like genes, expression of *TgFT1*, *TgFT2, TgFT3,* and *TgFT6* was detected in the leaves of the mother plant (Fig. 5B) [23]. *TgFT2* and *TgFT3* are both expressed around and during the blooming of the mother plant (between 14 and 20 WAP), albeit *TgFT2* to a higher degree. *TgFT1* and *TgFT6* leaf expression partially overlap with *TgFT2* and *TgFT3* starting at a later moment, from blooming onwards (18 WAP and 20 WAP, respectively). Furthermore, *FT-like* gene expression was measured in the mother bulb scales, which are modified leaves. Each scale is directly connected to a single daughter bud, and it can in principle be the source of differentiated growing signals for the buds. From the analyzed *TgFT*-like genes, *TgFT2*, *TgFT3,* and *TgFT6* expression were detected in scale tissue, in the scale connected to the fast-growing F bud (named ‘F-associated scale’,F-A scale) as well as in the scale connected to the slow-growing NF bud (named ‘NF-A scale’), and all three appear higher in NF-A scales (Fig. 5C). What differentiates these patterns of expression is both timing and magnitude: in fact, *TgFT2* is expressed from 6 WAP until 14 WAP and peaks at 10 WAP; *TgFT6* is detected in the same timeframe, but peaks at 12–14 WAP; finally, *TgFT3* was found to be expressed soon after the mother bulbs were planted in soil (one day after planting), and to a much higher degree in NF-A scales in comparison to F-A scales. As for most FT proteins described in the literature, TgFT2, TgFT3, and TgFT6 could be transported from leaf-like organs (in this case the scales of the mother bulb) to a meristem (in this case the developing axillary daughter buds), and therefore could contribute to the regulation of daughter bulb growth and development. Considering its differential expression profile in scales, *TgFT3* is of particular interest as a potential source of differential regulation between daughter buds.

Local expression of *TgFT4* and *TgTFL1* has been reported in meristem-rich samples of F and NF daughter bulbs and has been previously linked with flowering regulation in tulip [22] (Fig. 5D). According to our results, *TgTFL1* shows a window of expression between 14 and 26 WAP. Although it is present in both types of daughter bulbs, expression in NF is higher at 20 and 22 WAP. The expression of *TgFT4* is roughly opposite to the one of *TgTFL1*,it is high between 0 and 10 WAP and after 24 WAP. *TgFT4* mRNA appears to be overall moderately more abundant in F daughter bulbs, supporting the hypothesis that it acts as a flower inducer. The fact that *TgFT4* is not only expressed at flower induction as expected but also at very early stages of bud development (between 0 and 10 WAP), points to additional roles and suggests a function associated with meristem activity. Moreover, *TgFT3* was detected mainly in NF daughter meristem-enriched samples, with a peak at 18 WAP.

### Differential protein complex formation capacity of tulip PEBPs and activity upon ectopic expression in Arabidopsis

**Fig. 6:**
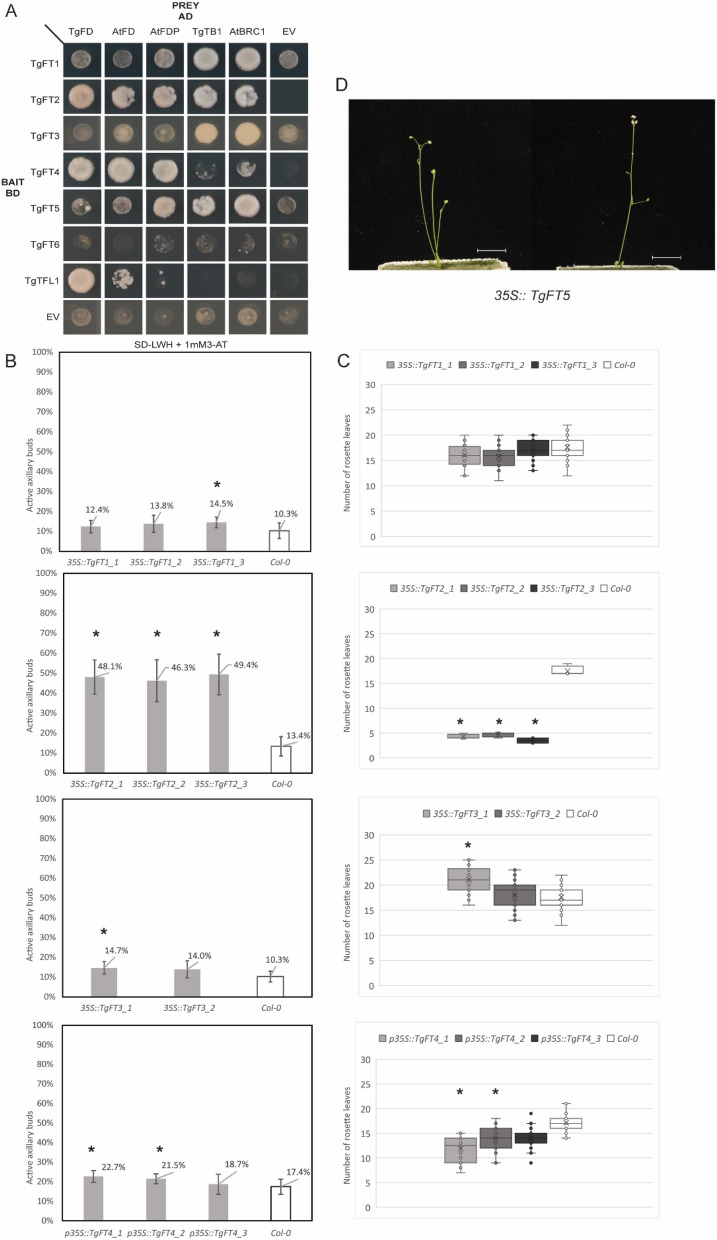
Biological activity of selected tulip PEBPs. (**A**) Y2H assay showing the protein–protein interaction capacity of tulip PEBPs with tulip and Arabidopsis FD and TB1/BRC1-like proteins. EV is a negative control based on the introduction of an Empty AD or BD vector. Full Y2H data can be found in Supplementary Fig. 7. (**B**) Percentage of active rosette axillary buds in Arabidopsis lines constitutively expressing *TgFT1*, *TgFT2*, *TgFT3,* or *TgFT4*. Plants were scored at the end of their lifecycle, at the first visible signs of silique browning. (**C**) Flowering time of Arabidopsis lines constitutively expressing *TgFT1*, *TgFT2*, *TgFT3,* or *TgFT4*, measured in the number of rosette leaves at visible bolting. (**D**) Morphology of Arabidopsis lines constitutively expressing *TgFT5*, at the stage of anthesis. The white bar indicates 1 cm. Asterisks indicate p < 0.05 in pairwise T-test assuming equal variance

To provide more insight into the potential functions of the different tulip PEBPs and to select the strongest candidates as regulators of flowering and bulbing, we analyzed their protein–protein interaction capacity by yeast two-hybrid (Y2H). The full-transcript sequences of the putative *FD* homolog *TgFD* (newly identified from transcriptomic data) and *TgTB1* (previously available in partial sequence) were retrieved from transcriptomic data (Supplementary Figs. 3 and 6) and used in a protein–protein interaction assay. Their Arabidopsis counterparts were used as a control. We hypothesized that PEBPs with florigen or anti-florigen activity might interact with TgFD, whereas PEBPs playing a role in the outgrowth of daughter bulbs probably would interact with TgTB1. Our results revealed the interaction of TgFT2, TgFT4, and TgTFL1 with TgFD; while TgFT1, TgFT2, TgFT3, and TgFT5 interacted with TgTB1 (Fig. 6A). Interestingly, TgFT2 is the only tested case of binding with both FD and TB1, mimicking the binding capacity of the Arabidopsis FT. Despite its high sequence similarity with TgFT2, TgFT6 did not show interaction with either of the transcription factors and was for this reason excluded from further analysis. The other tested PEBPs showed interaction specificity, suggesting some degree of specialization.

The ability of tulip PEBPs to specifically bind to TgFD or TgTB1 in Y2H suggests their potential capacity to affect their partner’s activity. To explore the potential role of these PEBP proteins in flowering and bulb size control, and considering the analogy between axillary bulb outgrowth in tulips and branching in Arabidopsis (Natalia M [27], we employed a heterologous approach and produced Arabidopsis lines expressing the selected tulip *PEBP* genes under a constitutive promoter. Number of rosette leaves was used as an indicator of flowering time. The percentage of active axillary branches was scored by counting the number of branches originating from the rosette leaves at the time point that the first silique on the main inflorescence starts browning and hence, ripening. For this purpose, Arabidopsis Col-0 lines ectopically expressing either *TgFT1*, *TgFT2*, *TgFT3*, *TgFT4,* or *TgFT5* under the constitutive CaMV35S promoter (35S) were generated and ectopic expression was confirmed by qRT-PCR (Supplementary Fig. 6). For *TgFT1,* only *35S::TgFT1* line 3 showed a significant effect in the percentage of active axillary branches, while none of the three lines showed a significant flowering time phenotype, in contrast to the previously observed mild late flowering response upon ectopic expression of *TgFT1* [23]. *35S::TgFT2* lines, instead, showed a clear increase in the outgrowth of axillary branches, as well as a significant early flowering phenotype for all three independent transgenic lines. A similar flowering-inducing effect was found for this tulip gene in the previous study [23]. For the one *35S::TgFT3* line where higher expression of *TgFT3* was observed (Supplementary Fig. 6), we could report an increase in axillary branches, and a late flowering phenotype, in line with the previous publication [23]. Among the *35S::TgFT4* lines, two had a minor increase in axillary branches and flowered significantly earlier than Col-0 (Fig. 6B and C). Intriguingly, *35S::TgFT5* lines showed extremely early flowering, completely overcoming the vegetative phase, with no rosette leaves produced. This is surprising since TgFT5 is predicted to have a repressive function due to its TFL-like amino acid residue corresponding to Y85/H88 (Supplementary Fig. 3). Unfortunately, due to the extreme early flowering, we couldn’t retrieve viable seeds from these lines and therefore, these plants were characterized at the T1 generation only. Consequently, no data on axillary meristem outgrowth could be obtained.

As our functional characterization suggests, heterologous expression of tulip *PEBPs* in Arabidopsis can help to discern between those that have a clear effect on flowering time and those that don’t. On the other hand, effects on branching are less pronounced and seem to be mostly linked with flowering effects.

## Discussion

### The transcriptional trajectory linked to the differential daughter bulb development

In the process of vegetative reproduction in adult tulip bulbs, axillary buds are initiated between the scales of the mother plant and develop into daughter bulbs of different sizes and with different flowering capacities. In the current study, we compared the transcriptomic profiles of large, flowering (F) daughter bulbs and small, non-flowering (NF) daughter bulbs from the same bulb cluster during their development from primordia to mature dormant bulbs. At first glance, both bulb types seem to follow a similar transcriptional trajectory, which can be divided into three phases (Fig. 2B): the first one, between -1 and 10 WAP, can be associated with the timeframe where the daughter bulb primordia are kept in an apparently dormant state in the form of buds (Fig. 2A), and don’t gain weight (Natalia M. [27],the second one, between 18 and 24 WAP, coincides with the growth phase, when both daughter bulbs gain weight, although to very different extents (Fig. 2A; Natalia M. [27],the third one, between 32 and 37 WAP matches with the period of bulb maturation. In this period there is no substantial weight gain but other processes take place, such as the formation of the tunica (the outmost protective layer of the bulb) in NF and F daughter bulbs and flower development in F daughter bulbs (Fig. 2A and 4A). In parallel, in this third period, bulb dormancy is established in all daughter bulbs contained in the cluster [22]. Within these three periods though, many transcriptomic differences were detected that can explain the developmental differences that clearly distinguish F and NF daughter bulbs. Indeed, we detected DEGs related to growth and proliferation by comparing the transcriptomes of F and NF axillary buds as early as 10 WAP (Fig. 3A and B). This is the last time point of the first period when neither of the daughter bulbs has started to visually grow, but clearly, transcriptional regulation of genes potentially associated with cell division is different. This contrasting activity is in line with the differential weight gain capacity of daughter bulbs, as described in the literature (Natalia M [27], and which is reflected in the period immediately following 10 WAP.

### Slow-growing daughter bulbs remain vegetative

F and NF daughter bulbs are developing in the same bulb cluster and receive the same flower-inducing environmental conditions. Nevertheless, the slow-growing NF daughter bulbs do not make the switch to flowering (Fig. 2A and 4A), which could be due to insufficient nutrient accumulation and because of selective molecular inhibition. F daughter buds are the first to resume growth after a period of semi-dormancy (Fig. 2A) [27]. As known, actively proliferating tissues become strong sinks [4, 16], and therefore are strong competitors for later-developing NF daughter bulbs. Indeed, higher TOR kinase activity during cold application (Fig. 3C) suggests a higher metabolic status in F buds, which is associated with a stronger growth response. As a consequence, NF daughter bulbs remain weaker sinks and receive smaller amounts of photoassimilates but also of potential mobile developmental regulators, such as different PEBP proteins. The difference in timing and extent of dormancy release is likely to be molecularly regulated. In fact, the expression of *TgTB1* is highly correlated with growth capacity (Natalia M. [27] (Supplementary Fig. 2). Accordingly, we observed that expression of flowering repressor genes and cell metabolism-associated genes was elevated in NF bulbs. Based on our experimental results, we hypothesize that mobile PEBP signals originating from the mother plant regulate dormancy release and growth capacity at daughter buds, which results in the different sizes. Specifically, leaf-borne signals such as TgFT1 and TgFT2 could induce bulb outgrowth through interaction and inhibition of TgTB1. When considering that the fast-growing F daughter bulb is likely to become a strong sink organ, we can hypothesize that these systemic signals from the mother’s leaves can reach the F daughter bulb in a higher amount in comparison to the NF daughter bulb and contribute to its development. In parallel, the scales of the mother plant could be the source of differential inhibitory signals, such as *TgFT3*, which is expressed higher in NF-A scales and NF meristems (Fig. 5C and D). In line, *TgFT3* ectopic expression in Arabidopsis does not accelerate flowering (Fig. 6C). We hypothesize that TgFT3 could constitute an ‘anti-bulbigen’ that impedes the full development of NF daughter bulbs by binding TgTB1 without inhibiting its activity (Fig. 6A). Complex and balancing acts between functionally different PEBPs as proposed here for tulip have been described also in the bulbous species onion, with a role for AcFT2 as florigen, AcFT1 as a bulbigen, and AcFT4 as an anti-bulbigen (Lee, Baldwin, Kenel, Mccallum, et al*.*, 2013). In analogy to this situation in bulbous species, a balancing PEBP mechanism was described in strawberry, a species that also has a dual reproduction modus with sexual reproduction via flowers and seeds, and asexual reproduction via so-called runners developing from axillary meristems [10]. Flowering in strawberries is controlled by the florigen FveFT2 and the systemically acting anti-florigen FveTFL1. In addition, a third PEBP protein is produced, FveFT3, which is supposed to determine the developmental fate and growth capacity of the axillary buds. Overall, these data and observations suggest a key and conserved function for PEBP proteins in determining the developmental fate and growth of (axillary) meristems in plants with a dual reproduction strategy. Furthermore, nutrient scarcity seems to play a role in the lack of flowering competence in tulip daughter bulbs. It has been shown that sugar sensing is a determinant for the success of flower induction in Arabidopsis, mediated by the signalling molecule TREHALOSE-6-PHOSPHATE (T6P) [39]. Following this theory, even though both F and NF daughter bulbs may receive other florigenic signals, their final flowering capacity would be determined very early on by their growth capacity and sink strength.

### Florigen in tulip

Because of their protein–protein interaction with TgFD in Y2H assays, their accelerating effect on flowering when ectopically expressed in Arabidopsis (Fig. 6C) [23], and their enhanced expression associated with the flowering of F daughter bulbs [22, 23], both *TgFT2* and *TgFT4* could potentially constitute the tulip florigen. Nevertheless, the leaves of the mother plant are senescent around the time of flower induction in F daughter bulbs (32 WAP) (Fig. 2A). For this reason, we point to the daughter-bulb expressed *TgFT4* as a more suitable candidate for the role of florigen. Some additional examples of potential meristematic florigens are found in other geophytes, such as the Narcissus *NtFT* and the lily *LlFT* [23, 32]. Furthermore, in rice local production and functioning of an FT-like protein in the meristem has recently been verified [11], showing that this mode of action exists in nature. In our case, there is no proof that *TgFT4* is expressed in meristematic cells only and not at the leaf primordia that surround it,if the latter were the case, a short-distance transmission would still be necessary.

## Conclusions

**Fig. 7:**
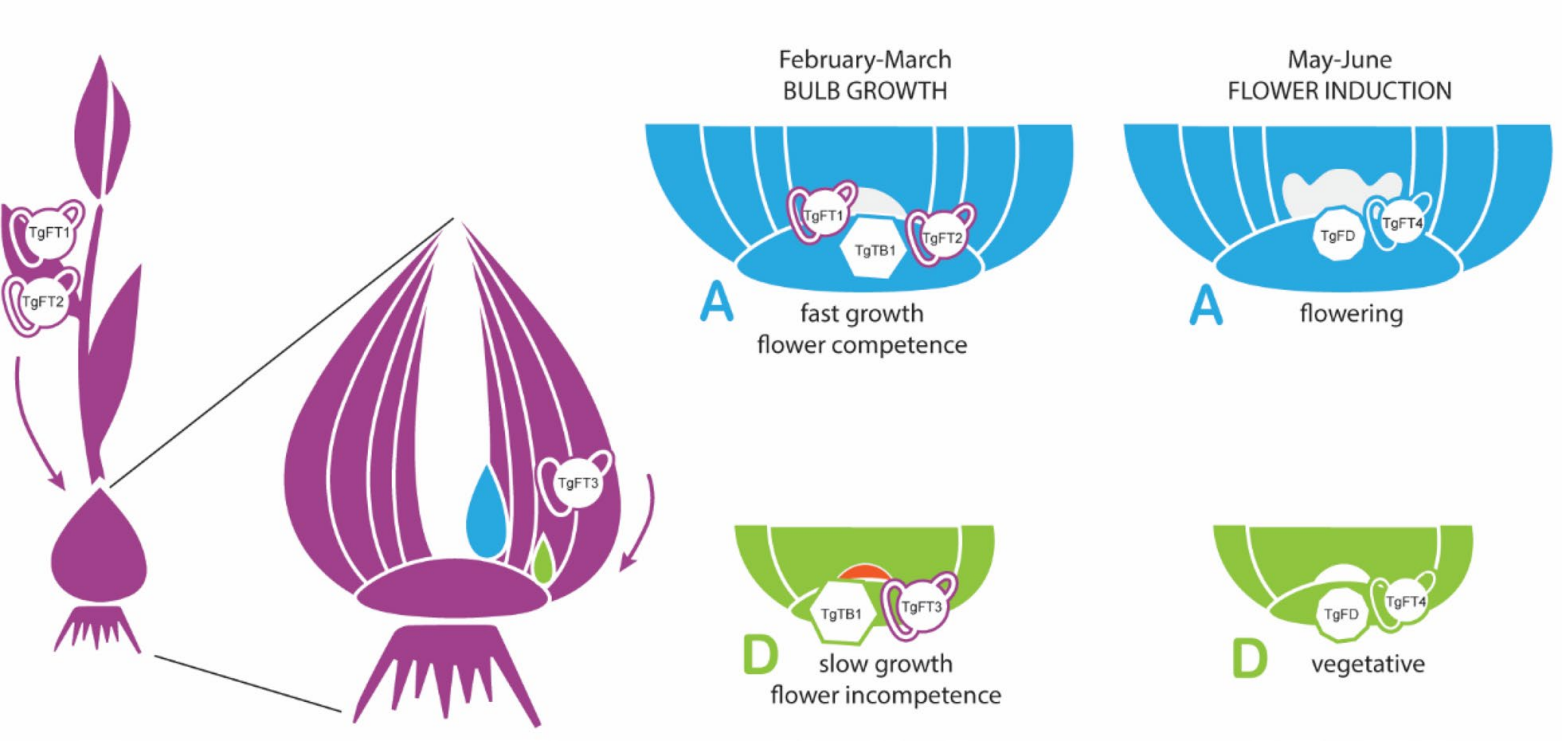
Summarizing model of PEBP protein functioning in tulip. Different colours indicate either the mother plant (in purple), the flowering potential (F) A-class daughter bulb (in blue) and the non-flowering (NF) D-class daughter bulb (in green). According to our model, the inducive signals TgFT1 and TgFT2 are produced by the mother plant leaves and reach the F daughter bulb in higher amounts because of differential sink strength. Once they have reached the meristematic region of F daughter bulb, TgFT1 and TgFT2 bind and inhibit the axillary growth inhibitor TgTB1, and promote growth and development. In parallel, the repressor TgFT3 is abundantly produced in the mother bulb scale connected with the NF daughter bulb, which therefore receives it in high amounts. Although TgFT3 binds to TgBRC1, it is not able to inhibit its activity; therefore, the NF daughter bulb remains in a dormant state for a longer time. As a consequence, the F daughter bulb grows faster and acquires flower competence, whereas the NF daughter bulb grows slower and does not acquire flower competence. Subsequently, even though both daughter bulbs produce the TgFD-binding, flower-inducing signal TgFT4, only the F daughter bulb can accomplish flower development

A comparison of the transcriptomic profiles of F and NF daughter bulbs during their seasonal development revealed a surprising similarity, considering their differential growth and flowering potential. In search for regulators of bulb development, we further characterized the tulip PEBP family and identified TgFT1, TgFT2, and TgFT3 that could be received in different amounts by F and NF daughter bulbs and could have the potential to regulate their developmental fate and outgrowth. Finally, we proposed that the main florigen in tulip is TgFT4, which originates directly in the daughter bulbs. Nevertheless, the link between TgFT2 and flowering cannot be excluded. Although our study strongly points to a certain functional role for some tulip PEBPs (summarized in Fig. 7), only the development of mutant lines in tulip can prove such association-based claims. Advancement of current technology is required to overcome the recalcitrance of this species and allow such studies to be performed.

## Supplementary Information

Supplementary Material 1.

Supplementary Material 2.

Supplementary Material 3.

## Data Availability

The RNAseq datasets generated and analyzed during the current study are available in the NCBI’s Sequence Read Archive (SRA) repository, BioProject PRJNA777886.
