# Supplementary material for: Differential growth and flowering capacity of tulip bulbs and the potential involvement of PHOSPHATIDYLETHANOLAMINE-BINDING PROTEINS (PEBPs)
Source: Biol Direct. 2025 Mar 10;20:29. doi: 10.1186/s13062-025-00625-y (PMC11895272; doi:10.1186/s13062-025-00625-y)
Supplement: Supplementary file 1 — Supplementary Material 1. [file 13062_2025_625_MOESM1_ESM.docx]

# Supplementary Material

**
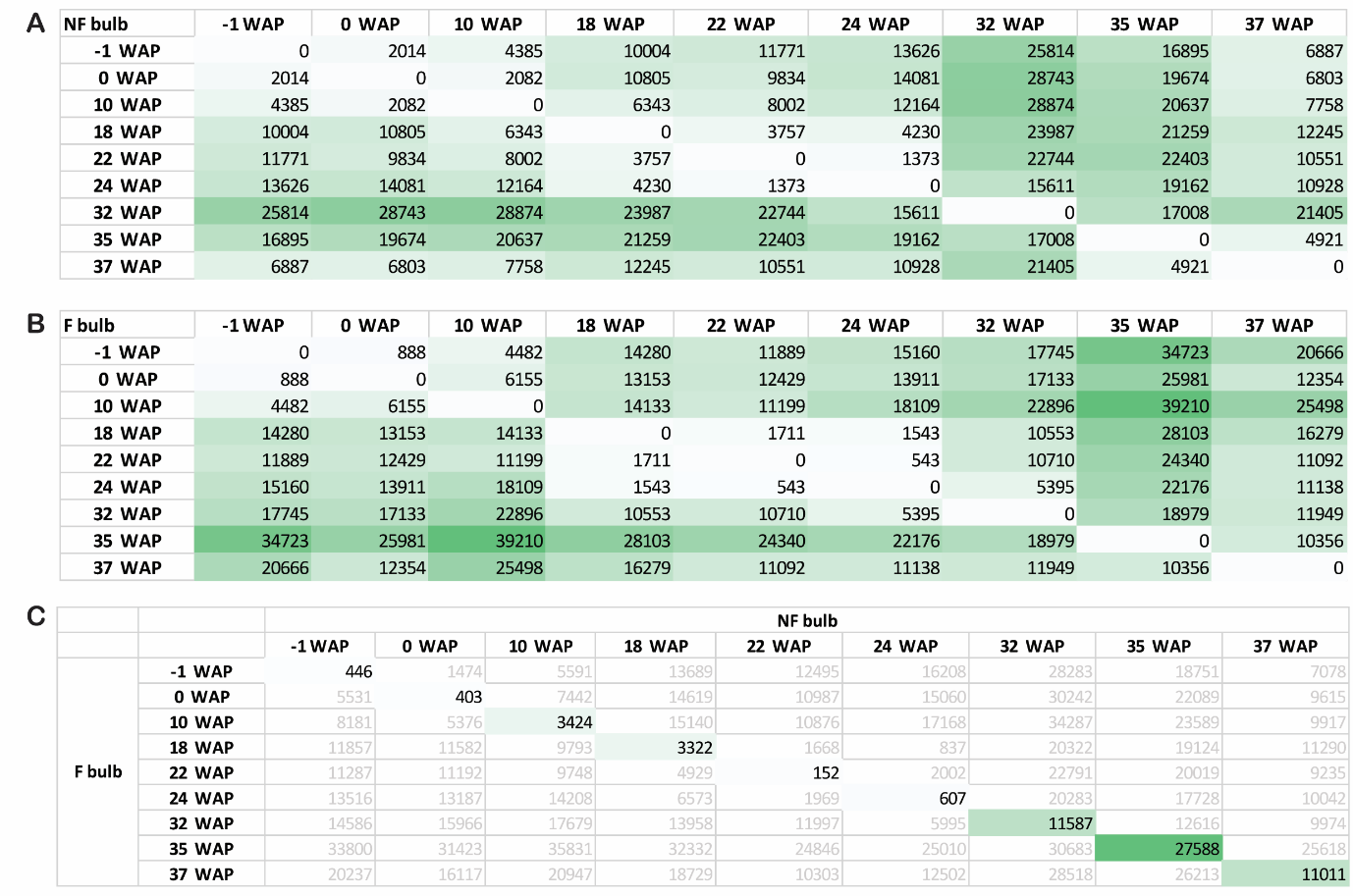
**

**Supplementary Figure 1. DEG analysis.** Number of DEG genes in pairwise comparisons of all time points in NF **(A)** and F **(B)** bulbs. **(C)** pairwise comparison between NF and F bulbs at every time point. WAP = Weeks After Planting.

**
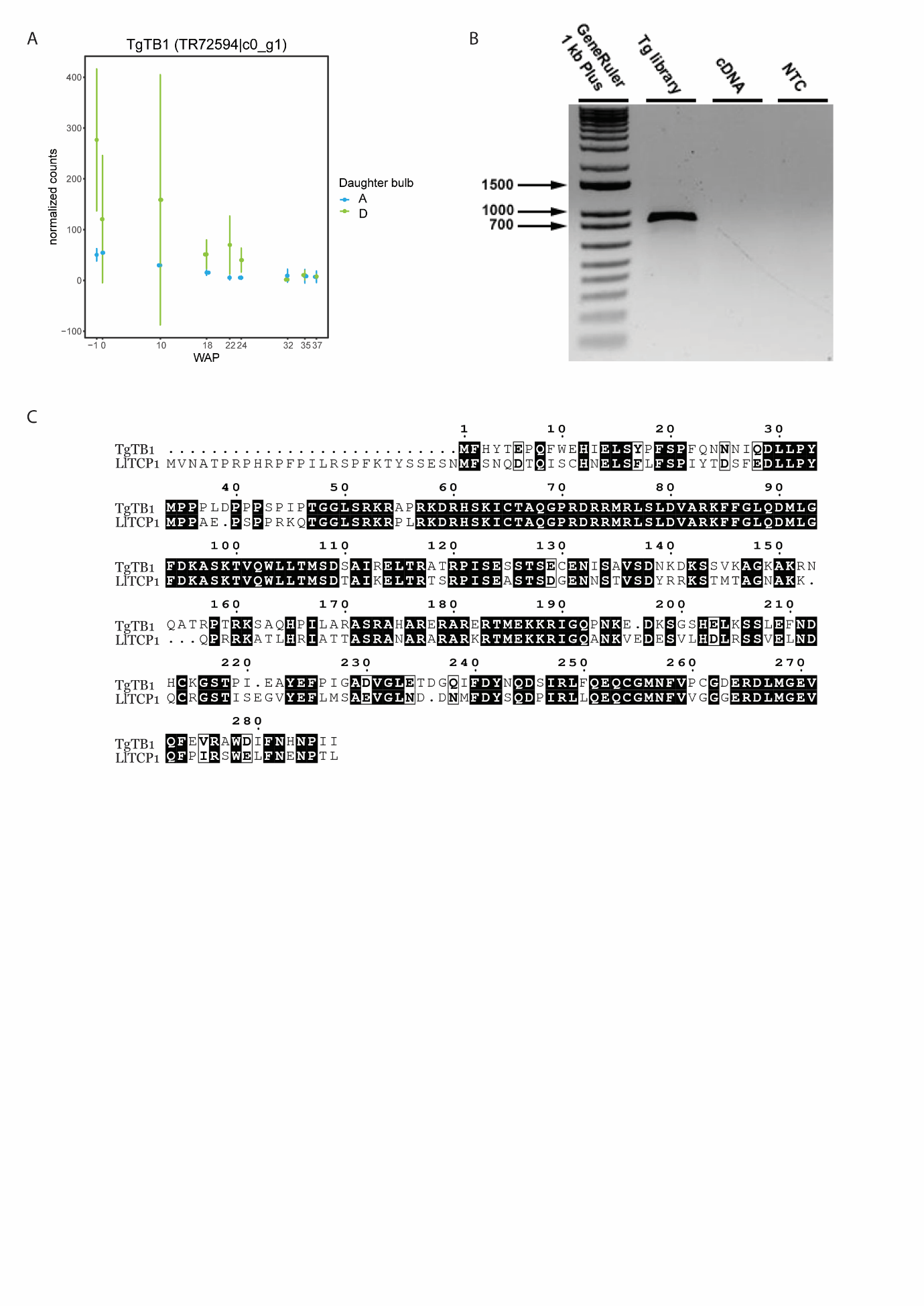
Supplementary Figure 2. TgTB1 characteristics**. **(A)** *TgTB1* expression in flowering (F) A-class and non-flowering (NF) D-class daughter bulbs **(B)** The predicted *TgTB1* CDS was experimentally validated by PCR. The CDS was amplified using a tulip cDNA expression library as a template (indicated as Tg library). Primers are shown in Supplementary Table 1. **(C)** Protein sequence alignment of predicted TgTB1 and LlTCP1 (*Lilium longiflorum*), showing that TgTB1 has a shorter N-terminal sequence compared to LlTCP1.


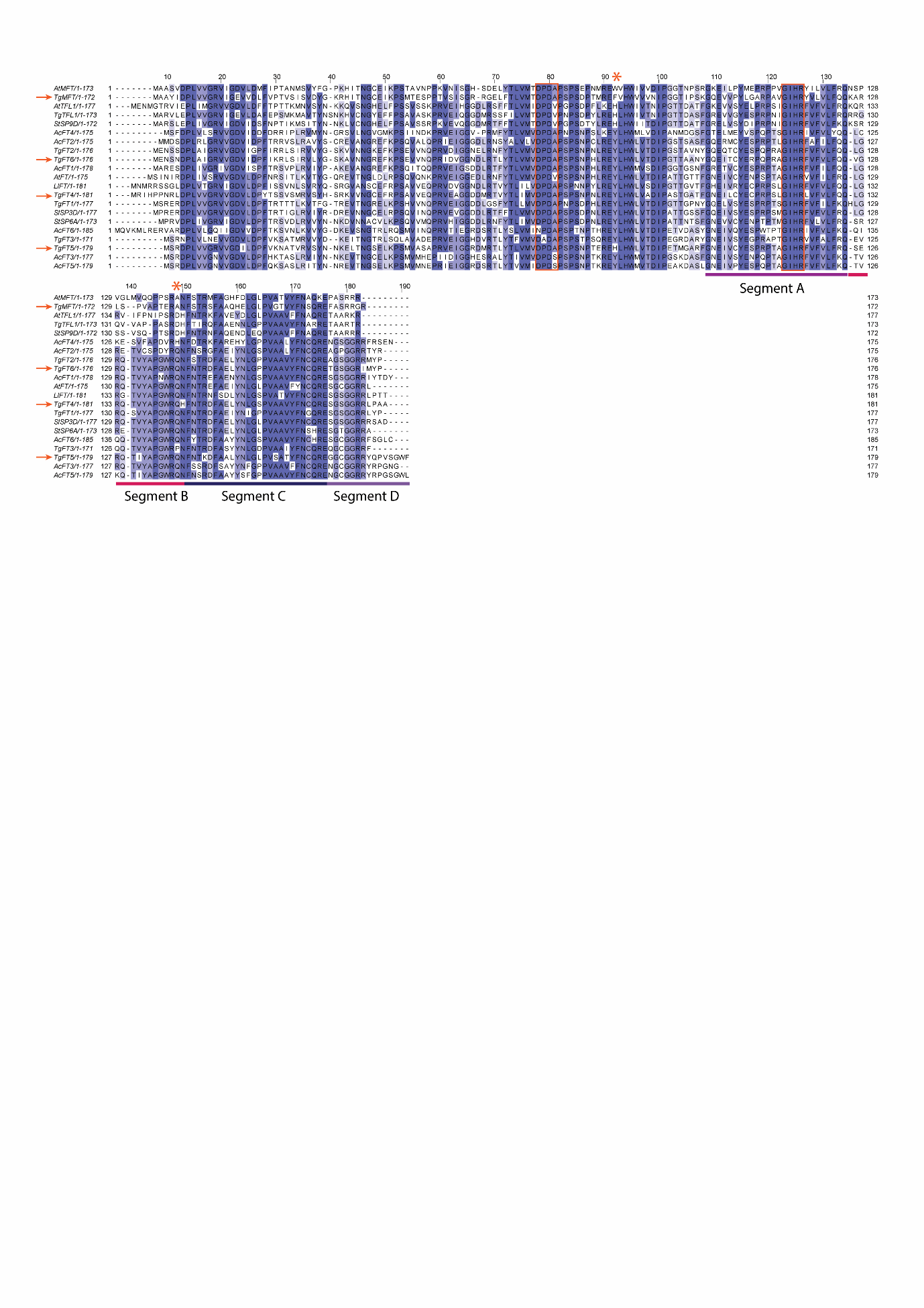


**Supplementary Figure 3. PEBP protein alignment.** Protein alignment of newly identified tulip PEBPs (orange arrows). The blue shading indicates the level of conservation of each amino acid (darker colours indicate higher conservation). Orange boxes and asterisks mark highly conserved motifs and functionally relevant residues, respectively.


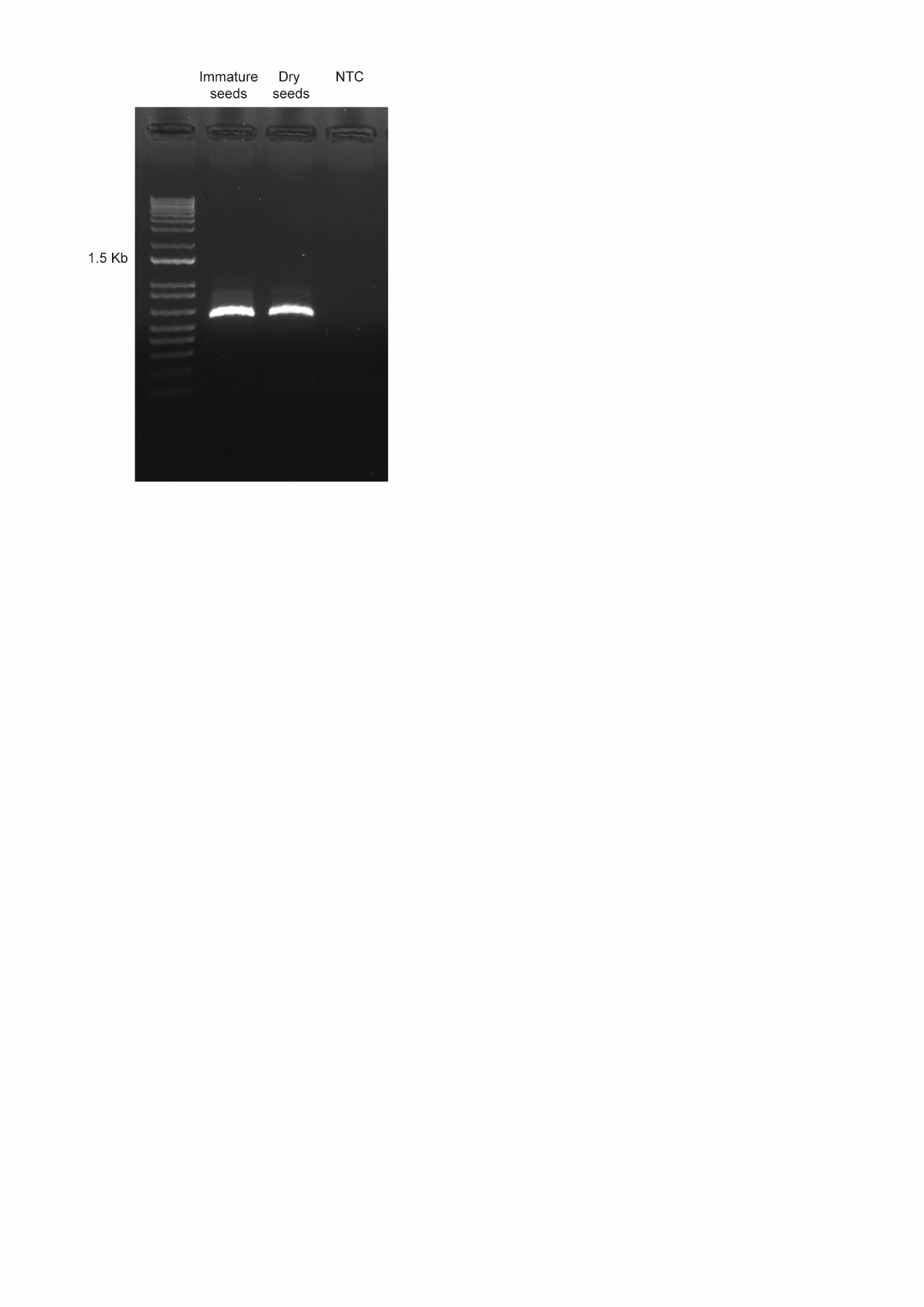
**Supplementary Figure 4. Amplification of *TgMFT* using seed cDNA as a template.** NTC = Non-Template Control. Primers are listed in Supplementary Table 1.


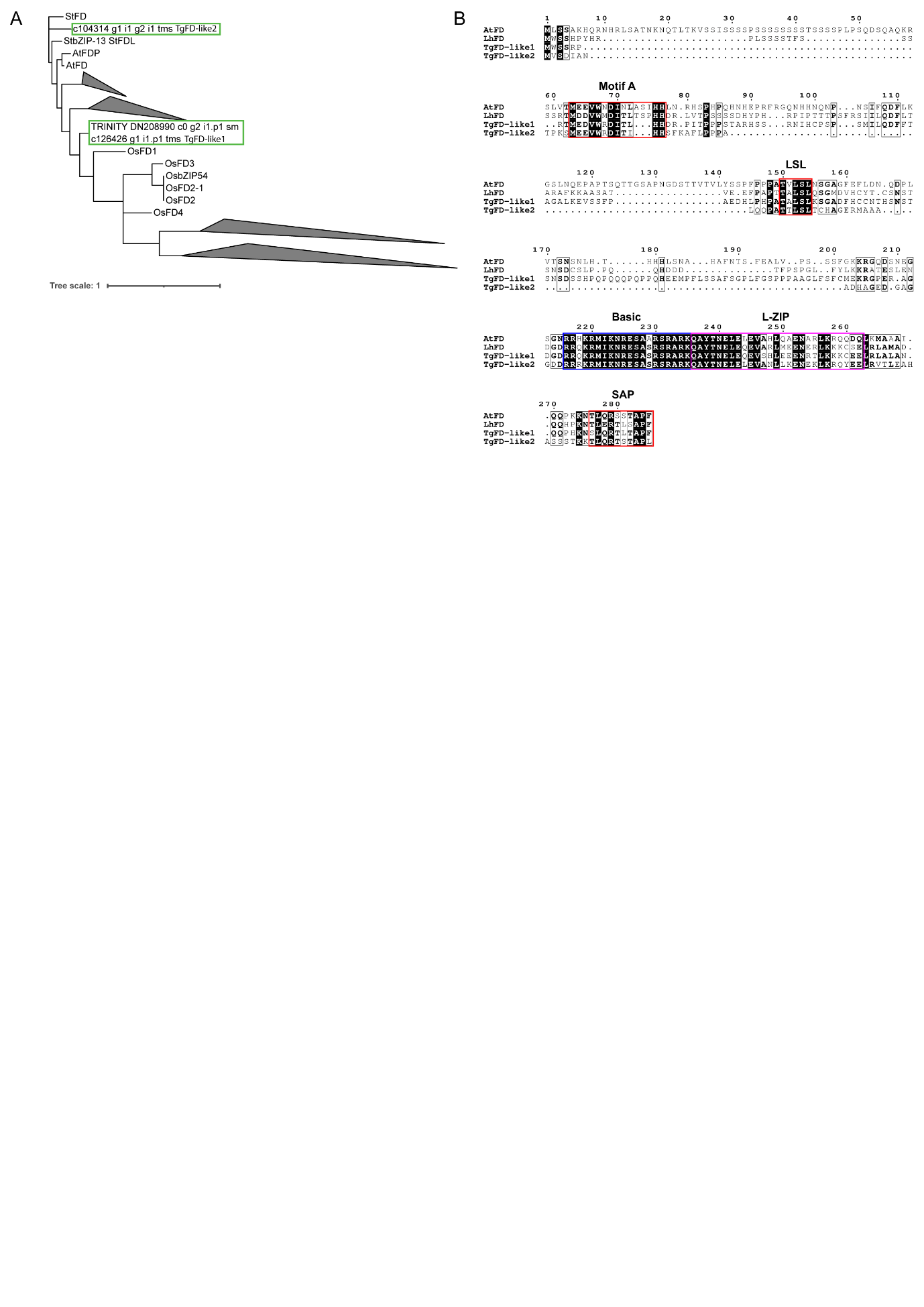
**Supplementary Figure 5. Identification of putative *FD-like* genes in tulip. (A)** maximum-likelihood trees constructed using the Arabidopsis, Rice, Potato, and tulip bZIPs. The tulip sequences in green boxes have been selected as candidates. **(B)** Protein sequence alignment of the Tulip FD-like sequences with AtFD and LhFD (*Lilium sp.*). Conserved motifs are marked with boxes. *TgFD-like1* has been selected for characterization and named *TgFD* in this study.


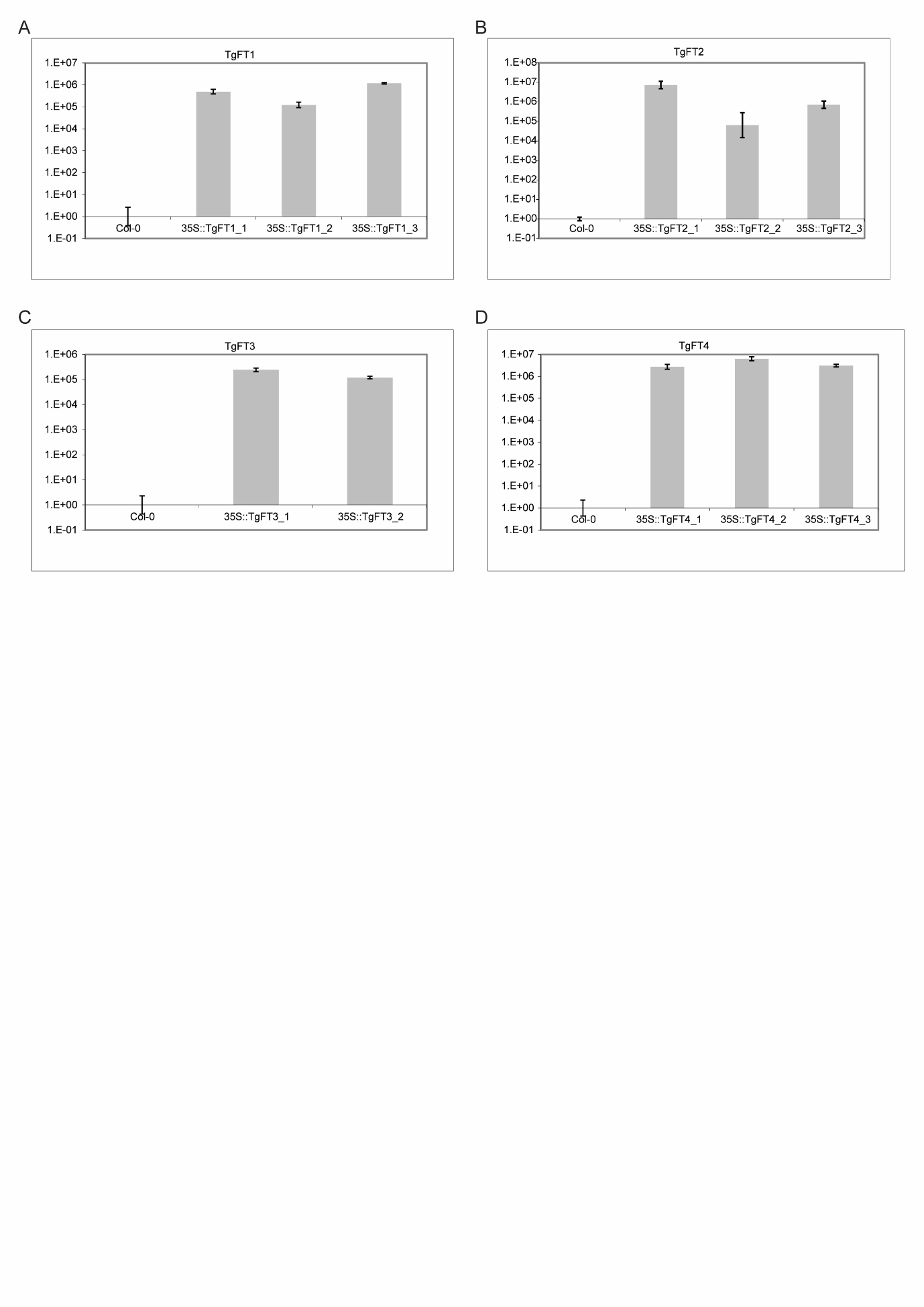
**Supplementary Figure 6. Validation of ectopic gene expression in Arabidopsis transgenic lines. (A-D)** qPCR analysis of tulip transgenes in Arabidopsis lines. The values indicate relative expression in comparison to the Arabidopsis housekeeping gene *TIP41*.


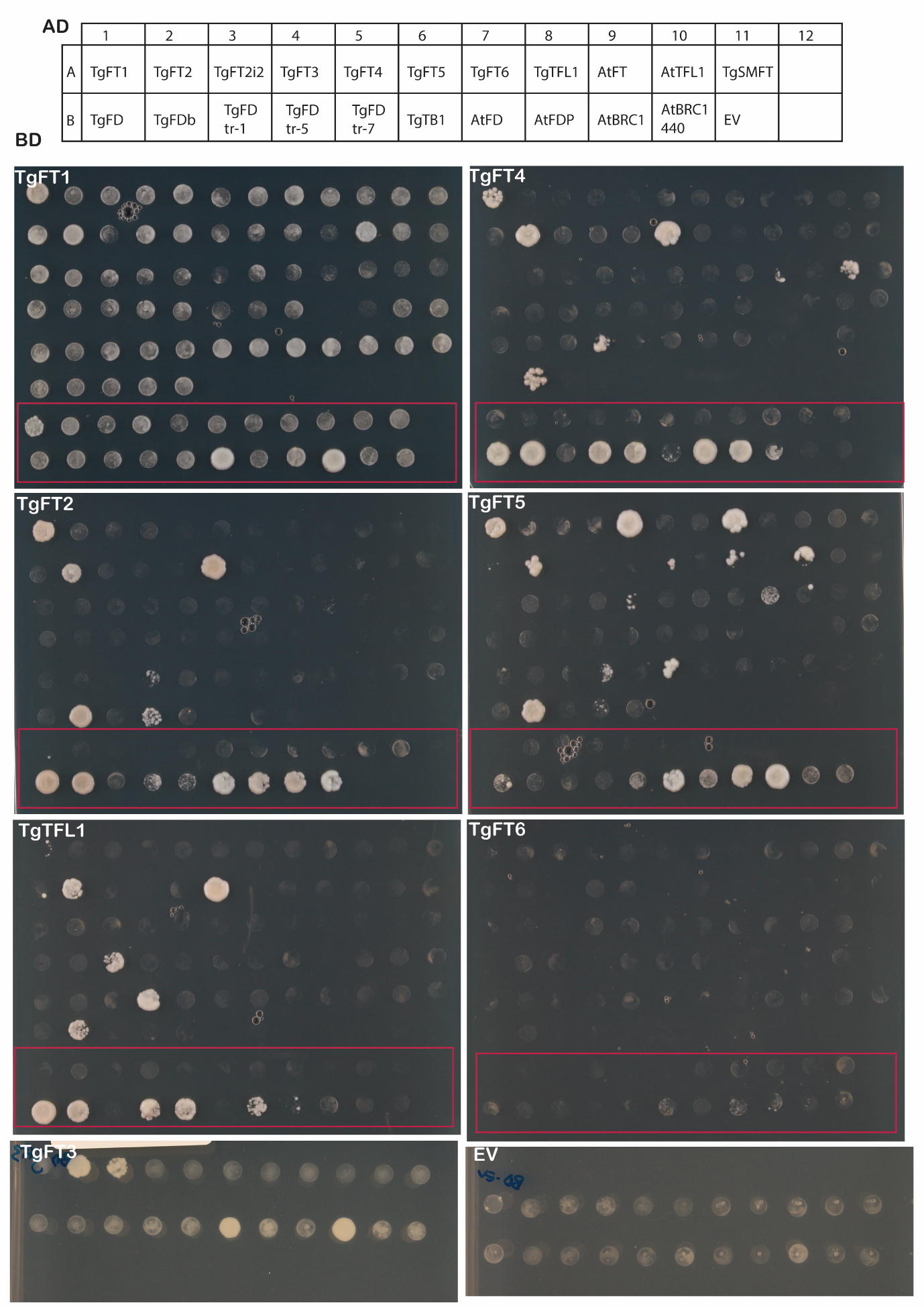


**Supplementary Figure 7. Pictures of full Y2H plates, including layout.**


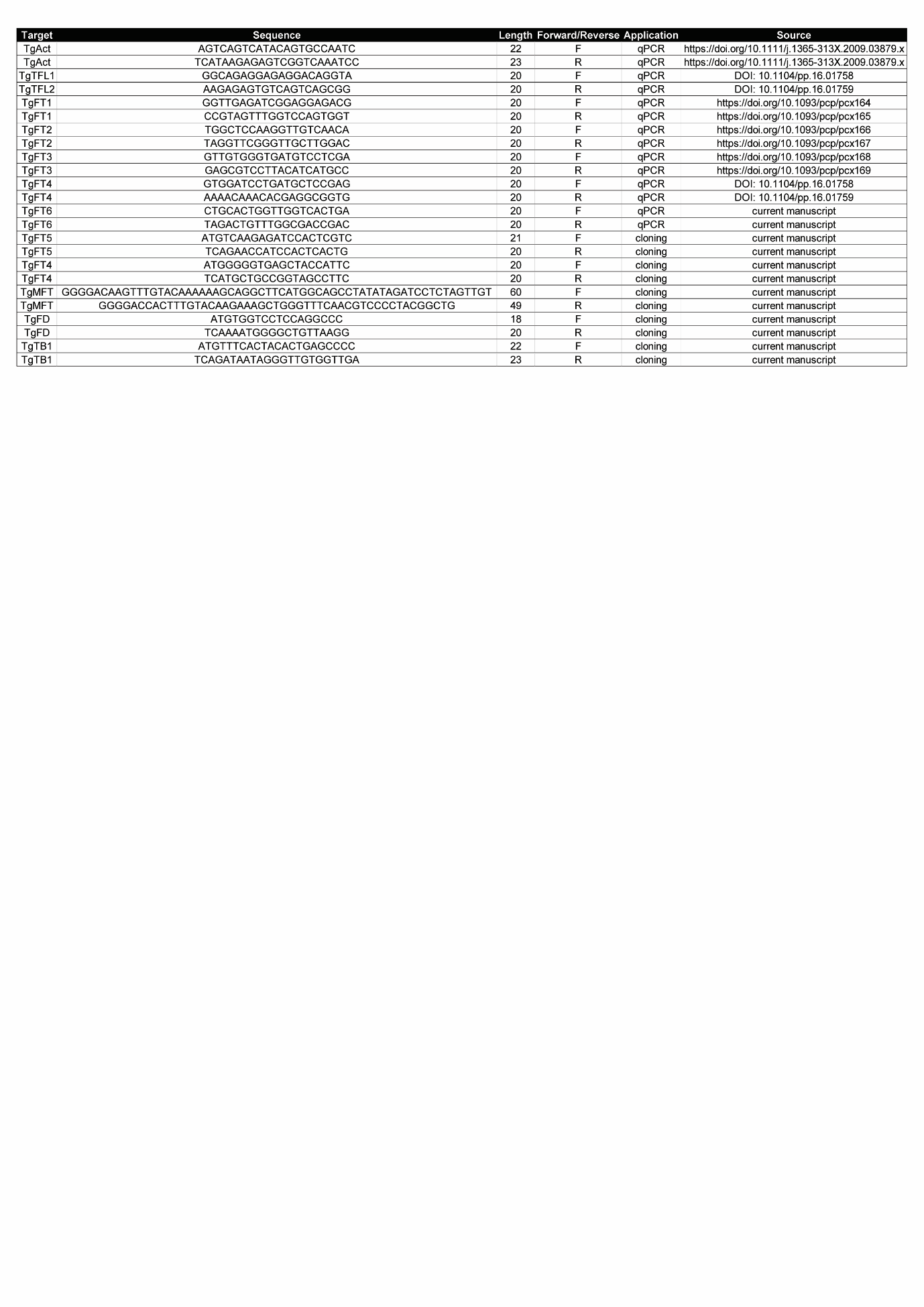
**Supplementary Table 1.** Primers used in this study.
