# Supplementary material for: Differential growth and flowering capacity of tulip bulbs and the potential involvement of PHOSPHATIDYLETHANOLAMINE-BINDING PROTEINS (PEBPs)
Source: Biol Direct. 2025 Mar 10;20:29. doi: 10.1186/s13062-025-00625-y (PMC11895272; doi:10.1186/s13062-025-00625-y)
Supplement: Supplementary file 2 — Supplementary Material 2. [file 13062_2025_625_MOESM2_ESM.pdf]

Supplementary File 1. Pairwise GO analysis of up- and down-regulated genes. Each plot name indicates which transcriptomes are compared (Number of WAP = Weeks After Planting; F = Flowering bulbs, NF = non-flowering bulbs; the < and > signs show which of the two samples has the higher expression). In each plot, the top 25 enriched GO terms are shown. Differential gene expression was performed using the Wald test ($p < 0.05$). Annotations are based on the closest Arabidopsis gene (Blastp, e-value $< 10^{-5}$).

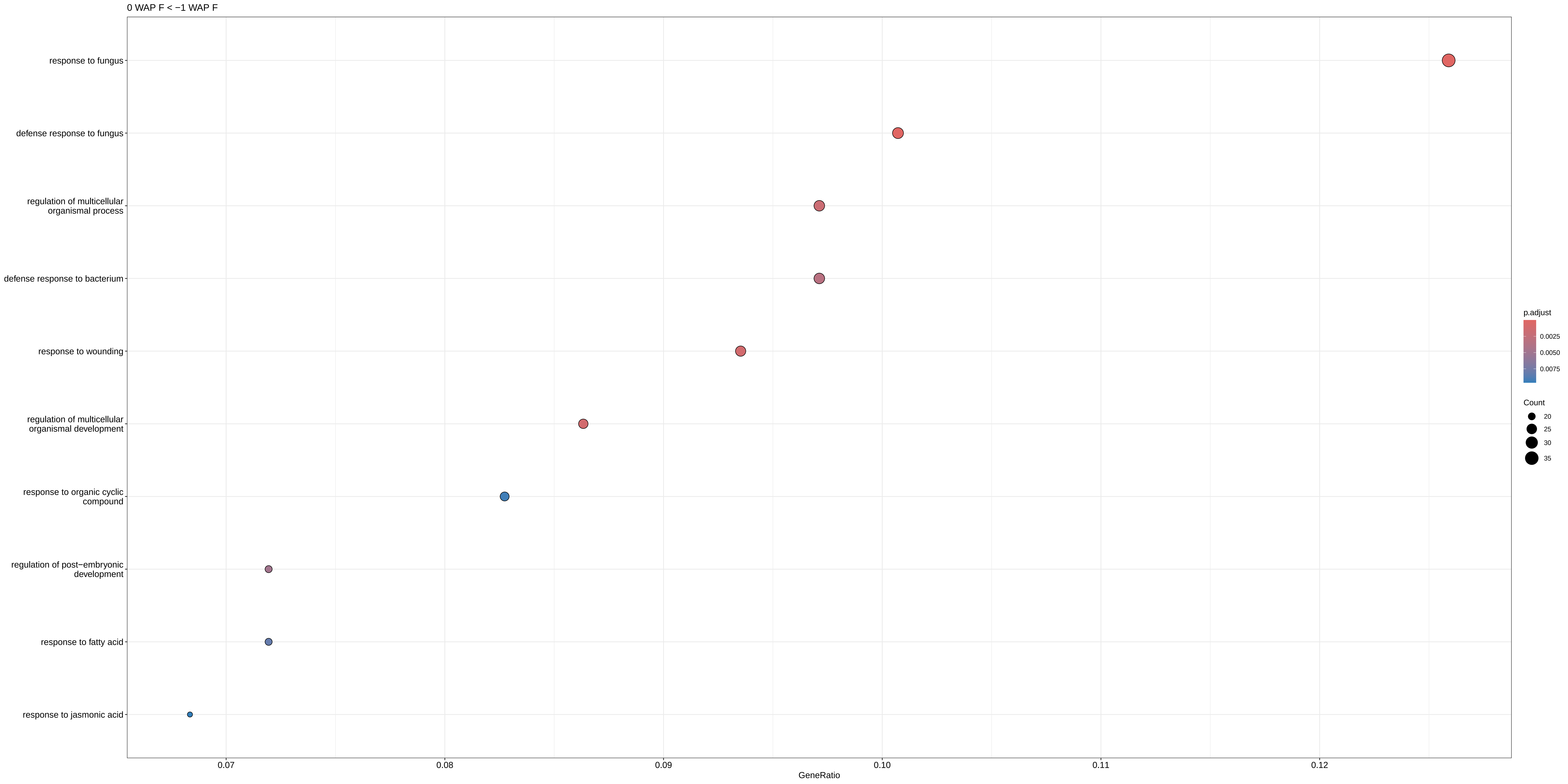
0 WAP F < −1 WAP F
response to fungus
defense response to fungus
regulation of multicellular organismal process
defense response to bacterium
response to wounding
regulation of multicellular organismal development
response to organic cyclic compound
regulation of post−embryonic development
response to fatty acid
response to jasmonic acid
0.07
0.08
0.09
0.10
0.11
0.12
GeneRatio
p.adjust
0.0025
0.0050
0.0075
Count
20
25
30
35

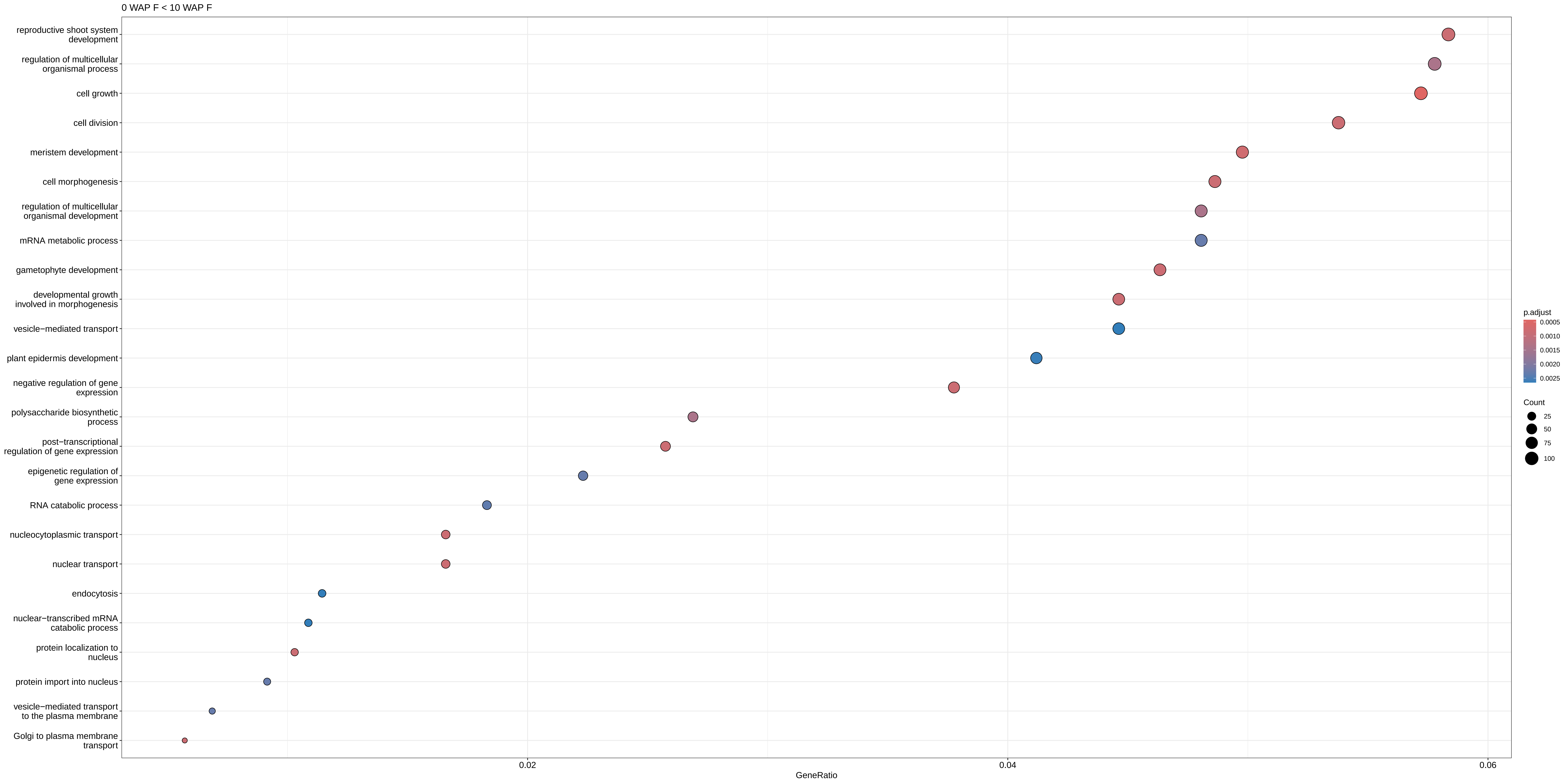

0 WAP F < 10 WAP F
reproductive shoot system development
regulation of multicellular organismal process
cell growth
cell division
meristem development
cell morphogenesis
regulation of multicellular organismal development
mRNA metabolic process
gametophyte development
developmental growth involved in morphogenesis
vesicle−mediated transport
plant epidermis development
negative regulation of gene expression
polysaccharide biosynthetic process
post−transcriptional regulation of gene expression
epigenetic regulation of gene expression
RNA catabolic process
nucleocytoplasmic transport
nuclear transport
endocytosis
nuclear−transcribed mRNA catabolic process
protein localization to nucleus
protein import into nucleus
vesicle−mediated transport to the plasma membrane
Golgi to plasma membrane transport
0.02
0.04
0.06
GeneRatio
p.adjust
0.0005
0.0010
0.0015
0.0020
0.0025
Count
25
50
75
100

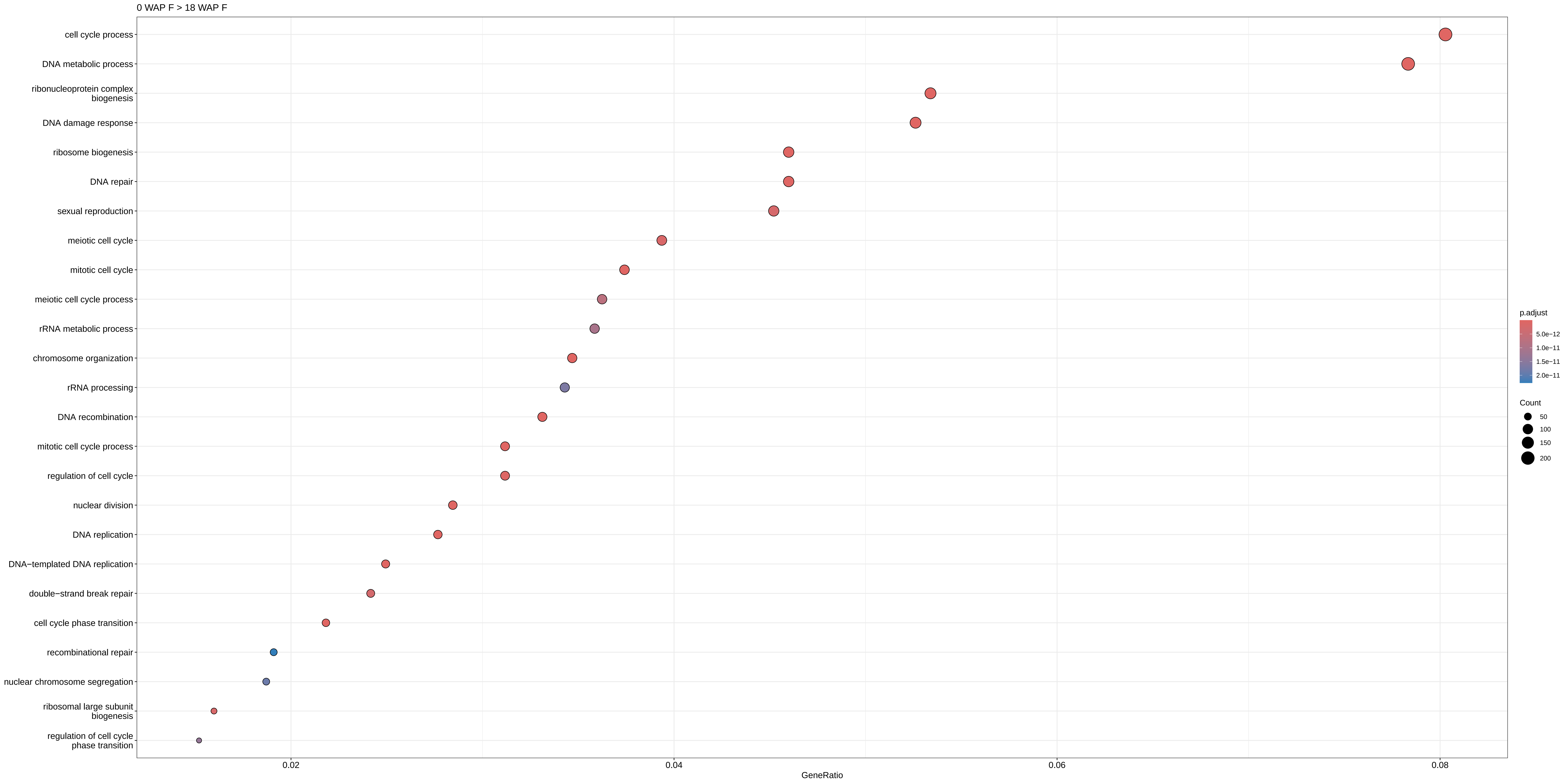

0 WAP F > 18 WAP F
cell cycle process
DNA metabolic process
ribonucleoprotein complex biogenesis
DNA damage response
ribosome biogenesis
DNA repair
sexual reproduction
meiotic cell cycle
mitotic cell cycle
meiotic cell cycle process
rRNA metabolic process
chromosome organization
rRNA processing
DNA recombination
mitotic cell cycle process
regulation of cell cycle
nuclear division
DNA replication
DNA−templated DNA replication
double−strand break repair
cell cycle phase transition
recombinational repair
nuclear chromosome segregation
ribosomal large subunit biogenesis
regulation of cell cycle phase transition
0.02
0.04
0.06
0.08
GeneRatio
p.adjust
5.0e−12
1.0e−11
1.5e−11
2.0e−11
Count
50
100
150
200

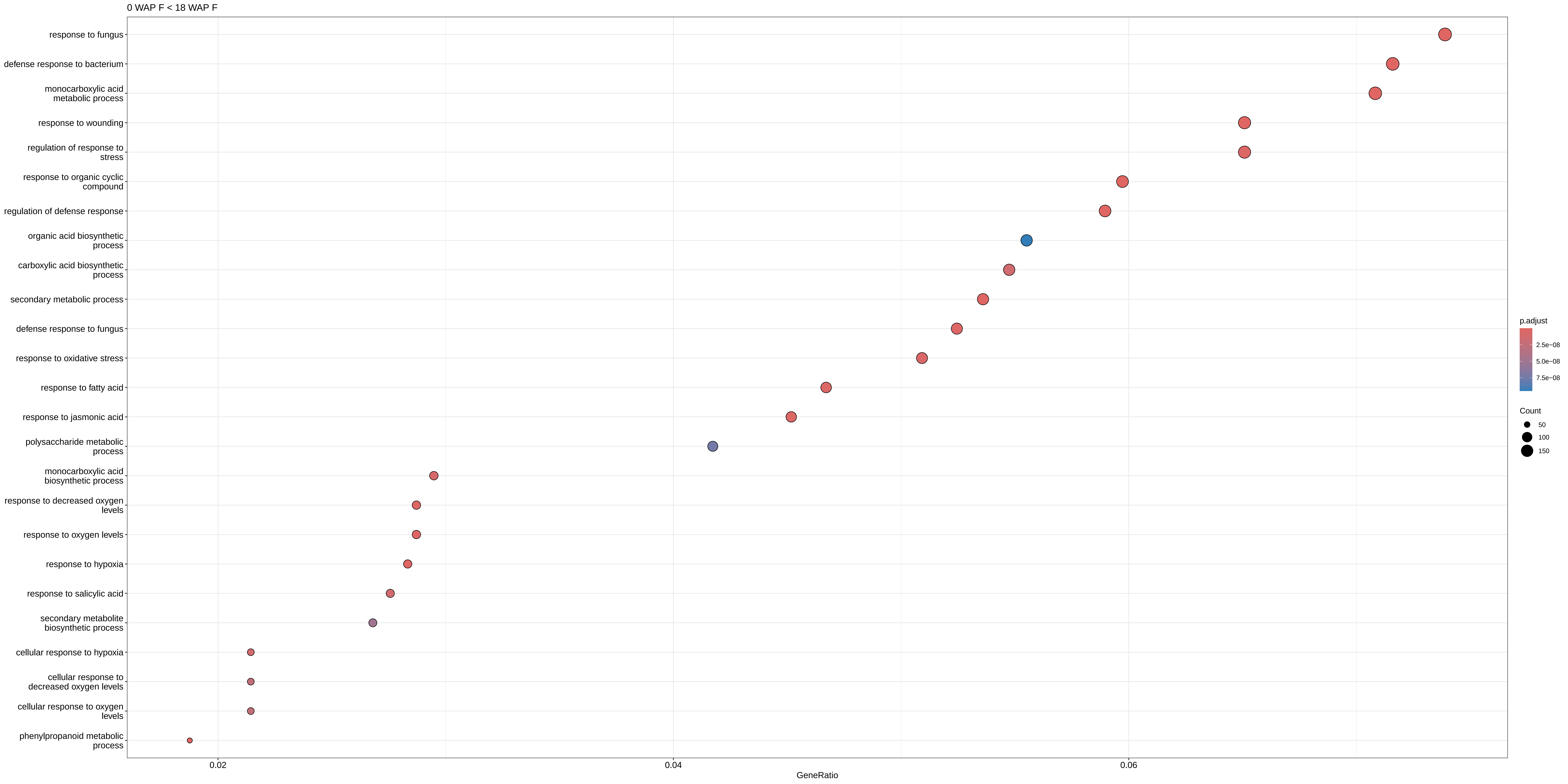
0 WAP F < 18 WAP F
response to fungus
defense response to bacterium
monocarboxylic acid metabolic process
response to wounding
regulation of response to stress
response to organic cyclic compound
regulation of defense response
organic acid biosynthetic process
carboxylic acid biosynthetic process
secondary metabolic process
defense response to fungus
response to oxidative stress
response to fatty acid
response to jasmonic acid
polysaccharide metabolic process
monocarboxylic acid biosynthetic process
response to decreased oxygen levels
response to oxygen levels
response to hypoxia
response to salicylic acid
secondary metabolite biosynthetic process
cellular response to hypoxia
cellular response to decreased oxygen levels
cellular response to oxygen levels
phenylpropanoid metabolic process
0.02
0.04
0.06
GeneRatio
p.adjust
2.5e−08
5.0e−08
7.5e−08
Count
50
100
150

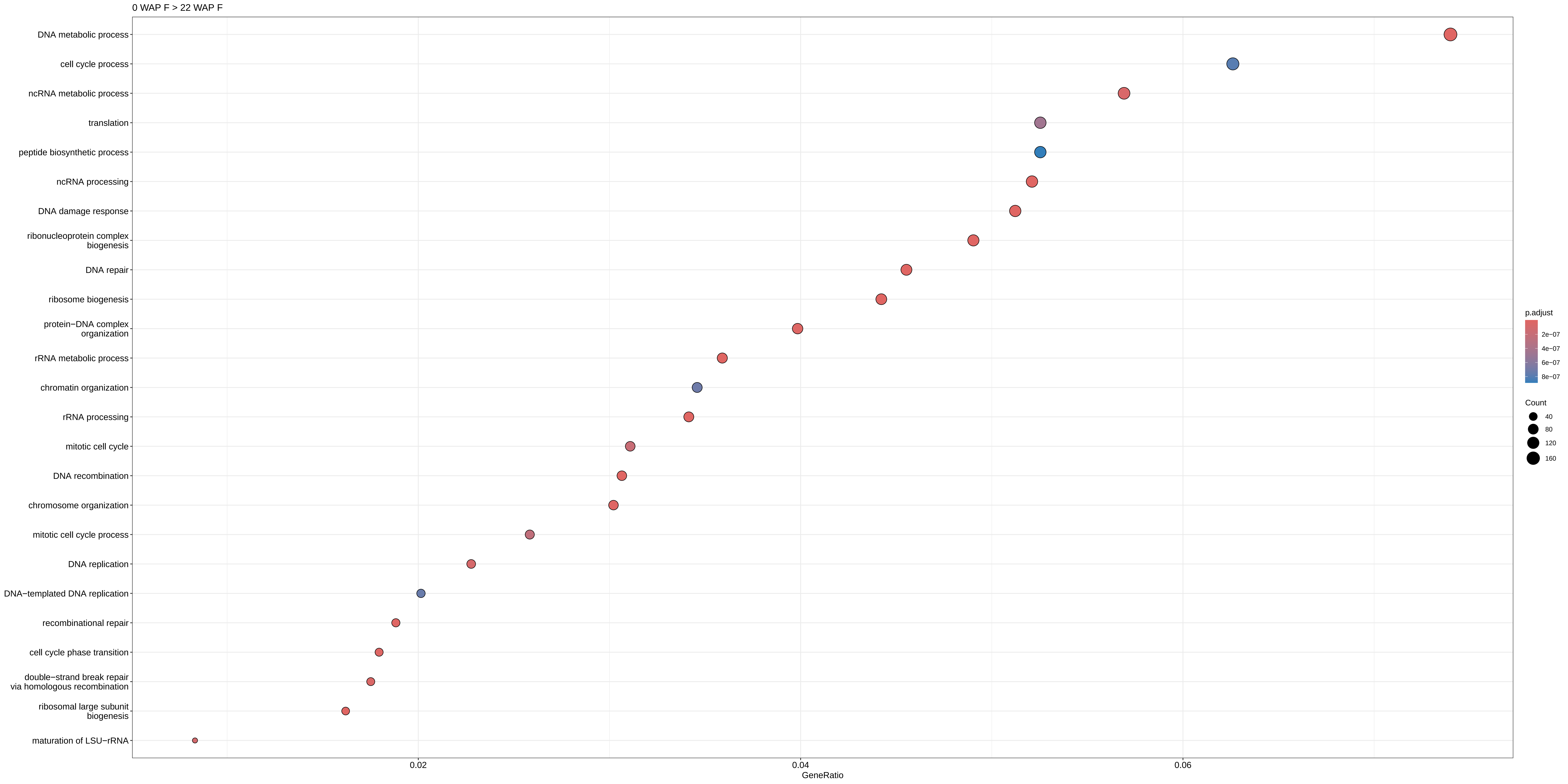
0 WAP F > 22 WAP F
DNA metabolic process
cell cycle process
ncRNA metabolic process
translation
peptide biosynthetic process
ncRNA processing
DNA damage response
ribonucleoprotein complex biogenesis
DNA repair
ribosome biogenesis
protein−DNA complex organization
rRNA metabolic process
chromatin organization
rRNA processing
mitotic cell cycle
DNA recombination
chromosome organization
mitotic cell cycle process
DNA replication
DNA−templated DNA replication
recombinational repair
cell cycle phase transition
double−strand break repair via homologous recombination
ribosomal large subunit biogenesis
maturation of LSU−rRNA
0.02
0.04
0.06
GeneRatio
p.adjust
2e−07
4e−07
6e−07
8e−07
Count
40
80
120
160

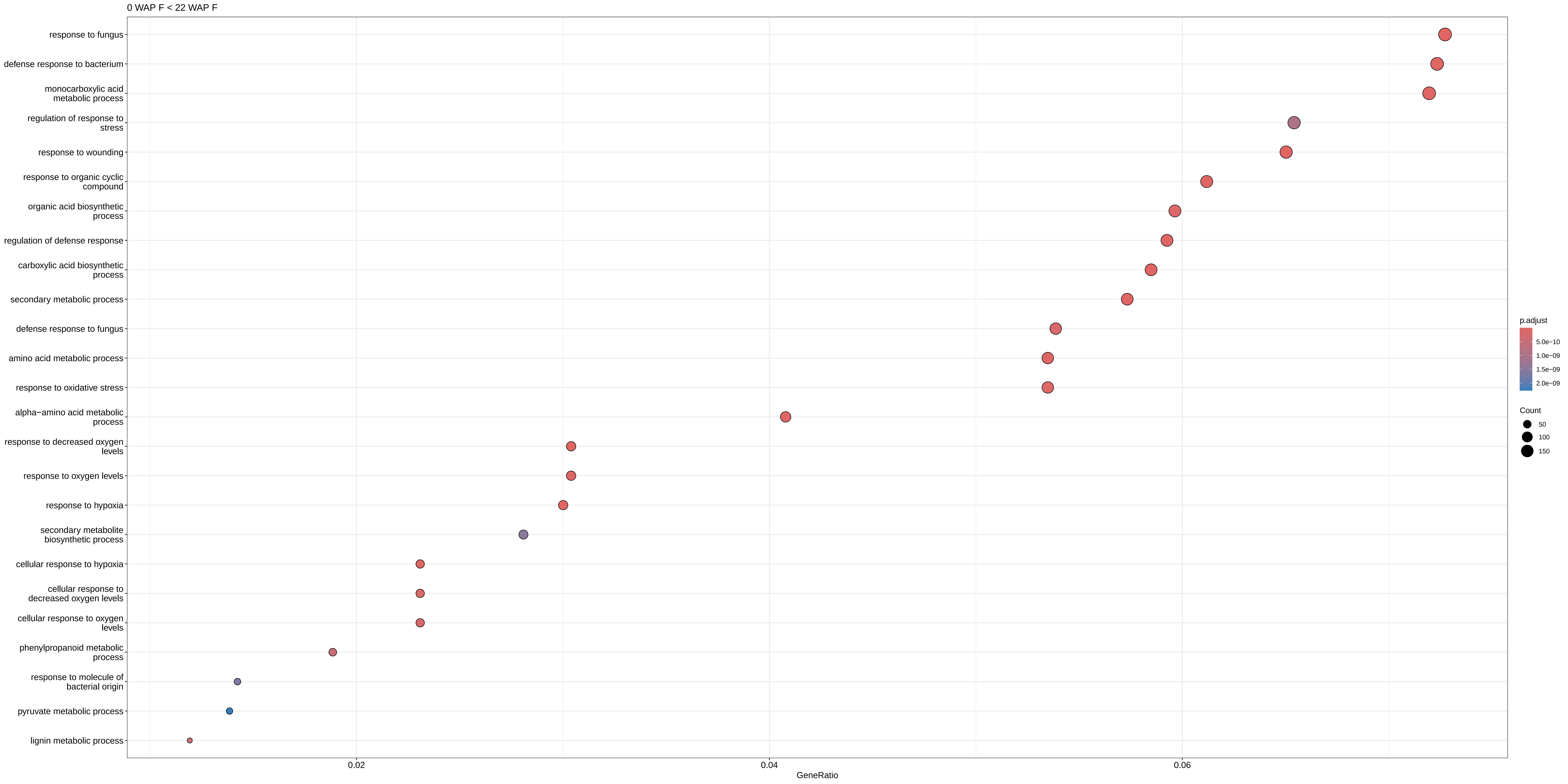

0 WAP F < 22 WAP F
response to fungus
defense response to bacterium
monocarboxylic acid metabolic process
regulation of response to stress
response to wounding
response to organic cyclic compound
organic acid biosynthetic process
regulation of defense response
carboxylic acid biosynthetic process
secondary metabolic process
defense response to fungus
amino acid metabolic process
response to oxidative stress
alpha−amino acid metabolic process
response to decreased oxygen levels
response to oxygen levels
response to hypoxia
secondary metabolite biosynthetic process
cellular response to hypoxia
cellular response to decreased oxygen levels
cellular response to oxygen levels
phenylpropanoid metabolic process
response to molecule of bacterial origin
pyruvate metabolic process
lignin metabolic process
0.02
0.04
0.06
GeneRatio
p.adjust
5.0e−10
1.0e−09
1.5e−09
2.0e−09
Count
50
100
150

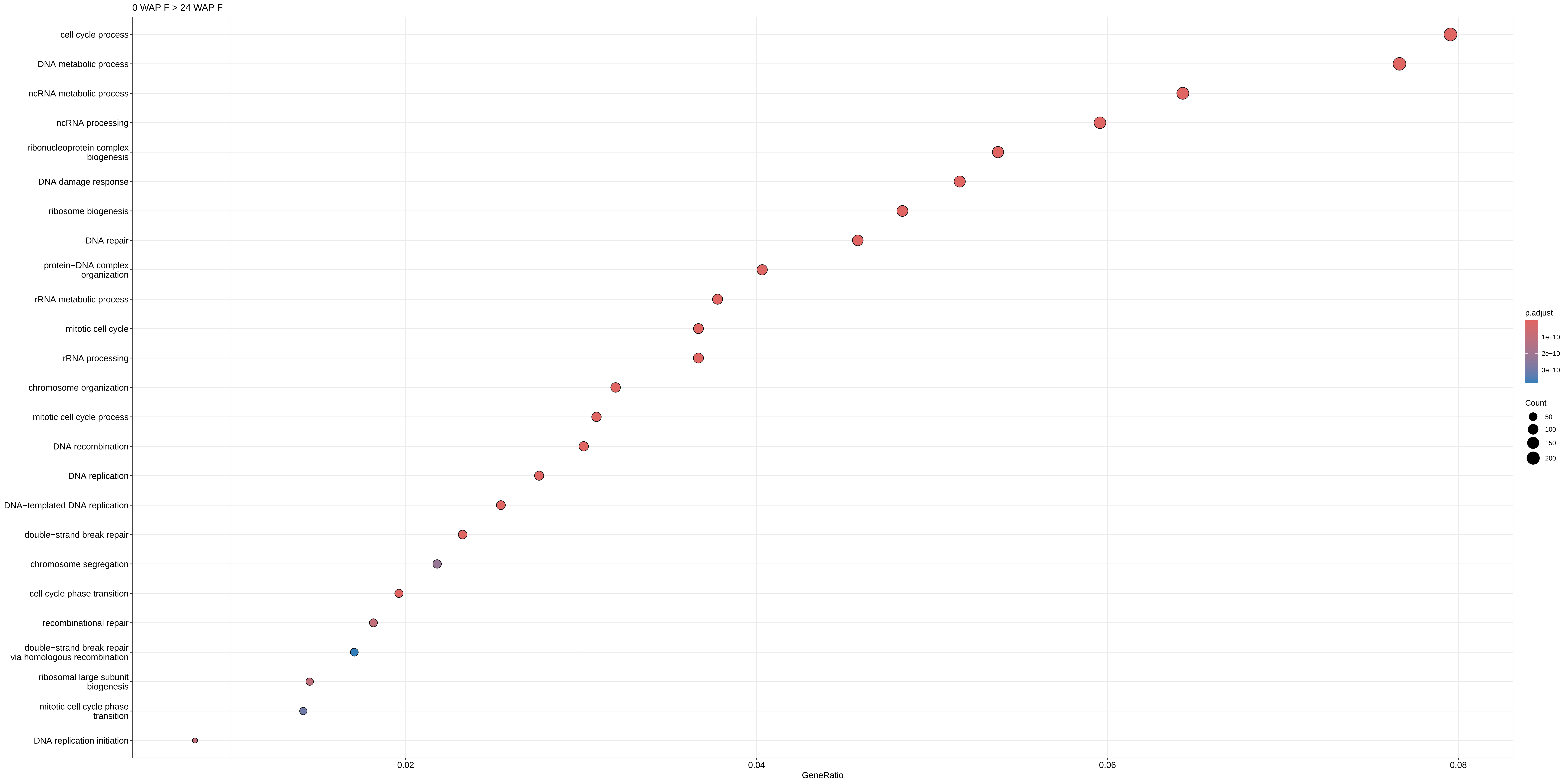
0 WAP F > 24 WAP F
cell cycle process
DNA metabolic process
ncRNA metabolic process
ncRNA processing
ribonucleoprotein complex biogenesis
DNA damage response
ribosome biogenesis
DNA repair
protein−DNA complex organization
rRNA metabolic process
mitotic cell cycle
rRNA processing
chromosome organization
mitotic cell cycle process
DNA recombination
DNA replication
DNA−templated DNA replication
double−strand break repair
chromosome segregation
cell cycle phase transition
recombinational repair
double−strand break repair via homologous recombination
ribosomal large subunit biogenesis
mitotic cell cycle phase transition
DNA replication initiation
0.02
0.04
0.06
0.08
GeneRatio
p.adjust
1e−10
2e−10
3e−10
Count
50
100
150
200

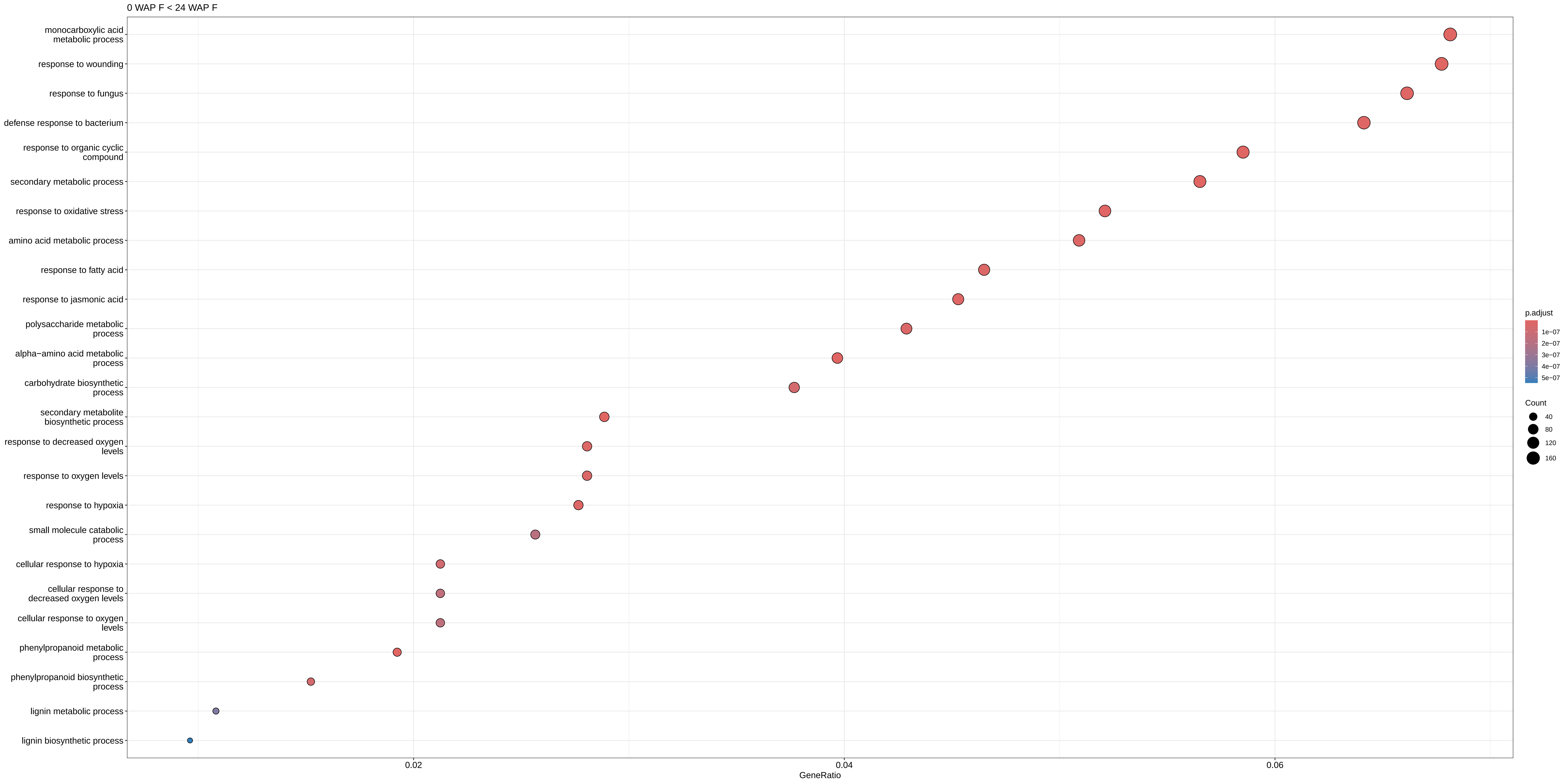
0 WAP F < 24 WAP F
monocarboxylic acid metabolic process
response to wounding
response to fungus
defense response to bacterium
response to organic cyclic compound
secondary metabolic process
response to oxidative stress
amino acid metabolic process
response to fatty acid
response to jasmonic acid
polysaccharide metabolic process
alpha−amino acid metabolic process
carbohydrate biosynthetic process
secondary metabolite biosynthetic process
response to decreased oxygen levels
response to oxygen levels
response to hypoxia
small molecule catabolic process
cellular response to hypoxia
cellular response to decreased oxygen levels
cellular response to oxygen levels
phenylpropanoid metabolic process
phenylpropanoid biosynthetic process
lignin metabolic process
lignin biosynthetic process
0.02
0.04
0.06
GeneRatio
p.adjust
1e−07
2e−07
3e−07
4e−07
5e−07
Count
40
80
120
160

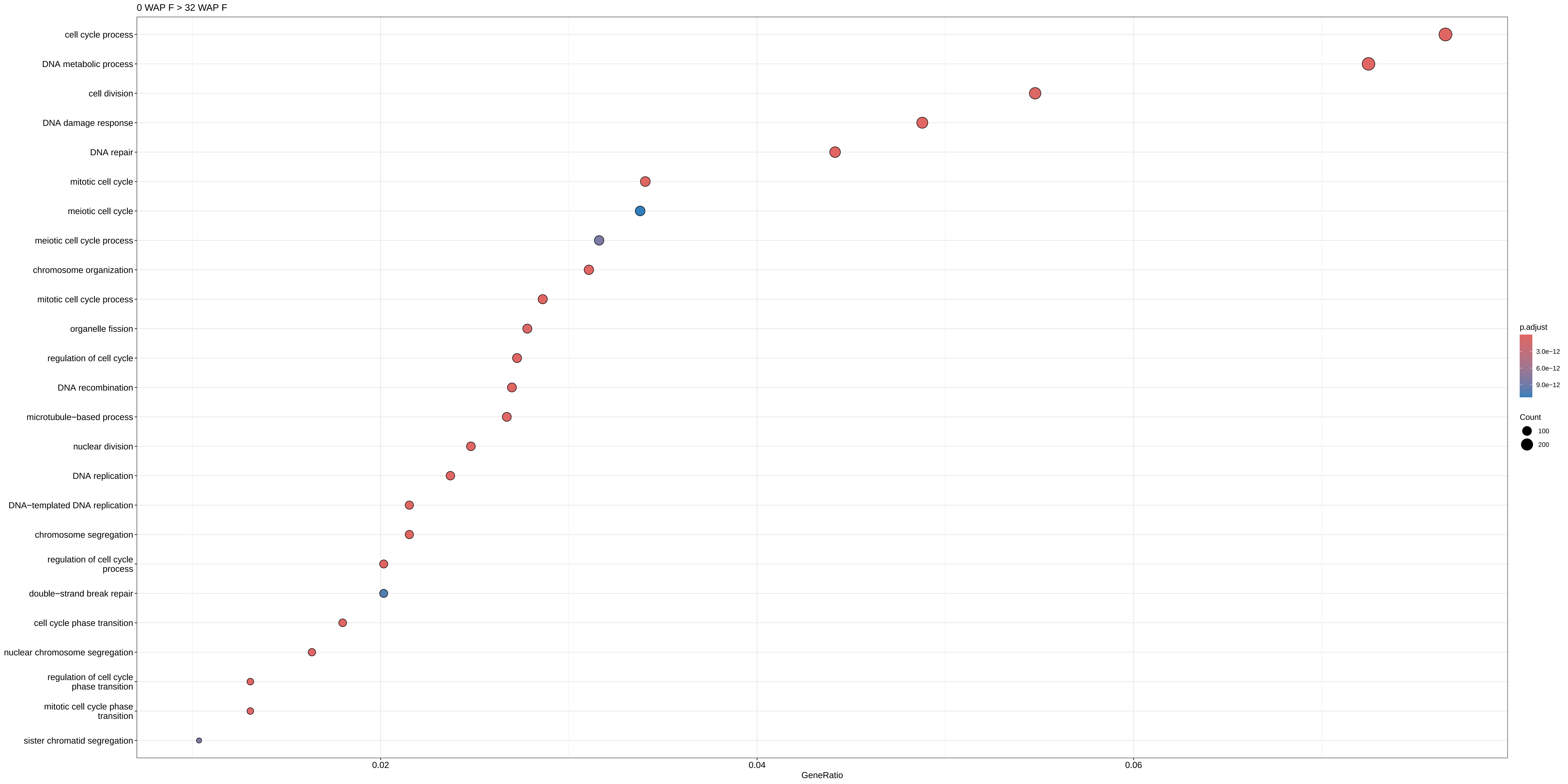

0 WAP F > 32 WAP F
cell cycle process
DNA metabolic process
cell division
DNA damage response
DNA repair
mitotic cell cycle
meiotic cell cycle
meiotic cell cycle process
chromosome organization
mitotic cell cycle process
organelle fission
regulation of cell cycle
DNA recombination
microtubule−based process
nuclear division
DNA replication
DNA−templated DNA replication
chromosome segregation
regulation of cell cycle process
double−strand break repair
cell cycle phase transition
nuclear chromosome segregation
regulation of cell cycle phase transition
mitotic cell cycle phase transition
sister chromatid segregation
0.02
0.04
0.06
GeneRatio
p.adjust
3.0e−12
6.0e−12
9.0e−12
Count
100
200

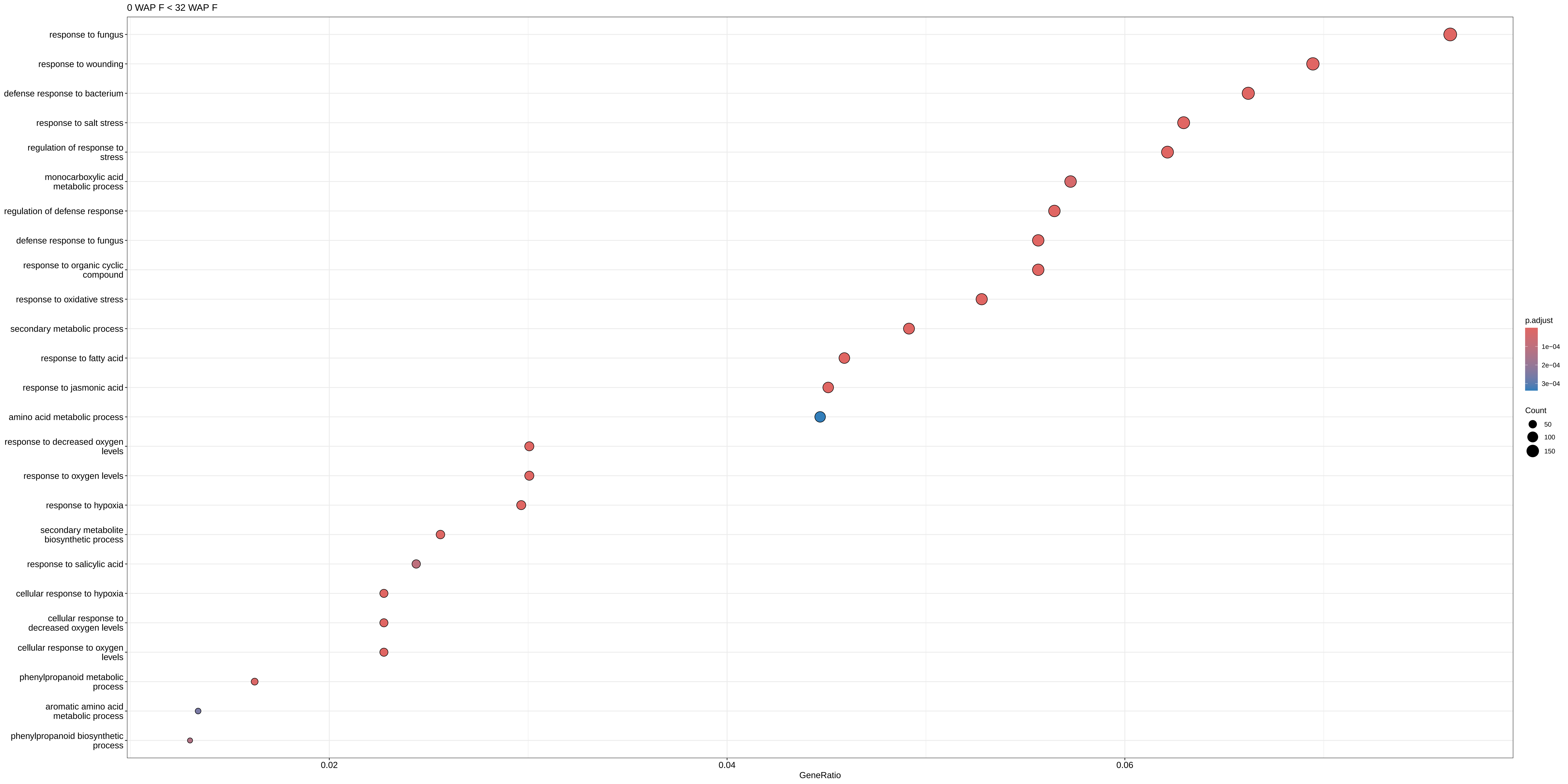
0 WAP F < 32 WAP F
response to fungus
response to wounding
defense response to bacterium
response to salt stress
regulation of response to stress
monocarboxylic acid metabolic process
regulation of defense response
defense response to fungus
response to organic cyclic compound
response to oxidative stress
secondary metabolic process
response to fatty acid
response to jasmonic acid
amino acid metabolic process
response to decreased oxygen levels
response to oxygen levels
response to hypoxia
secondary metabolite biosynthetic process
response to salicylic acid
cellular response to hypoxia
cellular response to decreased oxygen levels
cellular response to oxygen levels
phenylpropanoid metabolic process
aromatic amino acid metabolic process
phenylpropanoid biosynthetic process
0.02
0.04
0.06
GeneRatio
p.adjust
1e−04
2e−04
3e−04
Count
50
100
150

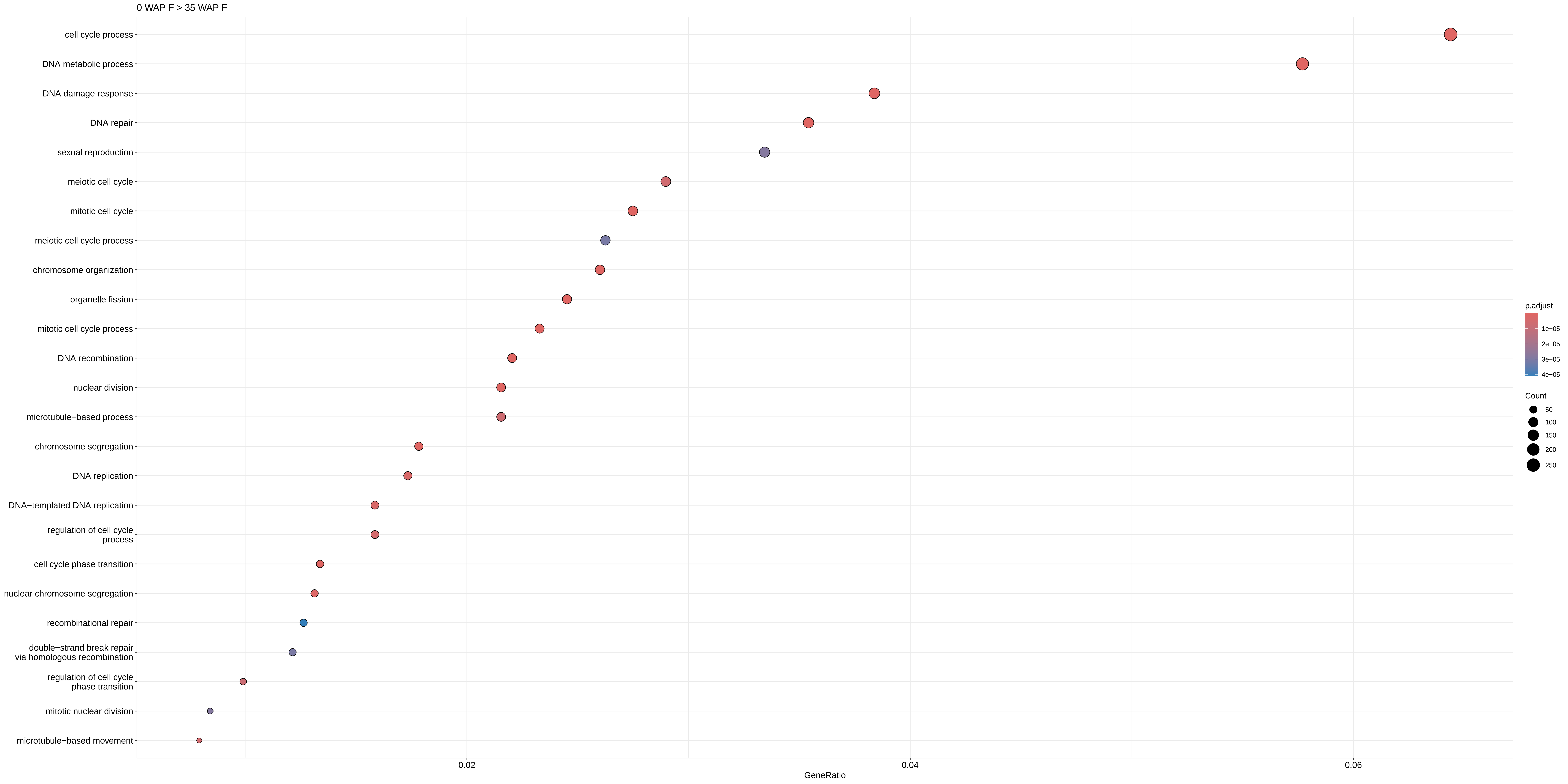
0 WAP F > 35 WAP F
cell cycle process
DNA metabolic process
DNA damage response
DNA repair
sexual reproduction
meiotic cell cycle
mitotic cell cycle
meiotic cell cycle process
chromosome organization
organelle fission
mitotic cell cycle process
DNA recombination
nuclear division
microtubule−based process
chromosome segregation
DNA replication
DNA−templated DNA replication
regulation of cell cycle process
cell cycle phase transition
nuclear chromosome segregation
recombinational repair
double−strand break repair via homologous recombination
regulation of cell cycle phase transition
mitotic nuclear division
microtubule−based movement
0.02
0.04
0.06
GeneRatio
p.adjust
1e−05
2e−05
3e−05
4e−05
Count
50
100
150
200
250

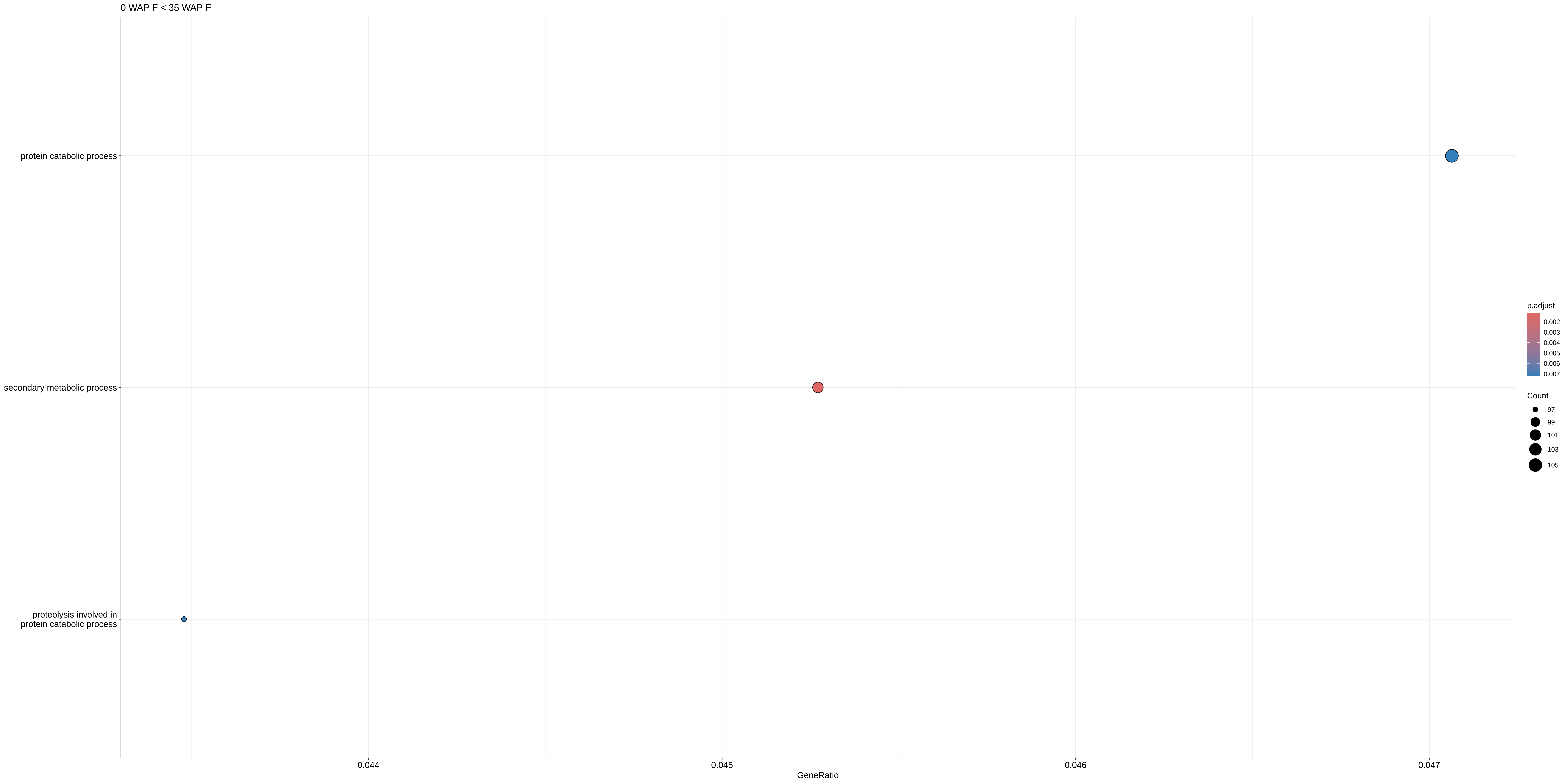
0 WAP F < 35 WAP F
protein catabolic process
secondary metabolic process
proteolysis involved in protein catabolic process
0.044
0.045
0.046
0.047
GeneRatio
p.adjust
0.002
0.003
0.004
0.005
0.006
0.007
Count
97
99
101
103
105

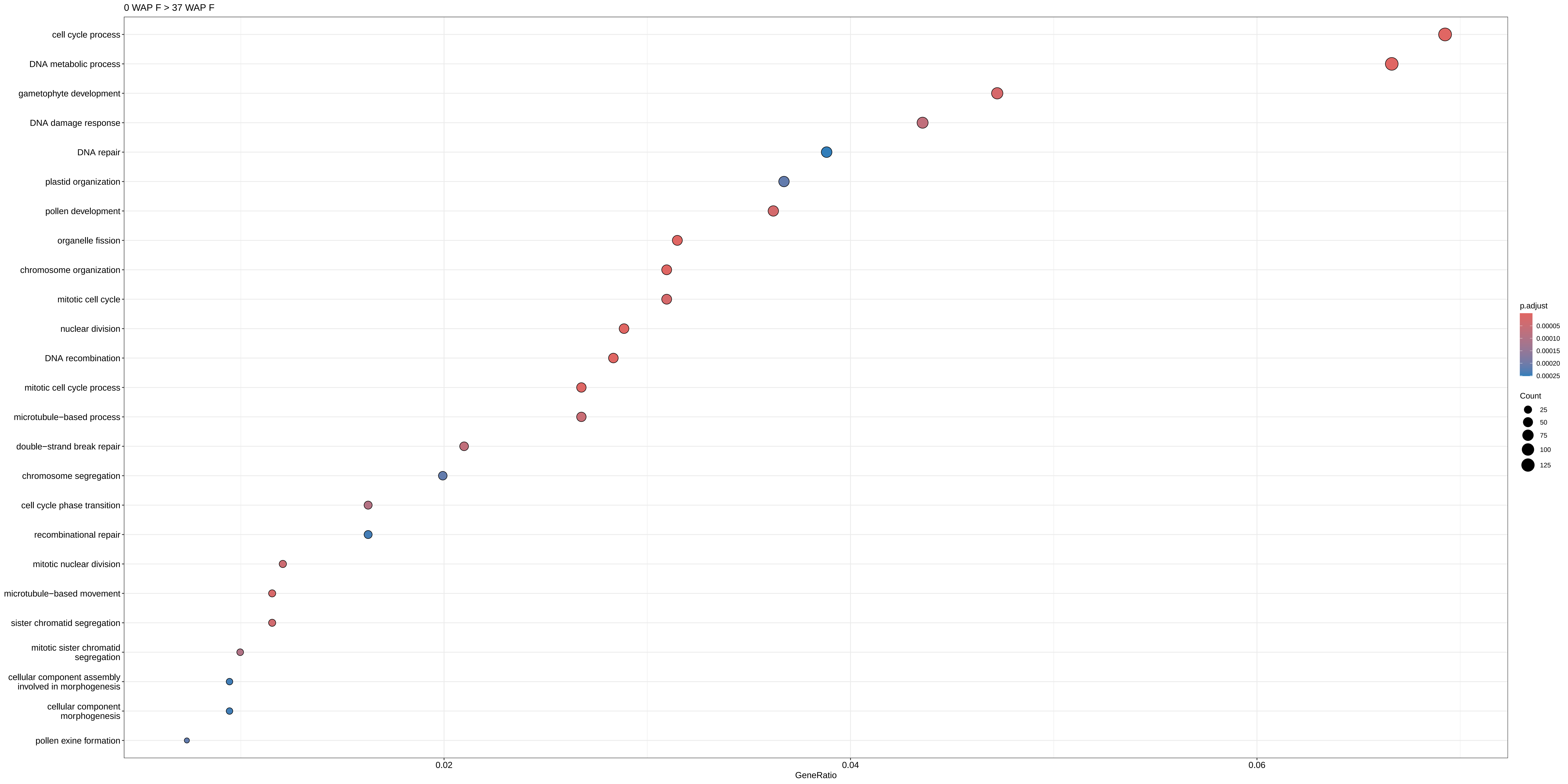
0 WAP F > 37 WAP F
cell cycle process
DNA metabolic process
gametophyte development
DNA damage response
DNA repair
plastid organization
pollen development
organelle fission
chromosome organization
mitotic cell cycle
nuclear division
DNA recombination
mitotic cell cycle process
microtubule−based process
double−strand break repair
chromosome segregation
cell cycle phase transition
recombinational repair
mitotic nuclear division
microtubule−based movement
sister chromatid segregation
mitotic sister chromatid segregation
cellular component assembly involved in morphogenesis
cellular component morphogenesis
pollen exine formation
0.02
0.04
0.06
GeneRatio
p.adjust
0.00005
0.00010
0.00015
0.00020
0.00025
Count
25
50
75
100
125

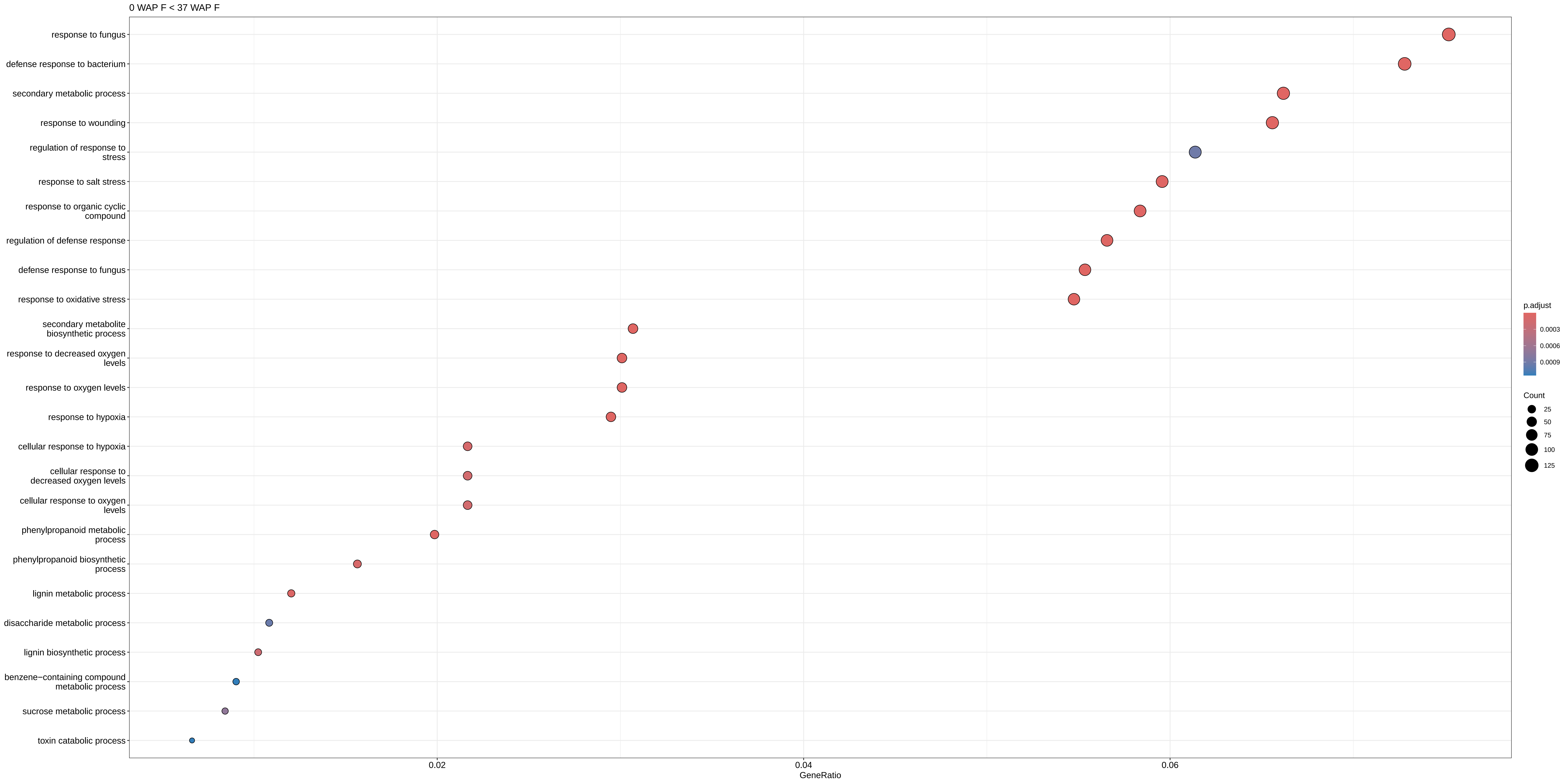

0 WAP F < 37 WAP F
response to fungus
defense response to bacterium
secondary metabolic process
response to wounding
regulation of response to stress
response to salt stress
response to organic cyclic compound
regulation of defense response
defense response to fungus
response to oxidative stress
secondary metabolite biosynthetic process
response to decreased oxygen levels
response to oxygen levels
response to hypoxia
cellular response to hypoxia
cellular response to decreased oxygen levels
cellular response to oxygen levels
phenylpropanoid metabolic process
phenylpropanoid biosynthetic process
lignin metabolic process
disaccharide metabolic process
lignin biosynthetic process
benzene−containing compound metabolic process
sucrose metabolic process
toxin catabolic process
0.02
0.04
0.06
GeneRatio
p.adjust
0.0003
0.0006
0.0009
Count
25
50
75
100
125

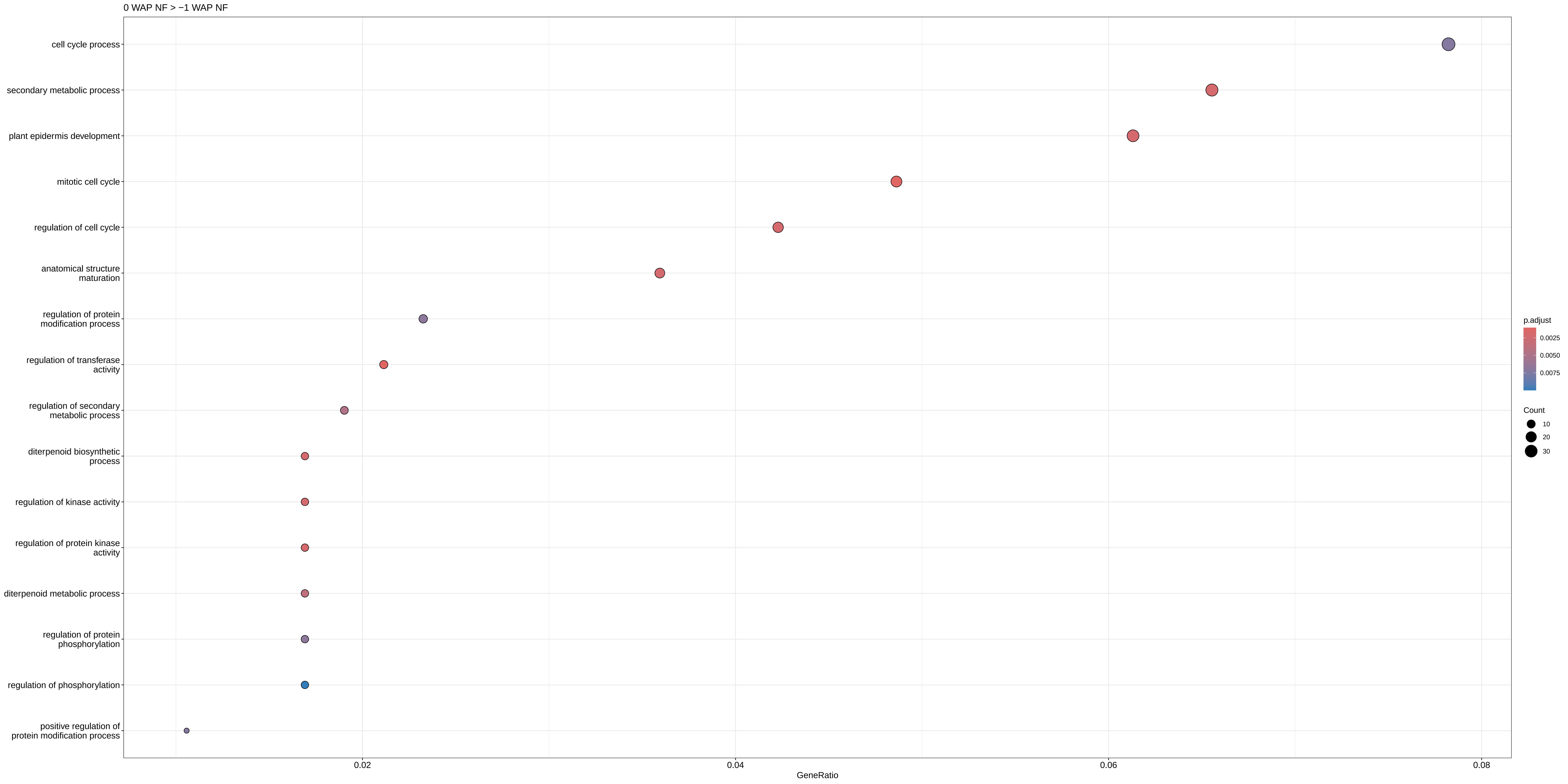

0 WAP NF > −1 WAP NF
cell cycle process
secondary metabolic process
plant epidermis development
mitotic cell cycle
regulation of cell cycle
anatomical structure maturation
regulation of protein modification process
regulation of transferase activity
regulation of secondary metabolic process
diterpenoid biosynthetic process
regulation of kinase activity
regulation of protein kinase activity
diterpenoid metabolic process
regulation of protein phosphorylation
regulation of phosphorylation
positive regulation of protein modification process
0.02
0.04
0.06
0.08
GeneRatio
p.adjust
0.0025
0.0050
0.0075
Count
10
20
30

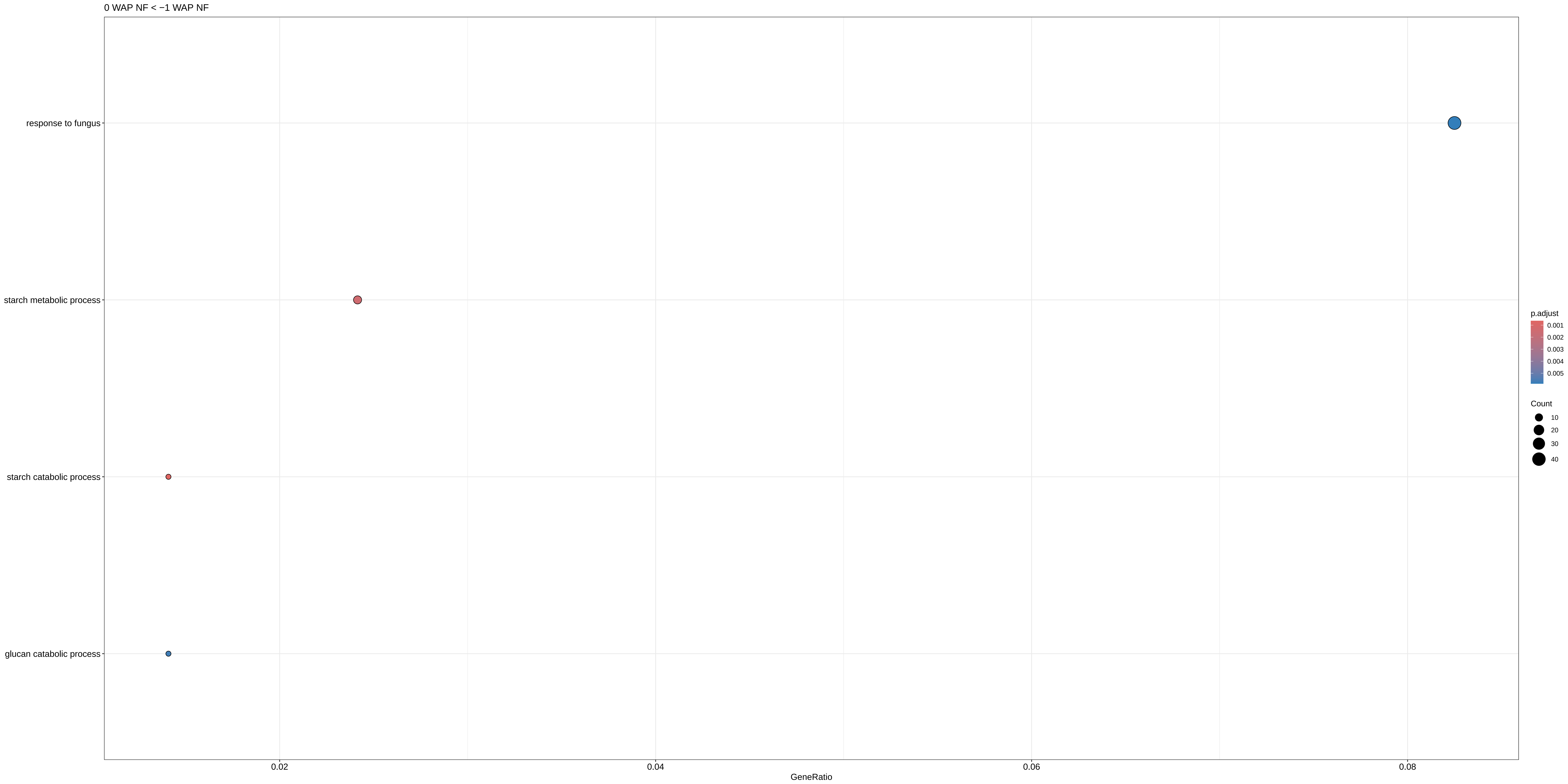

0 WAP NF < −1 WAP NF
response to fungus
starch metabolic process
starch catabolic process
glucan catabolic process
0.02
0.04
0.06
0.08
GeneRatio
p.adjust
0.001
0.002
0.003
0.004
0.005
Count
10
20
30
40

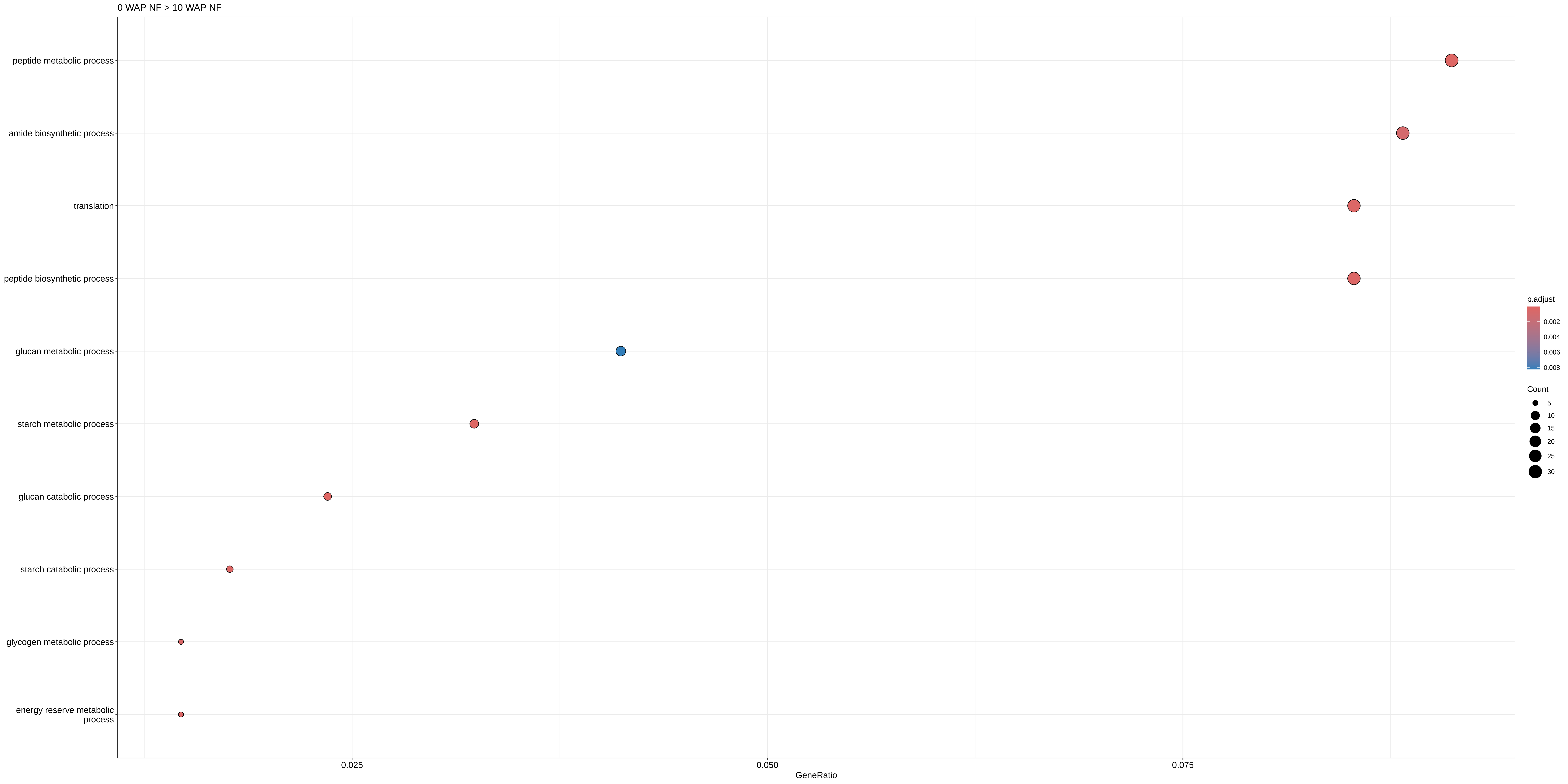
0 WAP NF > 10 WAP NF
peptide metabolic process
amide biosynthetic process
translation
peptide biosynthetic process
glucan metabolic process
starch metabolic process
glucan catabolic process
starch catabolic process
glycogen metabolic process
energy reserve metabolic process
0.025
0.050
0.075
GeneRatio
p.adjust
0.002
0.004
0.006
0.008
Count
5
10
15
20
25
30

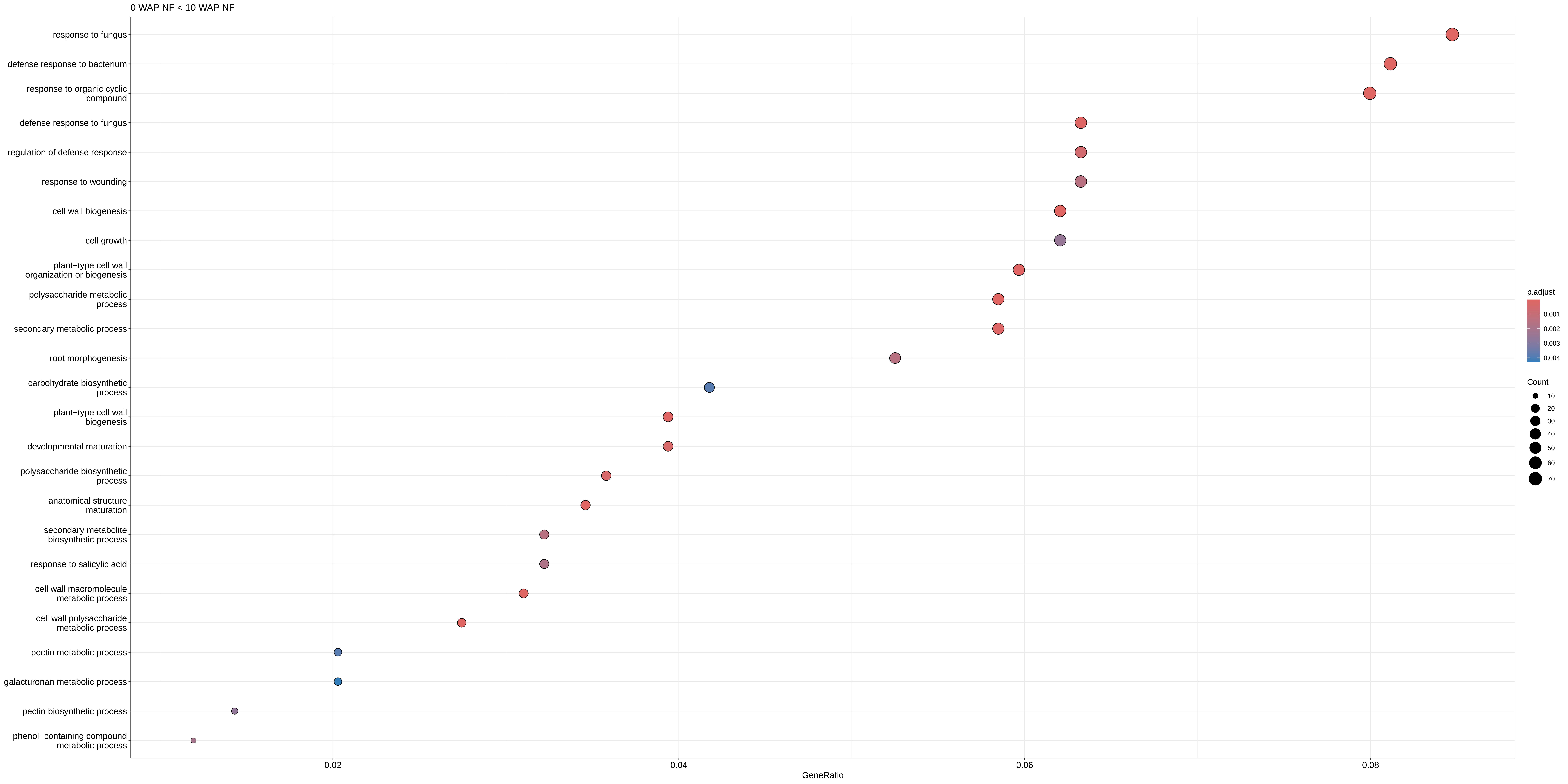

0 WAP NF < 10 WAP NF
response to fungus
defense response to bacterium
response to organic cyclic compound
defense response to fungus
regulation of defense response
response to wounding
cell wall biogenesis
cell growth
plant−type cell wall organization or biogenesis
polysaccharide metabolic process
secondary metabolic process
root morphogenesis
carbohydrate biosynthetic process
plant−type cell wall biogenesis
developmental maturation
polysaccharide biosynthetic process
anatomical structure maturation
secondary metabolite biosynthetic process
response to salicylic acid
cell wall macromolecule metabolic process
cell wall polysaccharide metabolic process
pectin metabolic process
galacturonan metabolic process
pectin biosynthetic process
phenol−containing compound metabolic process
0.02
0.04
0.06
0.08
GeneRatio
p.adjust
0.001
0.002
0.003
0.004
Count
10
20
30
40
50
60
70

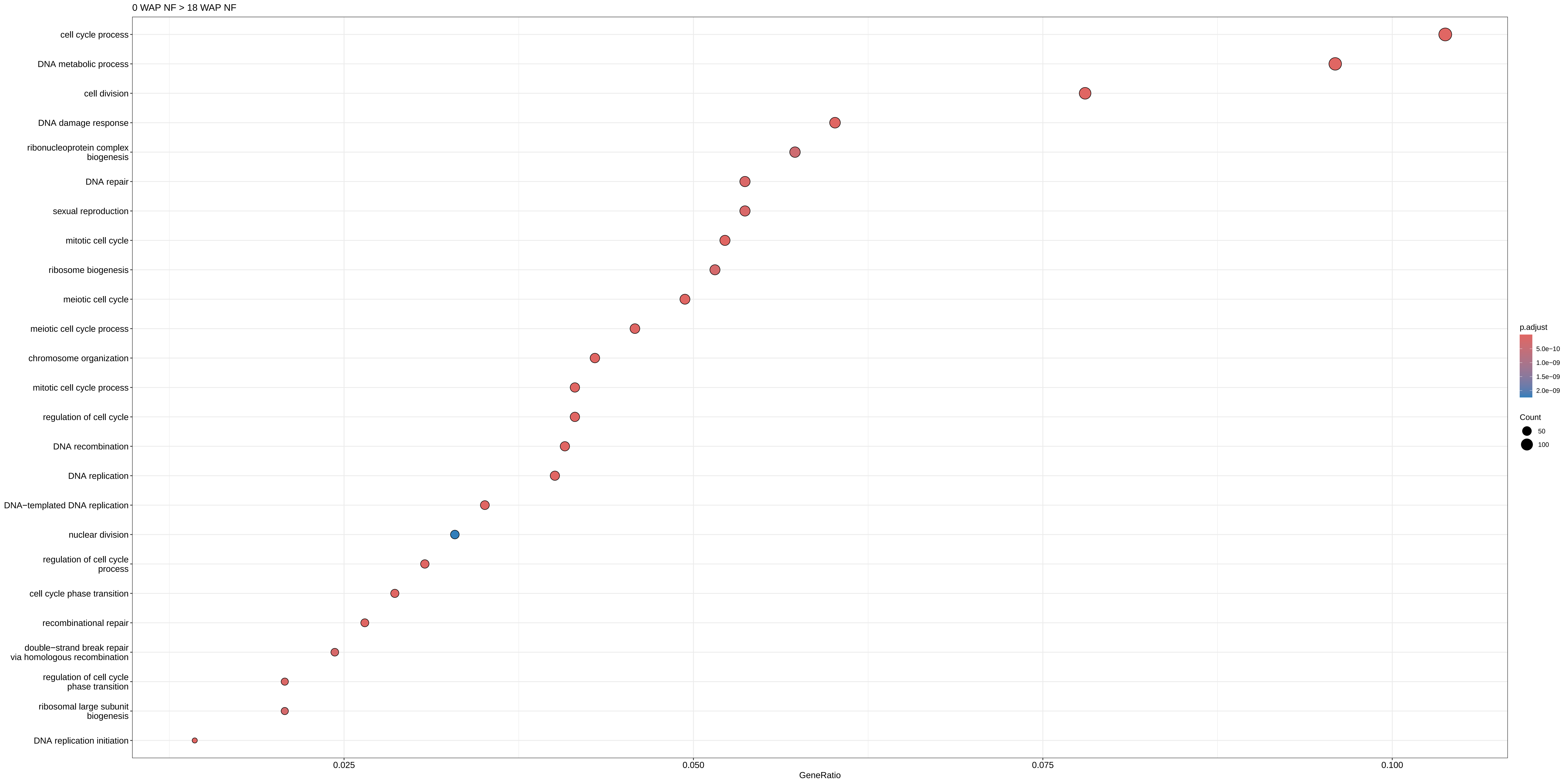

0 WAP NF > 18 WAP NF
cell cycle process
DNA metabolic process
cell division
DNA damage response
ribonucleoprotein complex biogenesis
DNA repair
sexual reproduction
mitotic cell cycle
ribosome biogenesis
meiotic cell cycle
meiotic cell cycle process
chromosome organization
mitotic cell cycle process
regulation of cell cycle
DNA recombination
DNA replication
DNA−templated DNA replication
nuclear division
regulation of cell cycle process
cell cycle phase transition
recombinational repair
double−strand break repair via homologous recombination
regulation of cell cycle phase transition
ribosomal large subunit biogenesis
DNA replication initiation
0.025
0.050
0.075
0.100
GeneRatio
p.adjust
5.0e−10
1.0e−09
1.5e−09
2.0e−09
Count
50
100

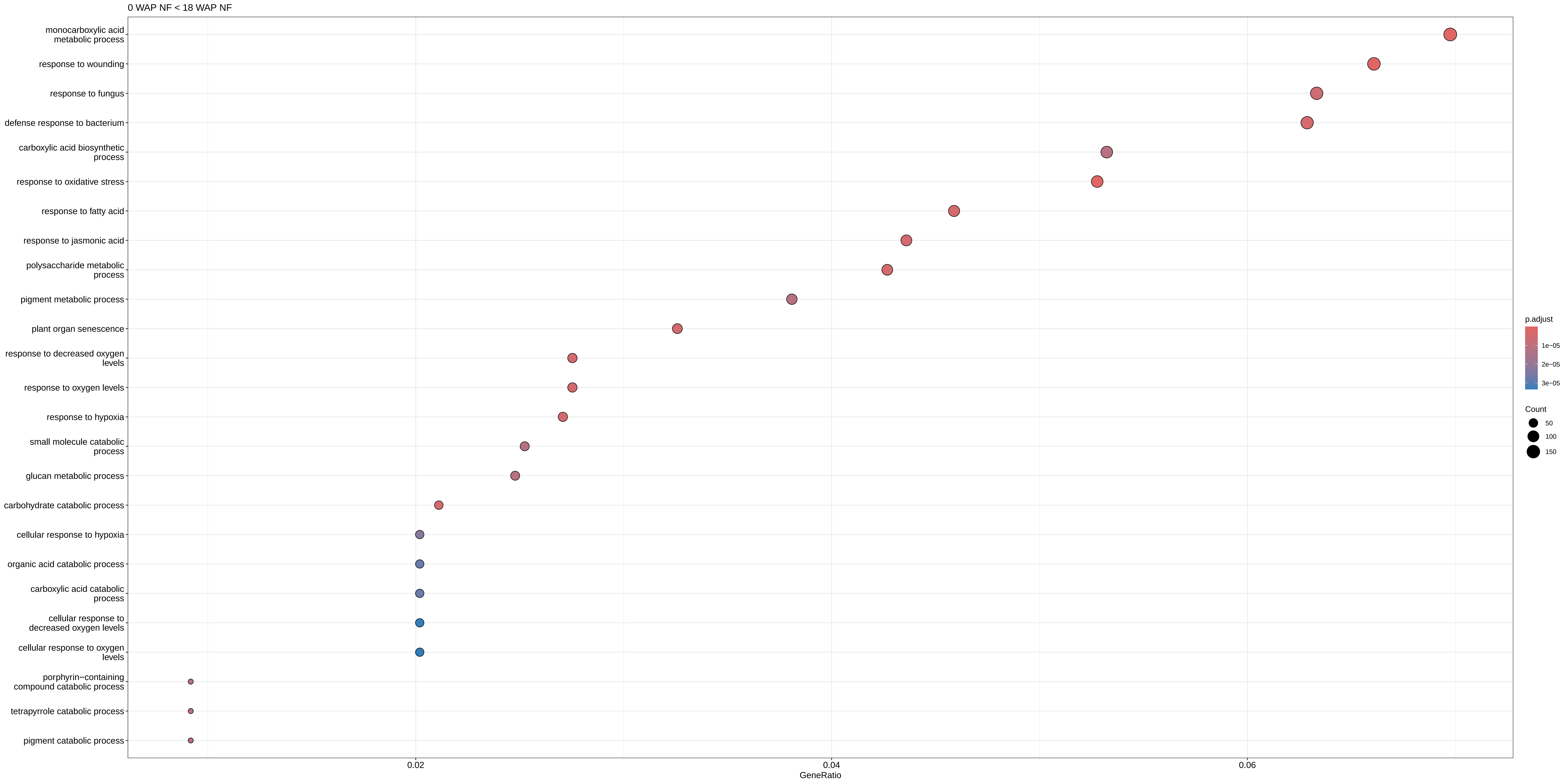
0 WAP NF < 18 WAP NF
monocarboxylic acid metabolic process
response to wounding
response to fungus
defense response to bacterium
carboxylic acid biosynthetic process
response to oxidative stress
response to fatty acid
response to jasmonic acid
polysaccharide metabolic process
pigment metabolic process
plant organ senescence
response to decreased oxygen levels
response to oxygen levels
response to hypoxia
small molecule catabolic process
glucan metabolic process
carbohydrate catabolic process
cellular response to hypoxia
organic acid catabolic process
carboxylic acid catabolic process
cellular response to decreased oxygen levels
cellular response to oxygen levels
porphyrin−containing compound catabolic process
tetrapyrrole catabolic process
pigment catabolic process
0.02
0.04
0.06
GeneRatio
p.adjust
1e−05
2e−05
3e−05
Count
50
100
150

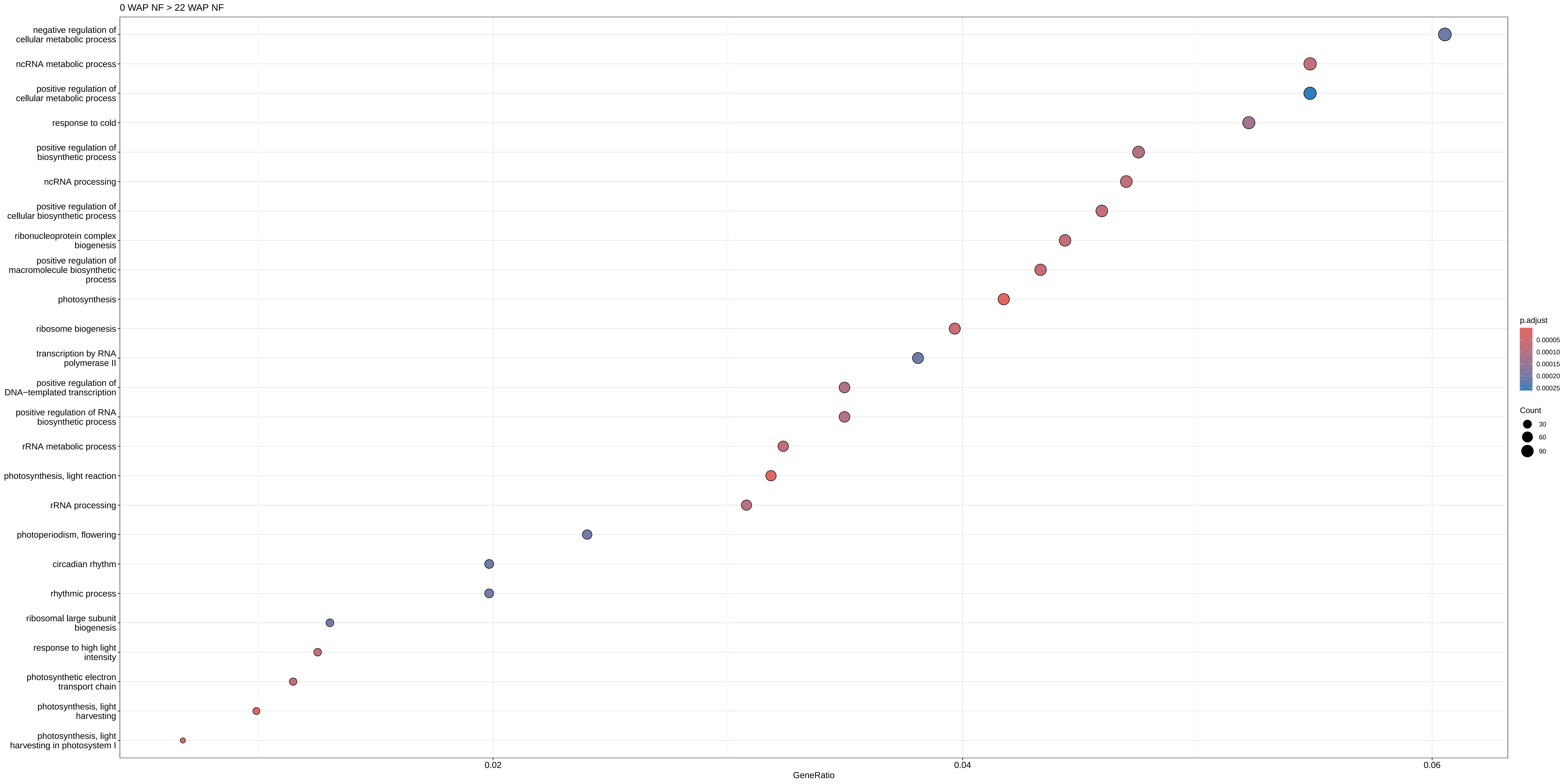

0 WAP NF > 22 WAP NF
negative regulation of cellular metabolic process
ncRNA metabolic process
positive regulation of cellular metabolic process
response to cold
positive regulation of biosynthetic process
ncRNA processing
positive regulation of cellular biosynthetic process
ribonucleoprotein complex biogenesis
positive regulation of macromolecule biosynthetic process
photosynthesis
ribosome biogenesis
transcription by RNA polymerase II
positive regulation of DNA−templated transcription
positive regulation of RNA biosynthetic process
rRNA metabolic process
photosynthesis, light reaction
rRNA processing
photoperiodism, flowering
circadian rhythm
rhythmic process
ribosomal large subunit biogenesis
response to high light intensity
photosynthetic electron transport chain
photosynthesis, light harvesting
photosynthesis, light harvesting in photosystem I
0.02
0.04
0.06
GeneRatio
p.adjust
0.00005
0.00010
0.00015
0.00020
0.00025
Count
30
60
90

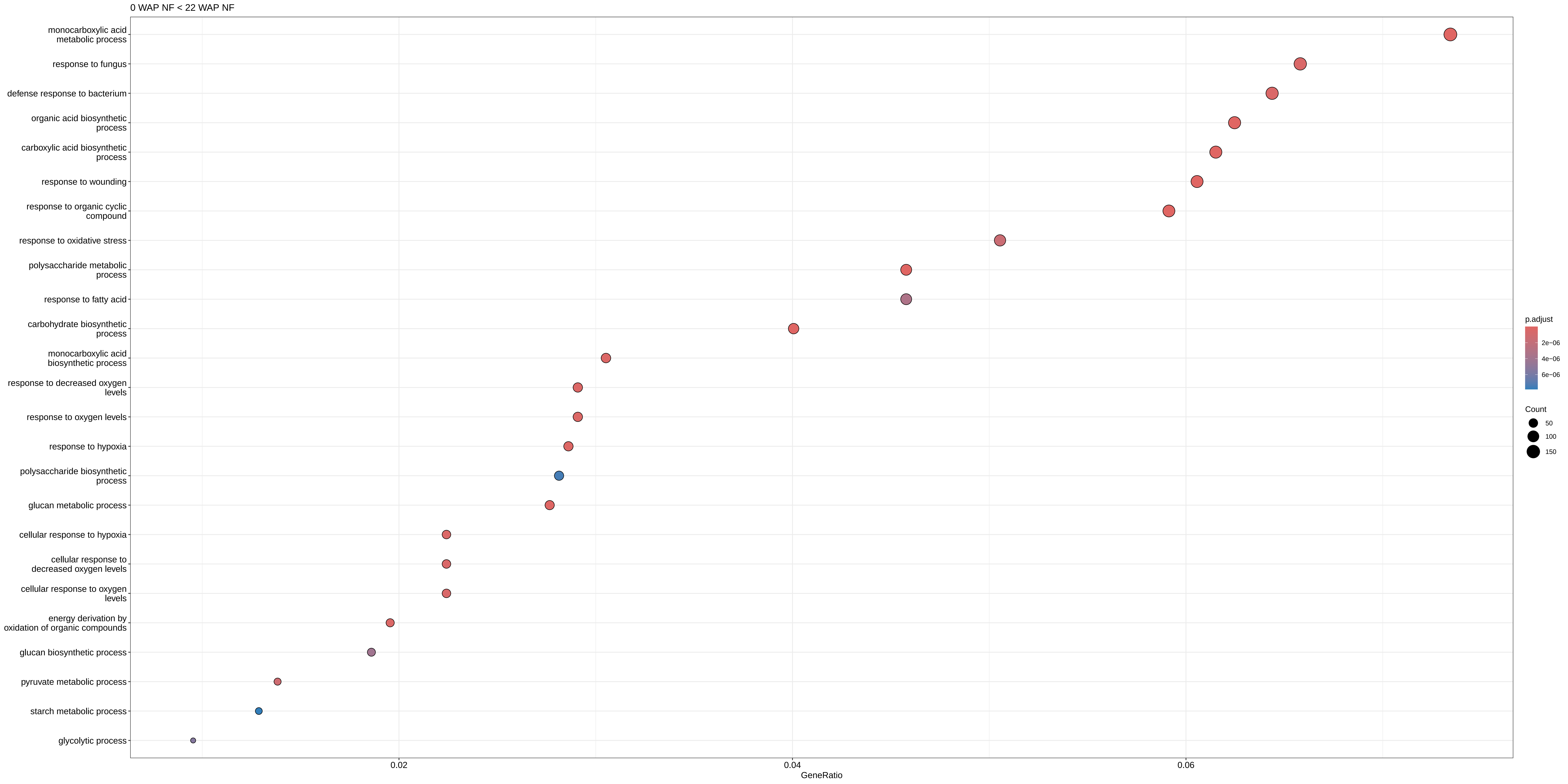
0 WAP NF < 22 WAP NF
monocarboxylic acid metabolic process
response to fungus
defense response to bacterium
organic acid biosynthetic process
carboxylic acid biosynthetic process
response to wounding
response to organic cyclic compound
response to oxidative stress
polysaccharide metabolic process
response to fatty acid
carbohydrate biosynthetic process
monocarboxylic acid biosynthetic process
response to decreased oxygen levels
response to oxygen levels
response to hypoxia
polysaccharide biosynthetic process
glucan metabolic process
cellular response to hypoxia
cellular response to decreased oxygen levels
cellular response to oxygen levels
energy derivation by oxidation of organic compounds
glucan biosynthetic process
pyruvate metabolic process
starch metabolic process
glycolytic process
0.02
0.04
0.06
GeneRatio
p.adjust
2e−06
4e−06
6e−06
Count
50
100
150

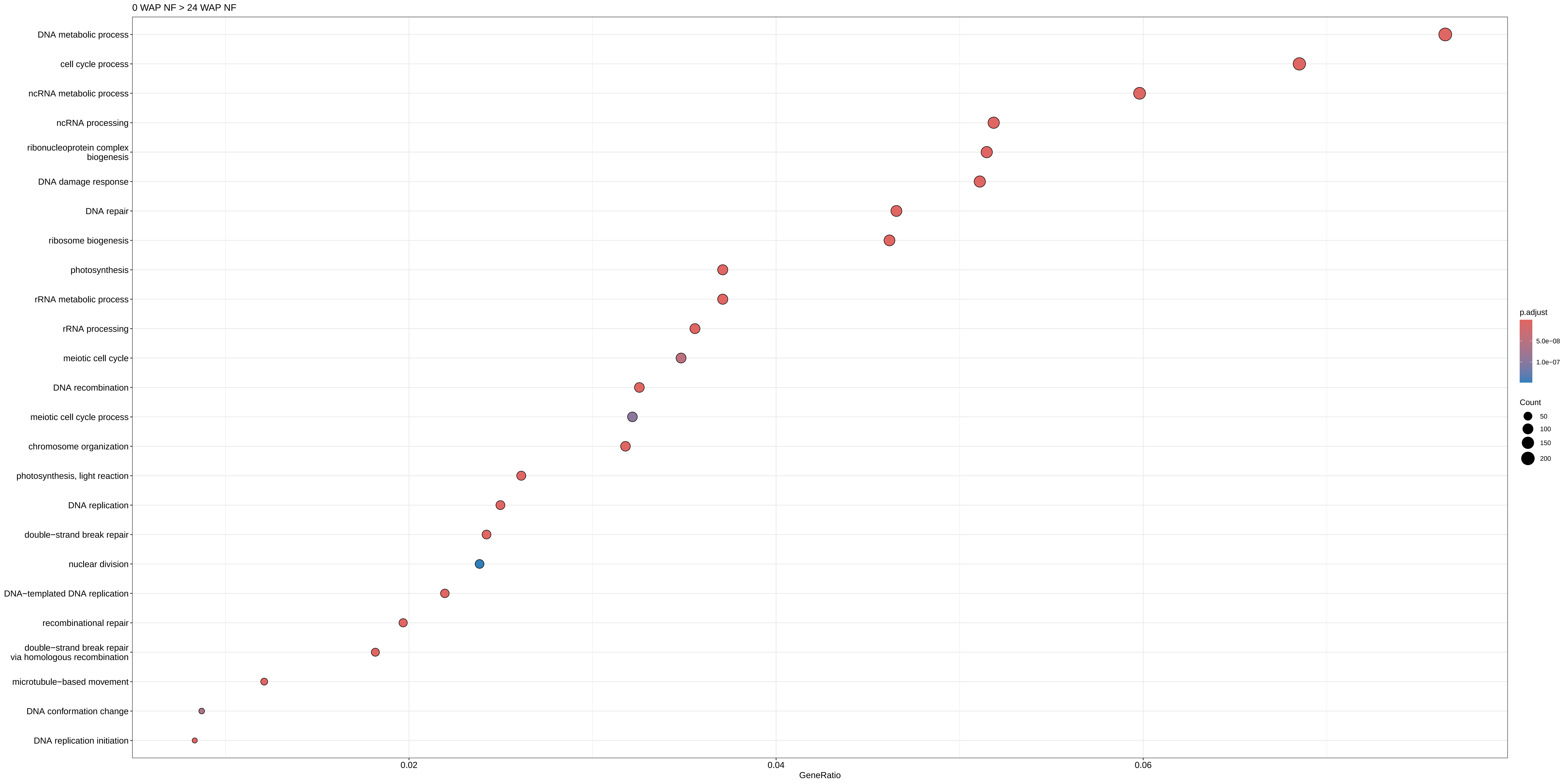

0 WAP NF > 24 WAP NF
DNA metabolic process
cell cycle process
ncRNA metabolic process
ncRNA processing
ribonucleoprotein complex biogenesis
DNA damage response
DNA repair
ribosome biogenesis
photosynthesis
rRNA metabolic process
rRNA processing
meiotic cell cycle
DNA recombination
meiotic cell cycle process
chromosome organization
photosynthesis, light reaction
DNA replication
double−strand break repair
nuclear division
DNA−templated DNA replication
recombinational repair
double−strand break repair via homologous recombination
microtubule−based movement
DNA conformation change
DNA replication initiation
0.02
0.04
0.06
GeneRatio
p.adjust
5.0e−08
1.0e−07
Count
50
100
150
200

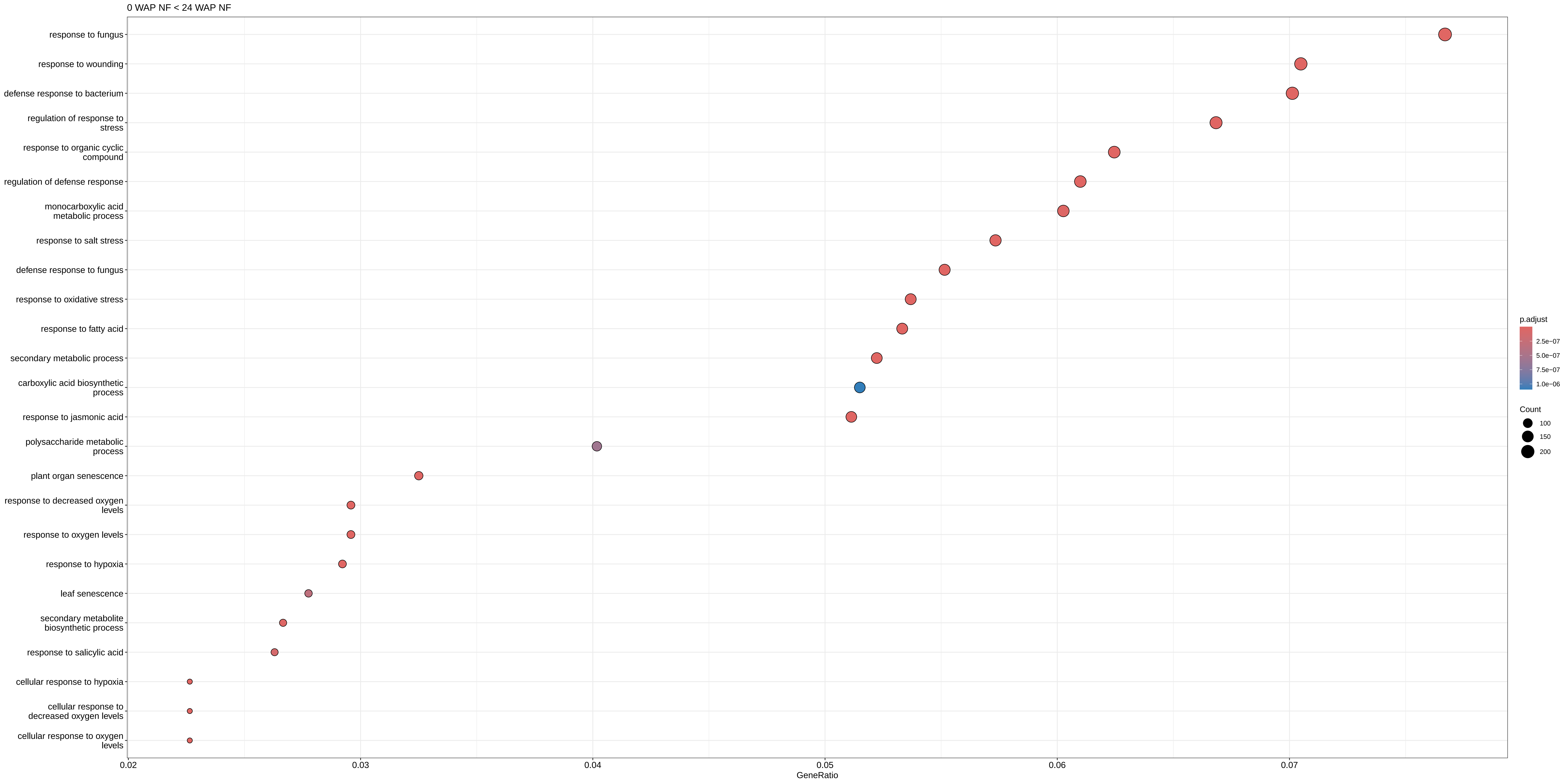

0 WAP NF < 24 WAP NF
response to fungus
response to wounding
defense response to bacterium
regulation of response to stress
response to organic cyclic compound
regulation of defense response
monocarboxylic acid metabolic process
response to salt stress
defense response to fungus
response to oxidative stress
response to fatty acid
secondary metabolic process
carboxylic acid biosynthetic process
response to jasmonic acid
polysaccharide metabolic process
plant organ senescence
response to decreased oxygen levels
response to oxygen levels
response to hypoxia
leaf senescence
secondary metabolite biosynthetic process
response to salicylic acid
cellular response to hypoxia
cellular response to decreased oxygen levels
cellular response to oxygen levels
0.02
0.03
0.04
0.05
0.06
0.07
GeneRatio
p.adjust
2.5e−07
5.0e−07
7.5e−07
1.0e−06
Count
100
150
200

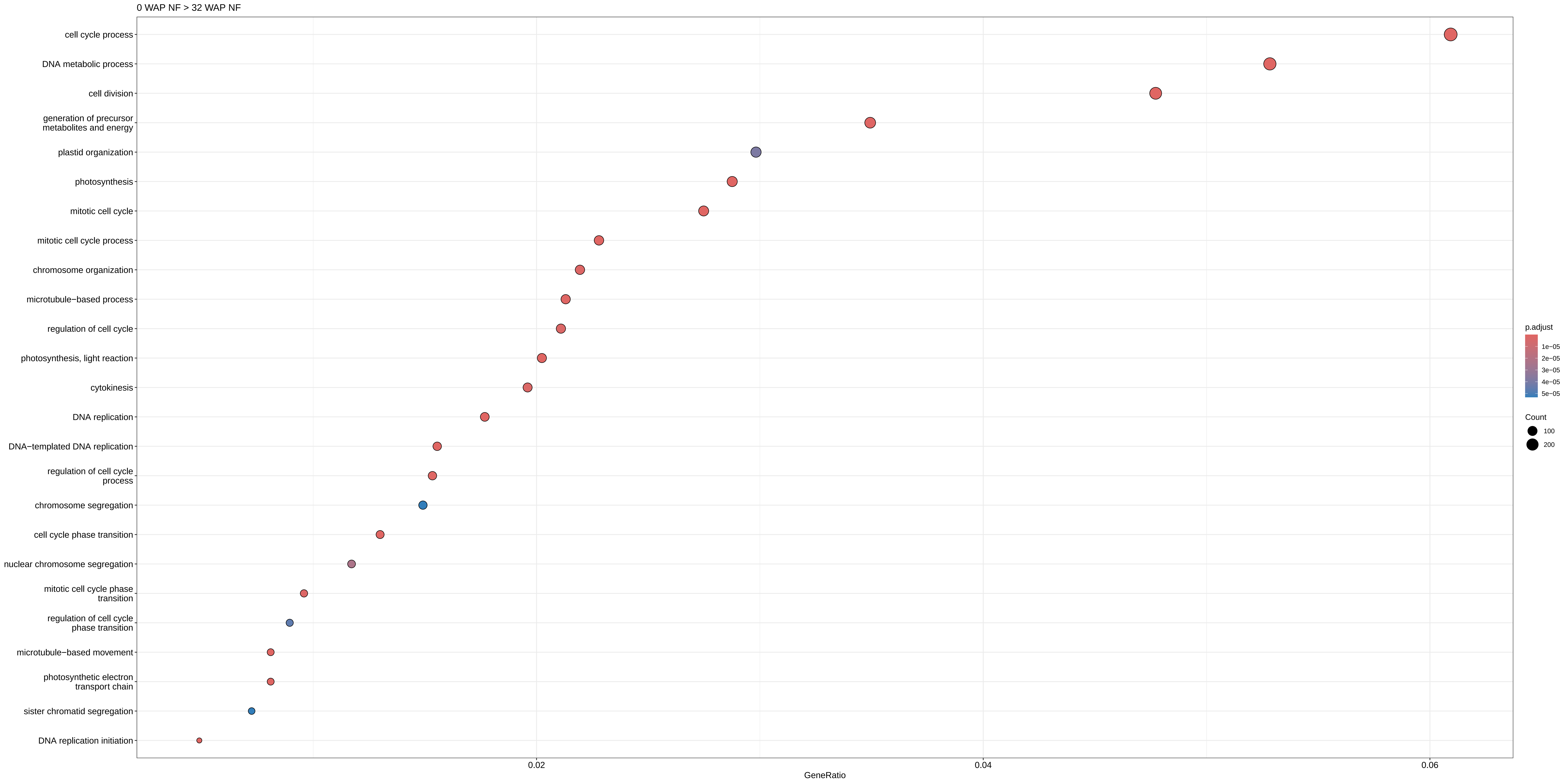

0 WAP NF > 32 WAP NF
cell cycle process
DNA metabolic process
cell division
generation of precursor metabolites and energy
plastid organization
photosynthesis
mitotic cell cycle
mitotic cell cycle process
chromosome organization
microtubule−based process
regulation of cell cycle
photosynthesis, light reaction
cytokinesis
DNA replication
DNA−templated DNA replication
regulation of cell cycle process
chromosome segregation
cell cycle phase transition
nuclear chromosome segregation
mitotic cell cycle phase transition
regulation of cell cycle phase transition
microtubule−based movement
photosynthetic electron transport chain
sister chromatid segregation
DNA replication initiation
0.02
0.04
0.06
GeneRatio
p.adjust
1e−05
2e−05
3e−05
4e−05
5e−05
Count
100
200

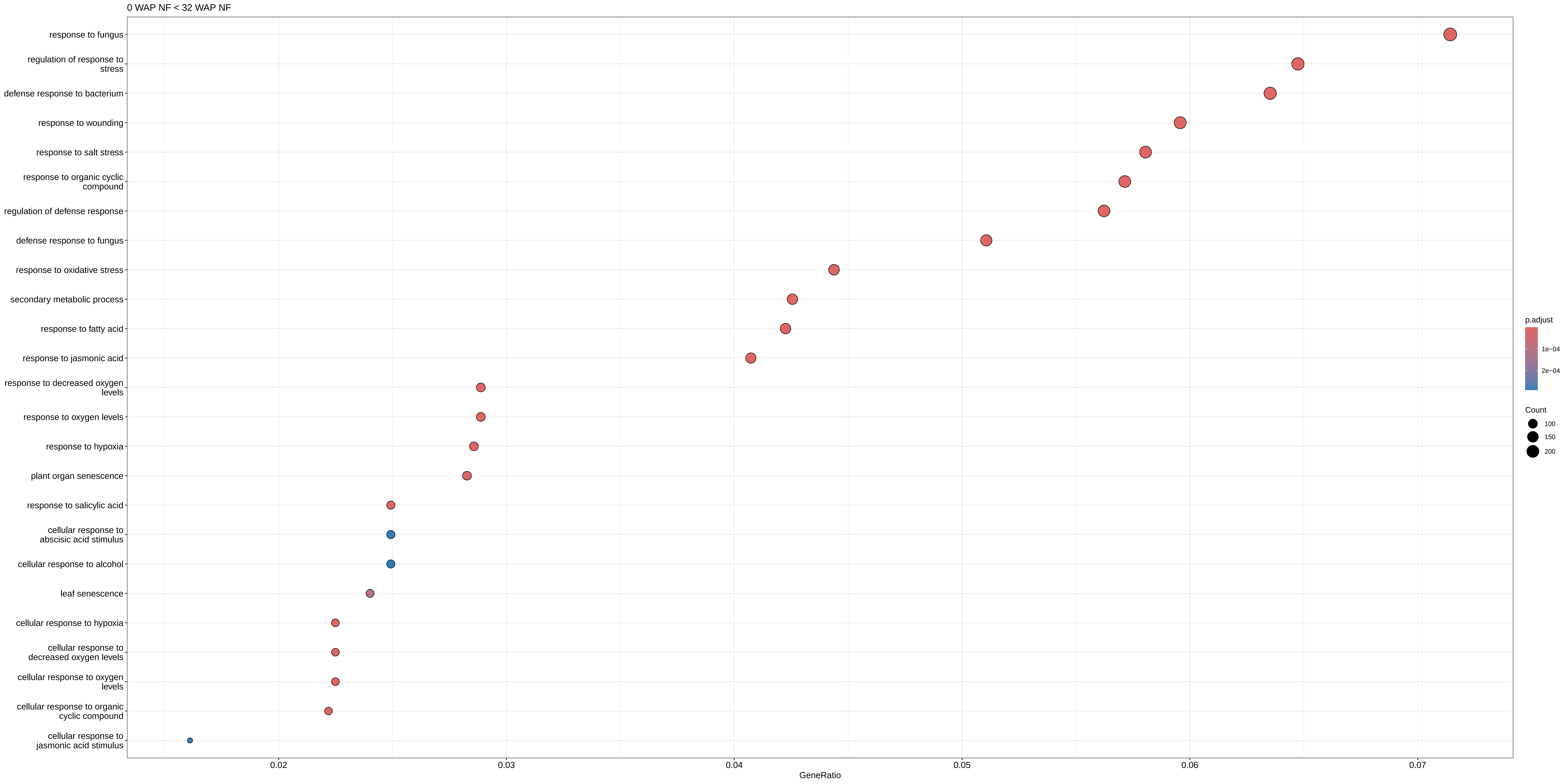

0 WAP NF < 32 WAP NF
response to fungus
regulation of response to stress
defense response to bacterium
response to wounding
response to salt stress
response to organic cyclic compound
regulation of defense response
defense response to fungus
response to oxidative stress
secondary metabolic process
response to fatty acid
response to jasmonic acid
response to decreased oxygen levels
response to oxygen levels
response to hypoxia
plant organ senescence
response to salicylic acid
cellular response to abscisic acid stimulus
cellular response to alcohol
leaf senescence
cellular response to hypoxia
cellular response to decreased oxygen levels
cellular response to oxygen levels
cellular response to organic cyclic compound
cellular response to jasmonic acid stimulus
0.02
0.03
0.04
0.05
0.06
0.07
GeneRatio
p.adjust
1e−04
2e−04
Count
100
150
200

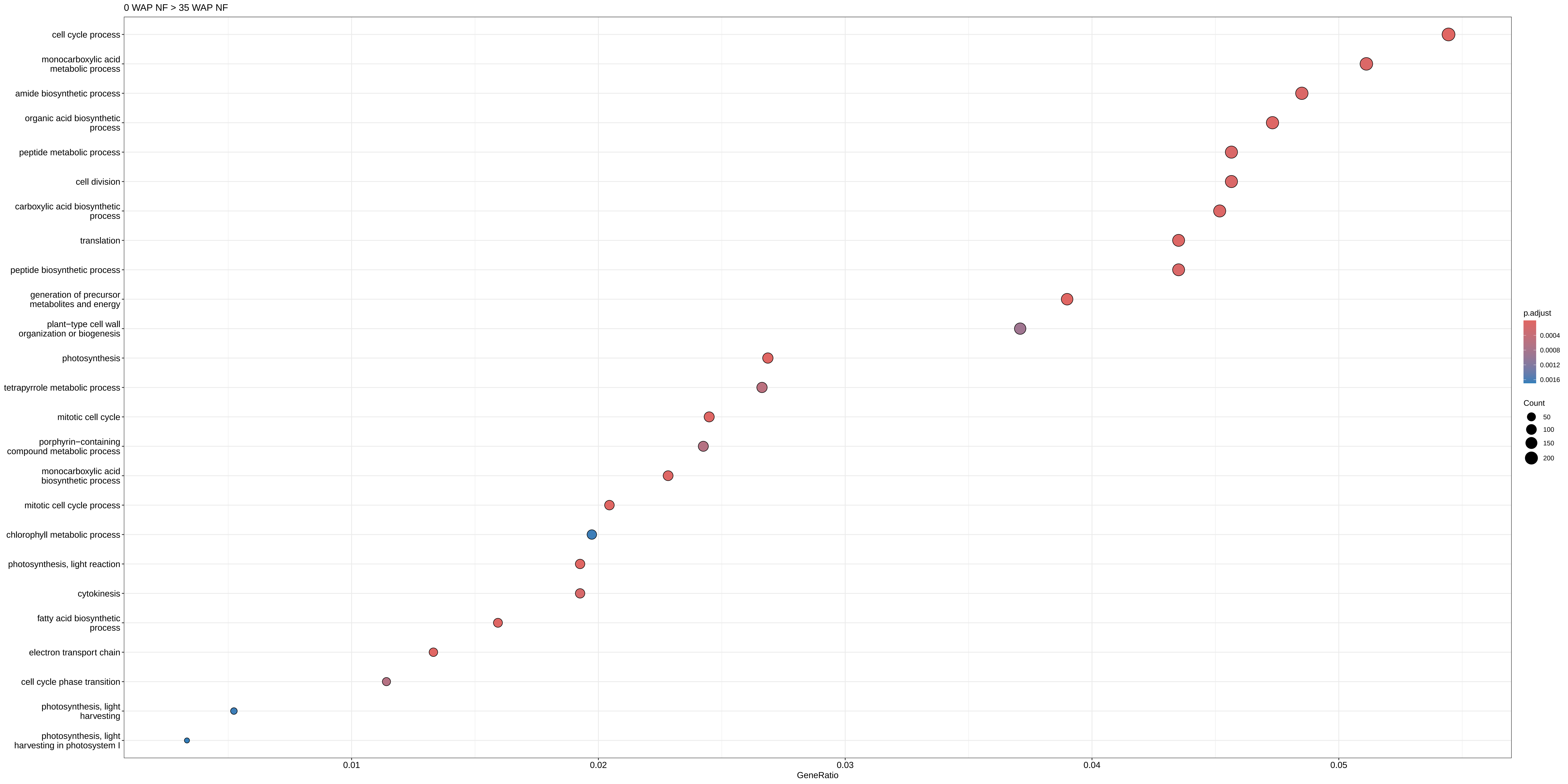

0 WAP NF > 35 WAP NF
cell cycle process
monocarboxylic acid metabolic process
amide biosynthetic process
organic acid biosynthetic process
peptide metabolic process
cell division
carboxylic acid biosynthetic process
translation
peptide biosynthetic process
generation of precursor metabolites and energy
plant−type cell wall organization or biogenesis
photosynthesis
tetrapyrrole metabolic process
mitotic cell cycle
porphyrin−containing compound metabolic process
monocarboxylic acid biosynthetic process
mitotic cell cycle process
chlorophyll metabolic process
photosynthesis, light reaction
cytokinesis
fatty acid biosynthetic process
electron transport chain
cell cycle phase transition
photosynthesis, light harvesting
photosynthesis, light harvesting in photosystem I
0.01
0.02
0.03
0.04
0.05
GeneRatio
p.adjust
0.0004
0.0008
0.0012
0.0016
Count
50
100
150
200

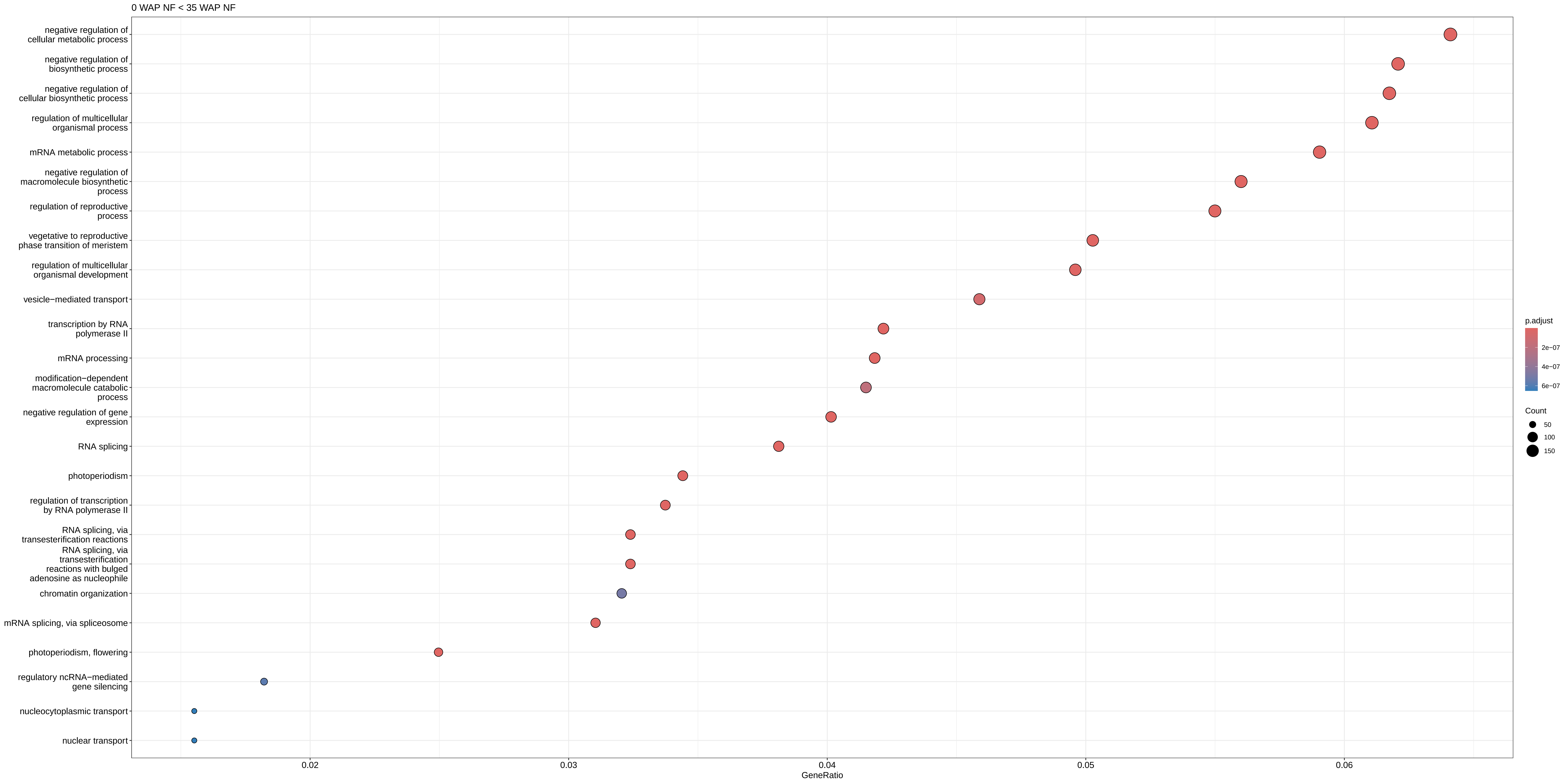

0 WAP NF < 35 WAP NF
negative regulation of cellular metabolic process
negative regulation of biosynthetic process
negative regulation of cellular biosynthetic process
regulation of multicellular organismal process
mRNA metabolic process
negative regulation of macromolecule biosynthetic process
regulation of reproductive process
vegetative to reproductive phase transition of meristem
regulation of multicellular organismal development
vesicle−mediated transport
transcription by RNA polymerase II
mRNA processing
modification−dependent macromolecule catabolic process
negative regulation of gene expression
RNA splicing
photoperiodism
regulation of transcription by RNA polymerase II
RNA splicing, via transesterification reactions
RNA splicing, via transesterification reactions with bulged adenosine as nucleophile
chromatin organization
mRNA splicing, via spliceosome
photoperiodism, flowering
regulatory ncRNA−mediated gene silencing
nucleocytoplasmic transport
nuclear transport
0.02
0.03
0.04
0.05
0.06
GeneRatio
p.adjust
2e−07
4e−07
6e−07
Count
50
100
150

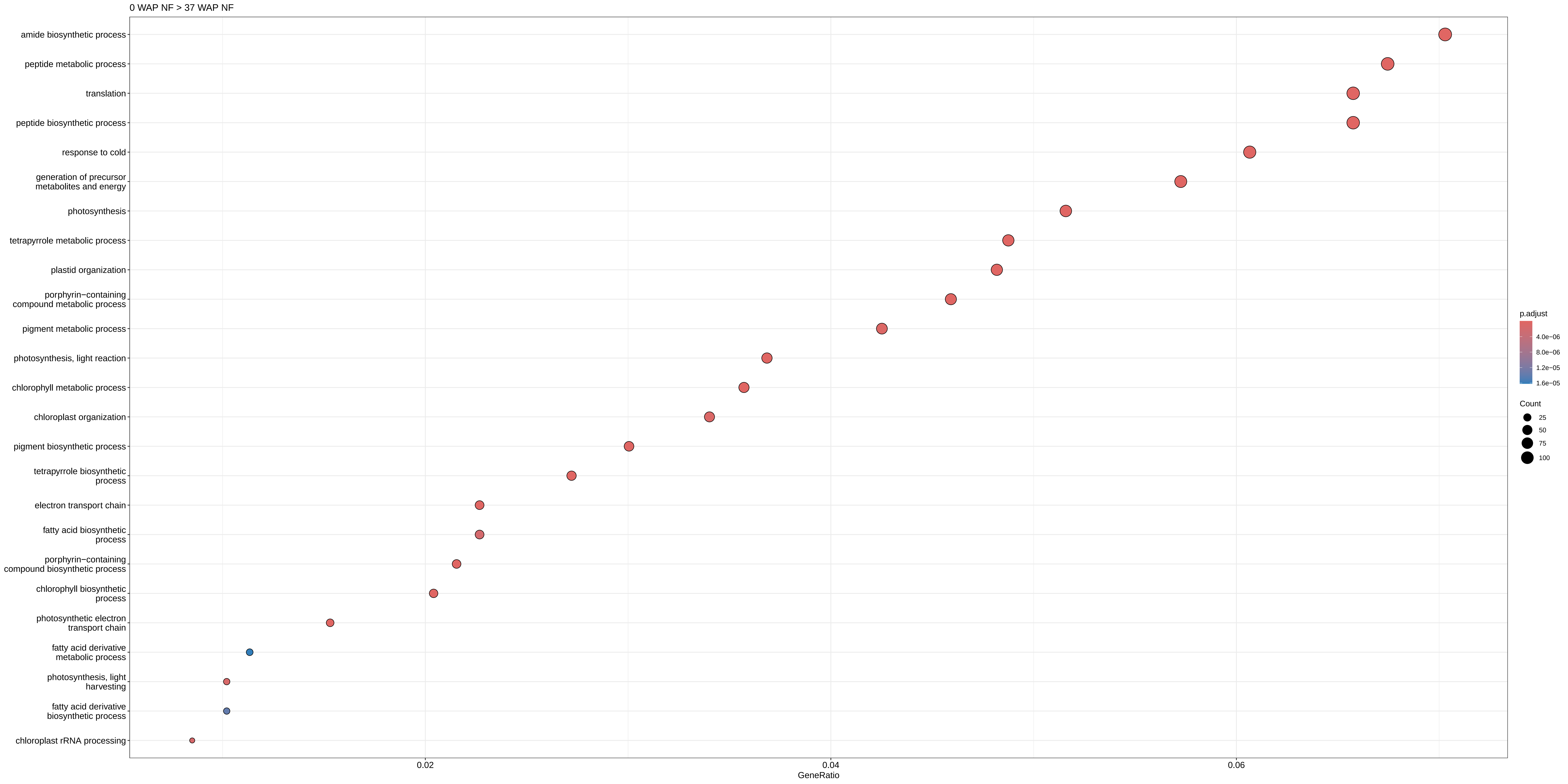
0 WAP NF > 37 WAP NF
amide biosynthetic process
peptide metabolic process
translation
peptide biosynthetic process
response to cold
generation of precursor metabolites and energy
photosynthesis
tetrapyrrole metabolic process
plastid organization
porphyrin−containing compound metabolic process
pigment metabolic process
photosynthesis, light reaction
chlorophyll metabolic process
chloroplast organization
pigment biosynthetic process
tetrapyrrole biosynthetic process
electron transport chain
fatty acid biosynthetic process
porphyrin−containing compound biosynthetic process
chlorophyll biosynthetic process
photosynthetic electron transport chain
fatty acid derivative metabolic process
photosynthesis, light harvesting
fatty acid derivative biosynthetic process
chloroplast rRNA processing
0.02
0.04
0.06
GeneRatio
p.adjust
4.0e−06
8.0e−06
1.2e−05
1.6e−05
Count
25
50
75
100

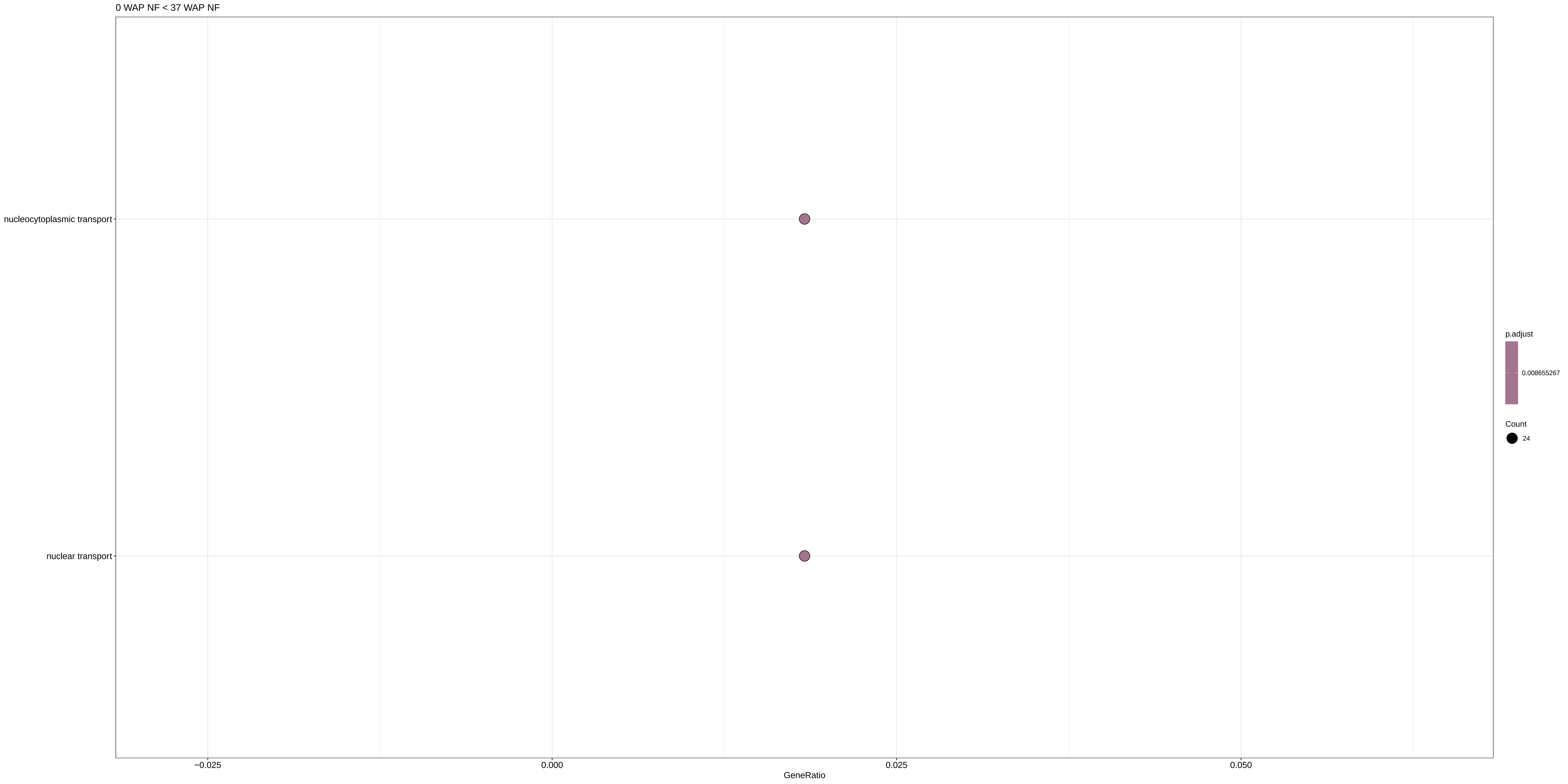
0 WAP NF < 37 WAP NF
nucleocytoplasmic transport
nuclear transport
−0.025
0.000
0.025
0.050
GeneRatio
p.adjust
0.008655267
Count
24

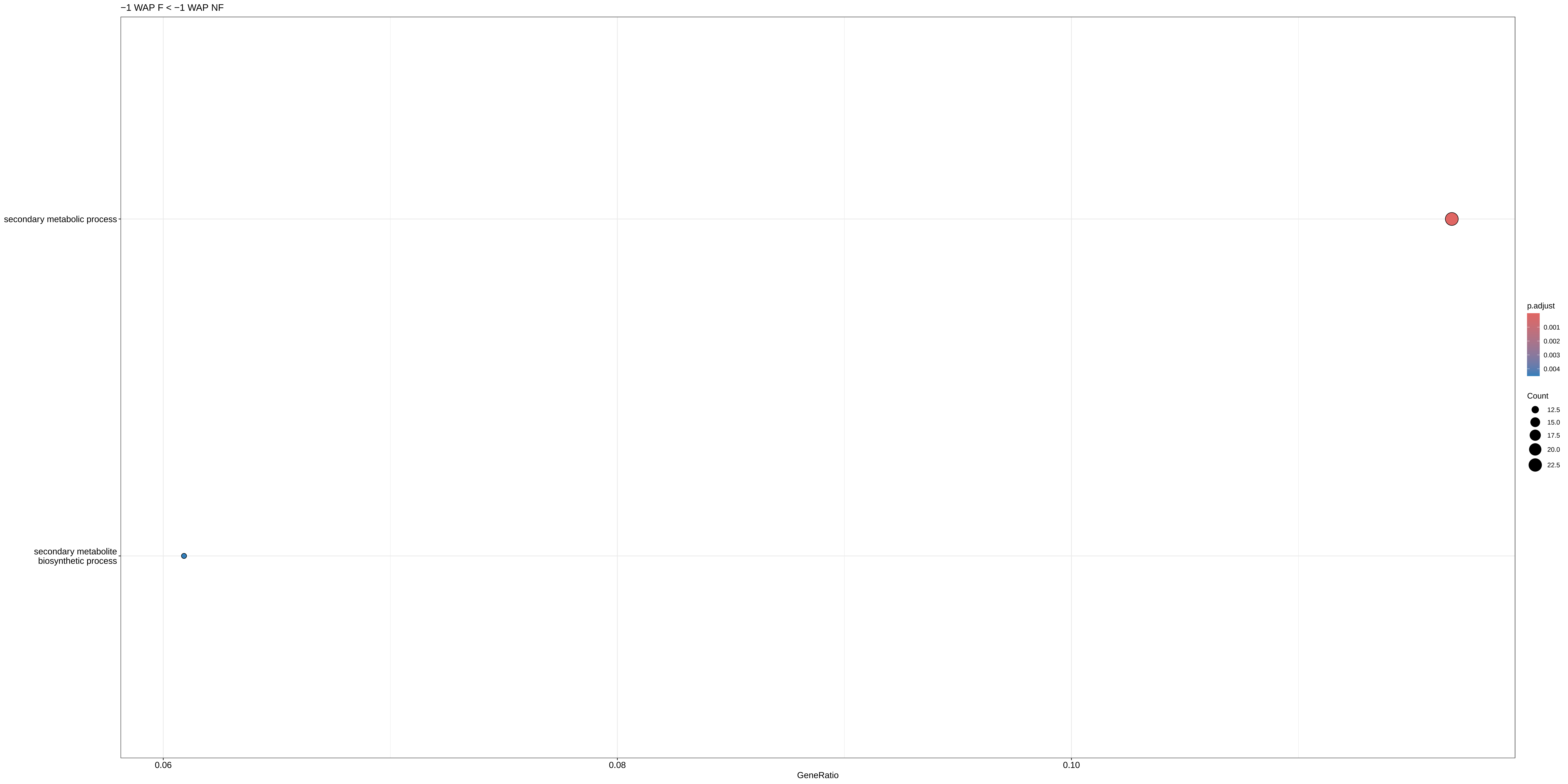
−1 WAP F < −1 WAP NF
secondary metabolic process
secondary metabolite biosynthetic process
0.06
0.08
0.10
GeneRatio
p.adjust
0.001
0.002
0.003
0.004
Count
12.5
15.0
17.5
20.0
22.5

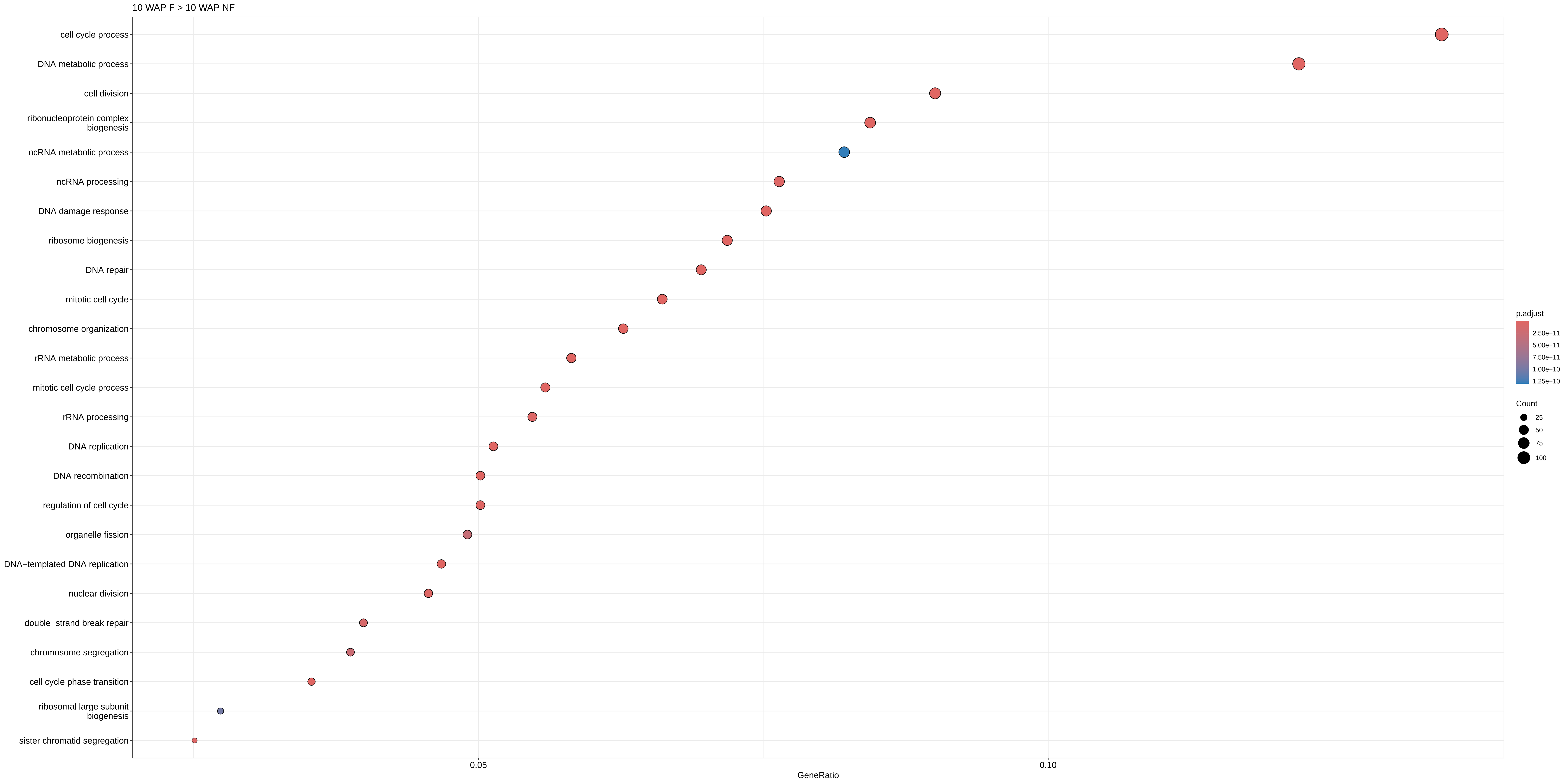
10 WAP F > 10 WAP NF
cell cycle process
DNA metabolic process
cell division
ribonucleoprotein complex biogenesis
ncRNA metabolic process
ncRNA processing
DNA damage response
ribosome biogenesis
DNA repair
mitotic cell cycle
chromosome organization
rRNA metabolic process
mitotic cell cycle process
rRNA processing
DNA replication
DNA recombination
regulation of cell cycle
organelle fission
DNA−templated DNA replication
nuclear division
double−strand break repair
chromosome segregation
cell cycle phase transition
ribosomal large subunit biogenesis
sister chromatid segregation
0.05
0.10
GeneRatio
p.adjust
2.50e−11
5.00e−11
7.50e−11
1.00e−10
1.25e−10
Count
25
50
75
100

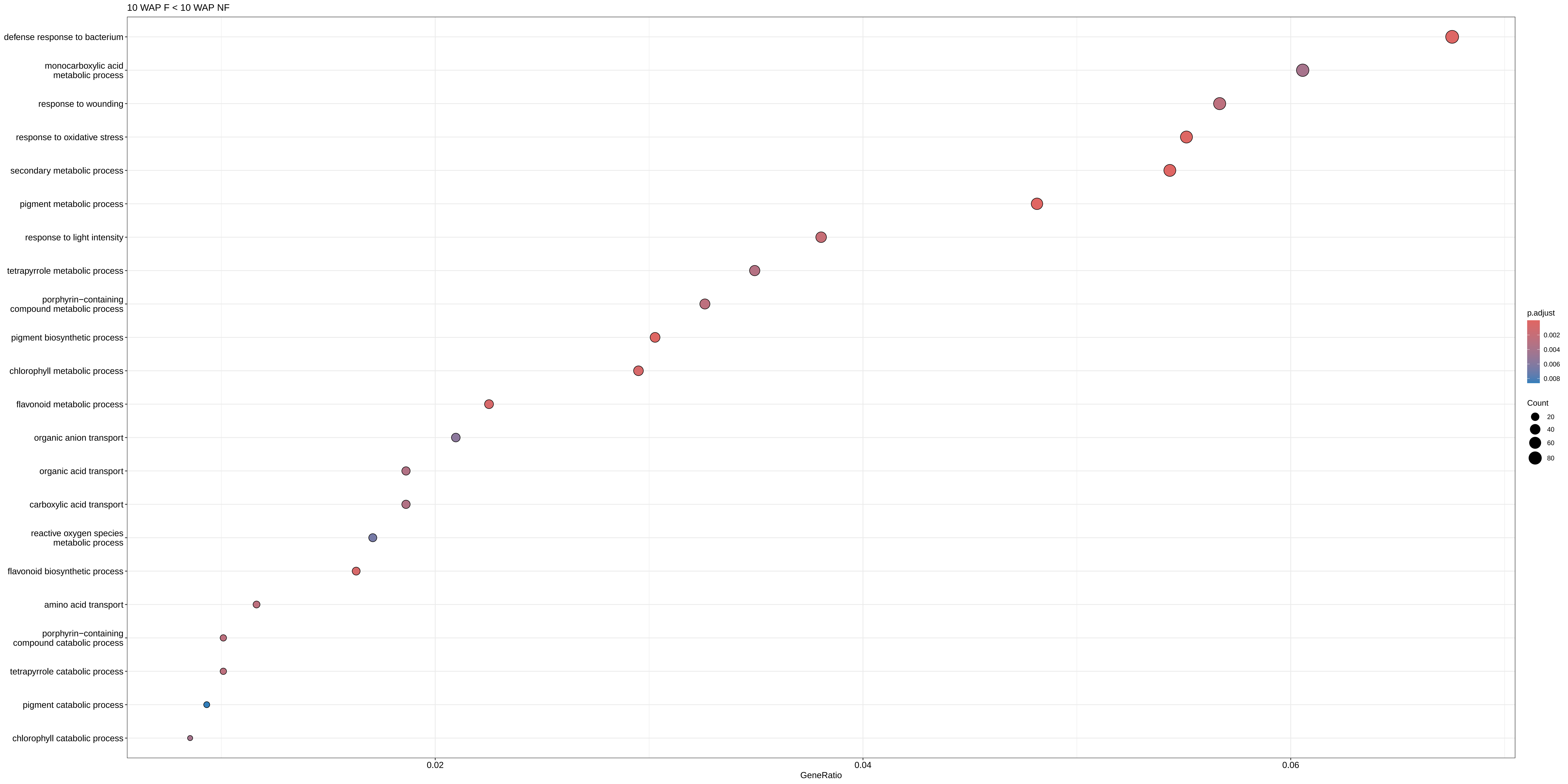

10 WAP F < 10 WAP NF
defense response to bacterium
monocarboxylic acid metabolic process
response to wounding
response to oxidative stress
secondary metabolic process
pigment metabolic process
response to light intensity
tetrapyrrole metabolic process
porphyrin−containing compound metabolic process
pigment biosynthetic process
chlorophyll metabolic process
flavonoid metabolic process
organic anion transport
organic acid transport
carboxylic acid transport
reactive oxygen species metabolic process
flavonoid biosynthetic process
amino acid transport
porphyrin−containing compound catabolic process
tetrapyrrole catabolic process
pigment catabolic process
chlorophyll catabolic process
0.02
0.04
0.06
GeneRatio
p.adjust
0.002
0.004
0.006
0.008
Count
20
40
60
80

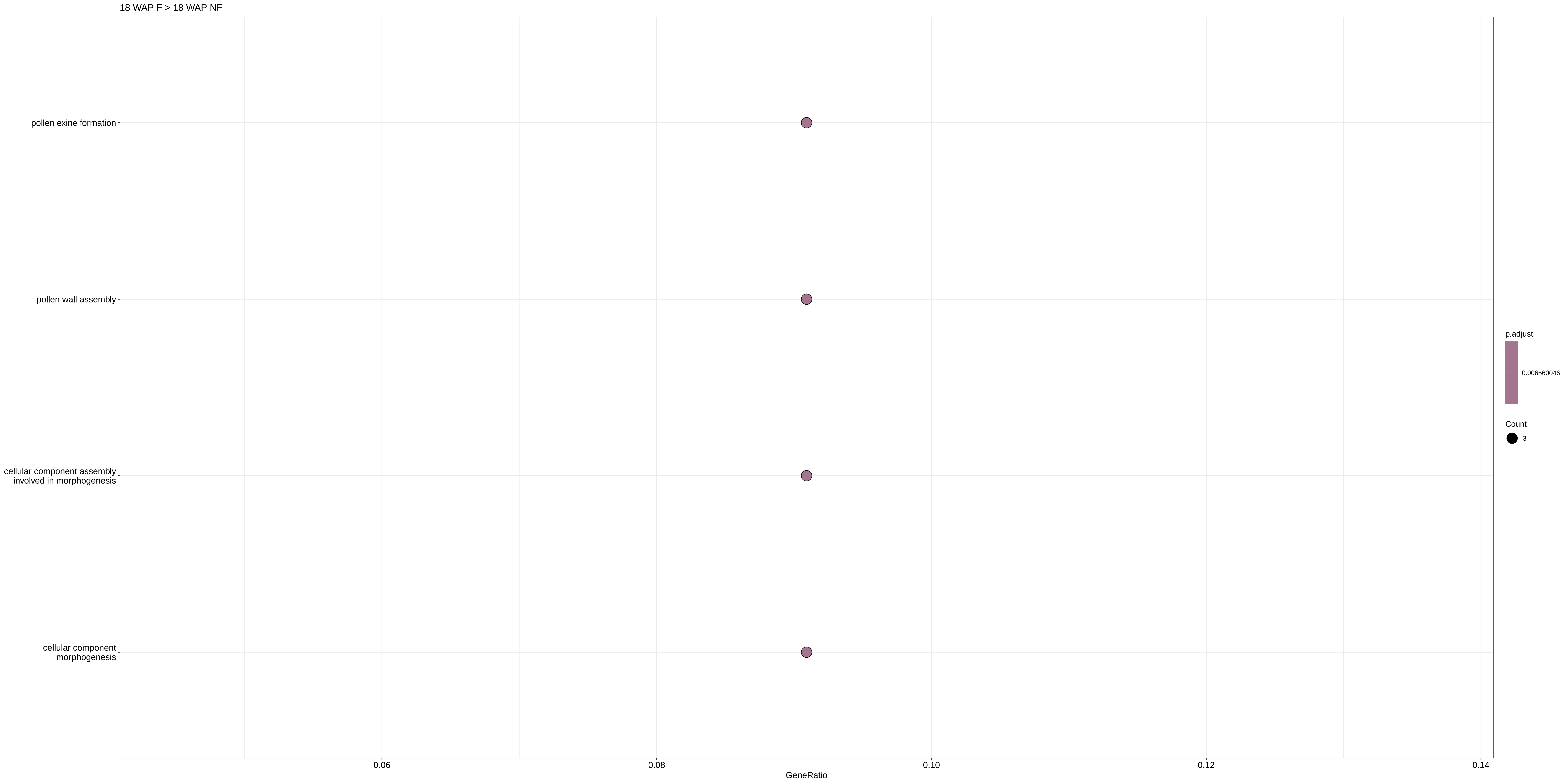
18 WAP F > 18 WAP NF
pollen exine formation
pollen wall assembly
cellular component assembly
involved in morphogenesis
cellular component
morphogenesis
0.06
0.08
0.10
0.12
0.14
GeneRatio
p.adjust
0.006560046
Count
3

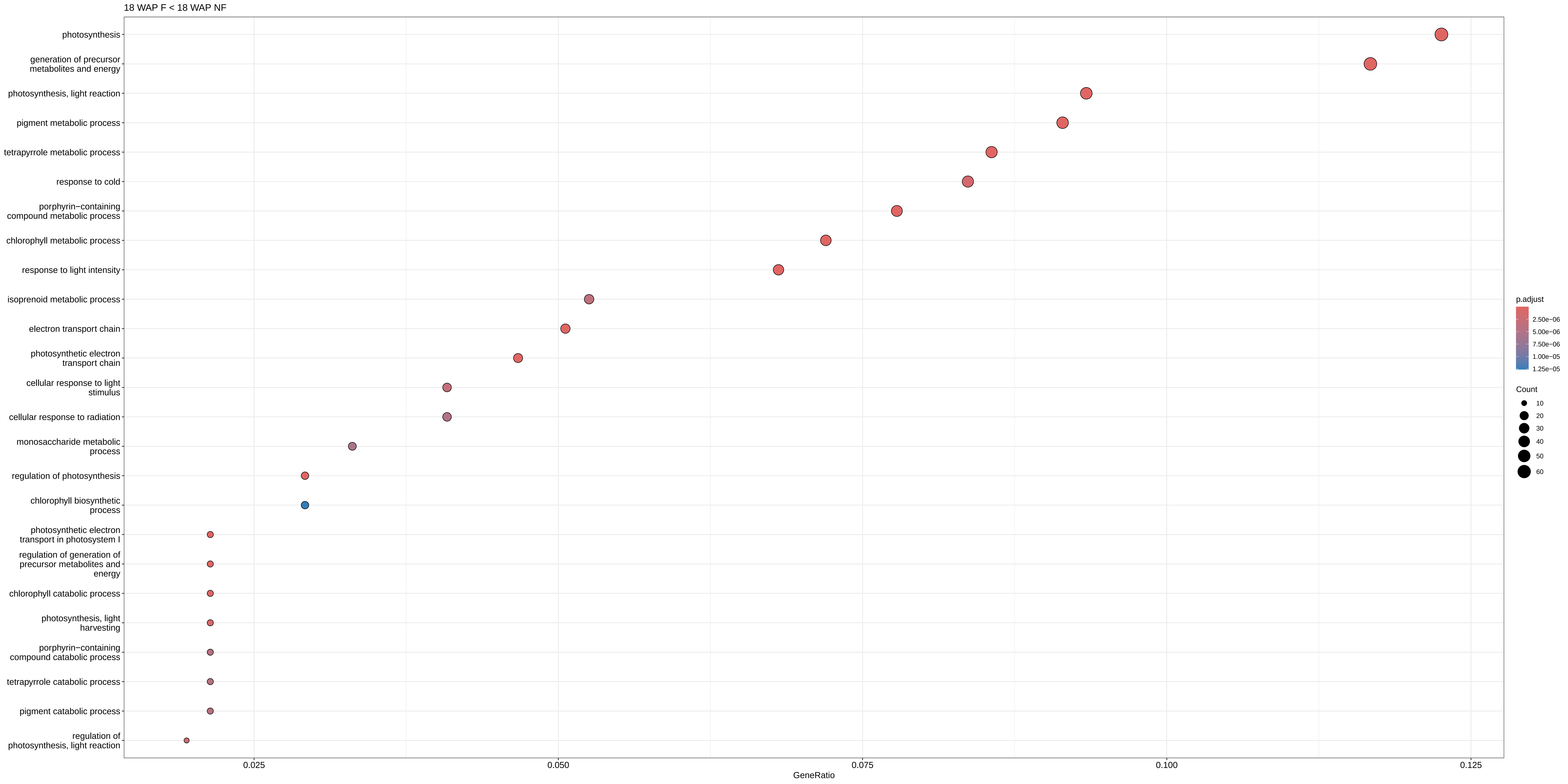
18 WAP F < 18 WAP NF
photosynthesis
generation of precursor metabolites and energy
photosynthesis, light reaction
pigment metabolic process
tetrapyrrole metabolic process
response to cold
porphyrin−containing compound metabolic process
chlorophyll metabolic process
response to light intensity
isoprenoid metabolic process
electron transport chain
photosynthetic electron transport chain
cellular response to light stimulus
cellular response to radiation
monosaccharide metabolic process
regulation of photosynthesis
chlorophyll biosynthetic process
photosynthetic electron transport in photosystem I
regulation of generation of precursor metabolites and energy
chlorophyll catabolic process
photosynthesis, light harvesting
porphyrin−containing compound catabolic process
tetrapyrrole catabolic process
pigment catabolic process
regulation of photosynthesis, light reaction
0.025
0.050
0.075
0.100
0.125
GeneRatio
p.adjust
2.50e−06
5.00e−06
7.50e−06
1.00e−05
1.25e−05
Count
10
20
30
40
50
60

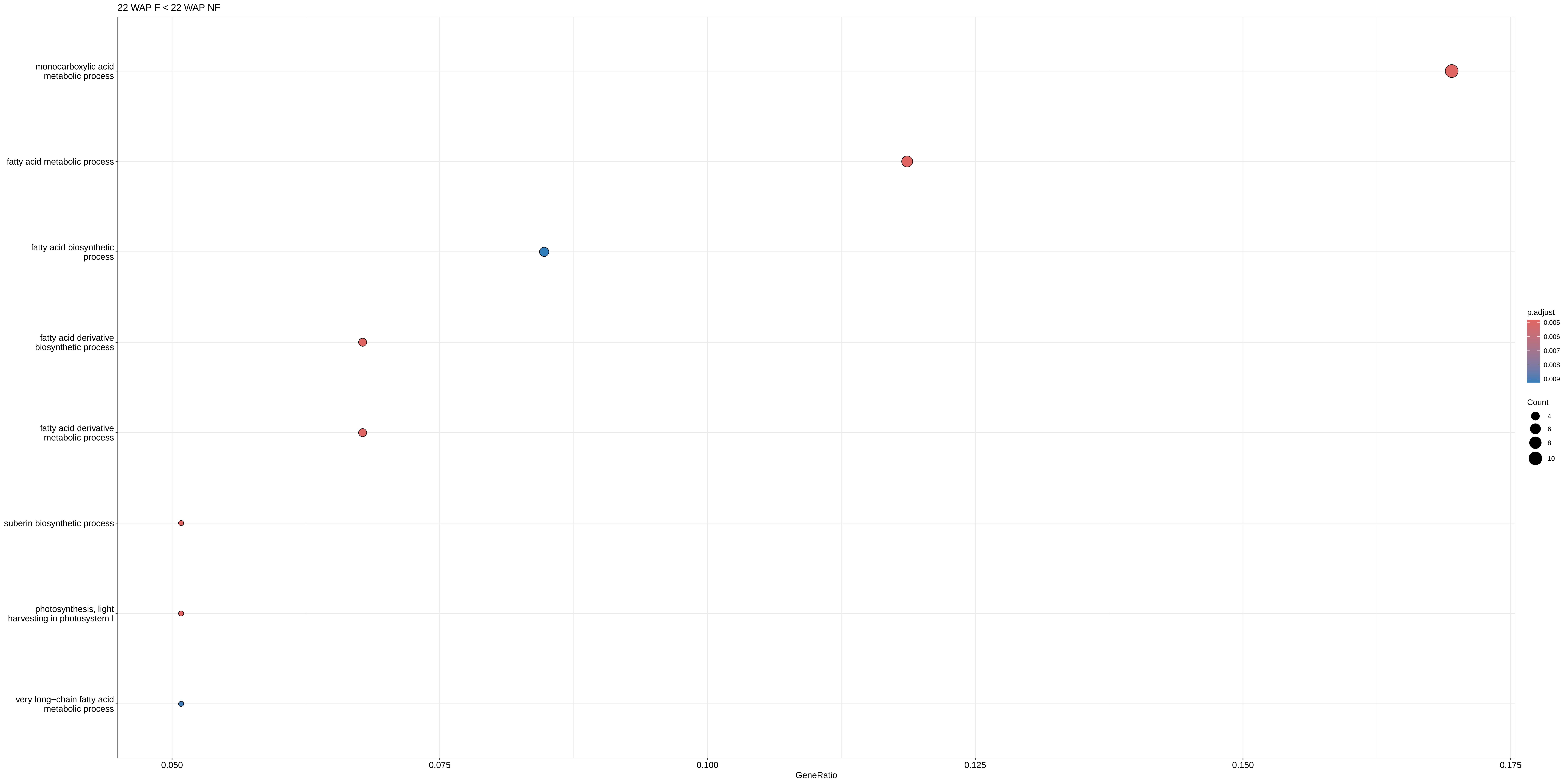

22 WAP F < 22 WAP NF
monocarboxylic acid metabolic process
fatty acid metabolic process
fatty acid biosynthetic process
fatty acid derivative biosynthetic process
fatty acid derivative metabolic process
suberin biosynthetic process
photosynthesis, light harvesting in photosystem I
very long−chain fatty acid metabolic process
0.050
0.075
0.100
0.125
0.150
0.175
GeneRatio
p.adjust
0.005
0.006
0.007
0.008
0.009
Count
4
6
8
10

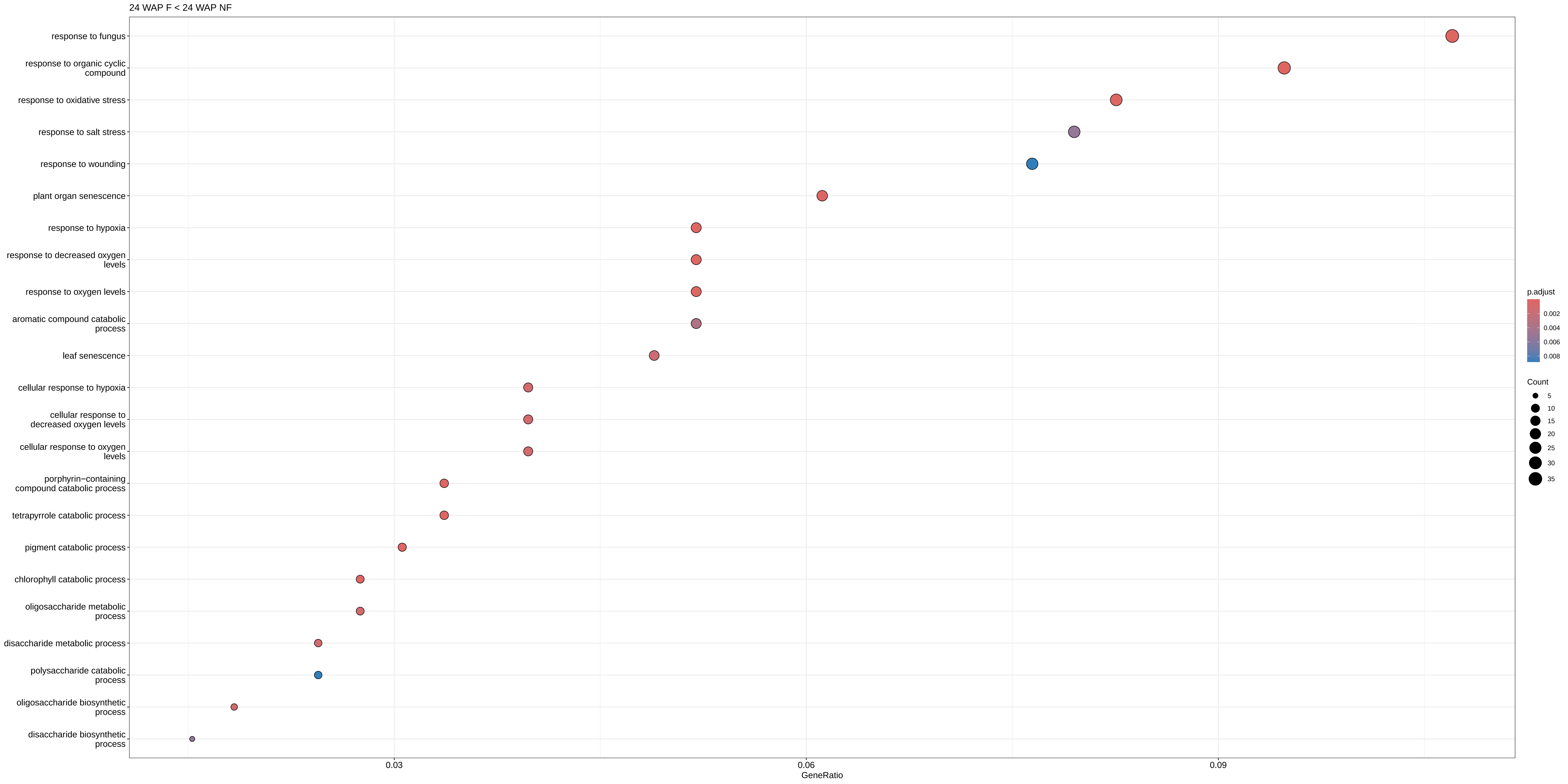
24 WAP F < 24 WAP NF
response to fungus
response to organic cyclic compound
response to oxidative stress
response to salt stress
response to wounding
plant organ senescence
response to hypoxia
response to decreased oxygen levels
response to oxygen levels
aromatic compound catabolic process
leaf senescence
cellular response to hypoxia
cellular response to decreased oxygen levels
cellular response to oxygen levels
porphyrin−containing compound catabolic process
tetrapyrrole catabolic process
pigment catabolic process
chlorophyll catabolic process
oligosaccharide metabolic process
disaccharide metabolic process
polysaccharide catabolic process
oligosaccharide biosynthetic process
disaccharide biosynthetic process
0.03
0.06
0.09
GeneRatio
p.adjust
0.002
0.004
0.006
0.008
Count
5
10
15
20
25
30
35

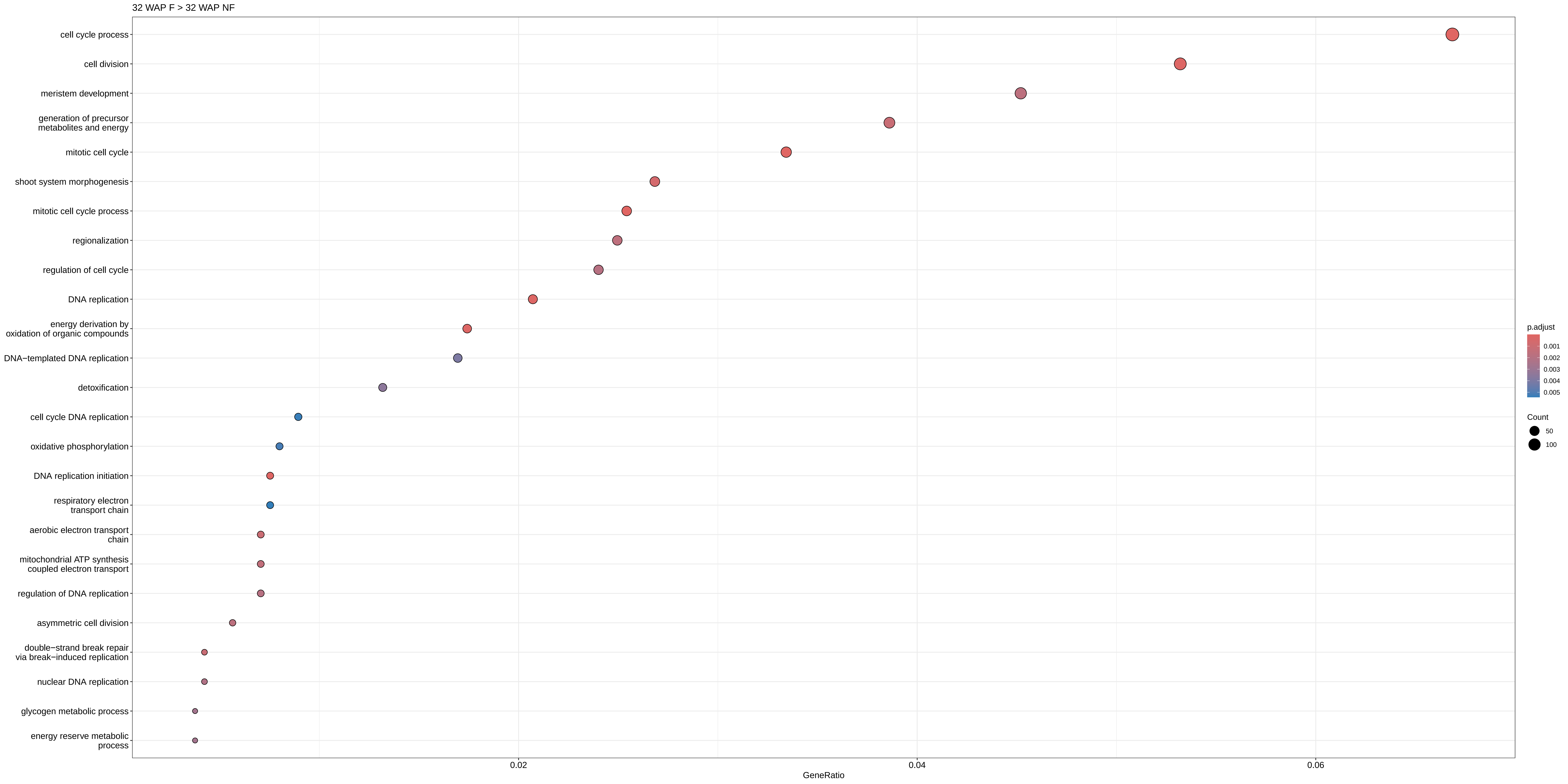
32 WAP F > 32 WAP NF
cell cycle process
cell division
meristem development
generation of precursor metabolites and energy
mitotic cell cycle
shoot system morphogenesis
mitotic cell cycle process
regionalization
regulation of cell cycle
DNA replication
energy derivation by oxidation of organic compounds
DNA−templated DNA replication
detoxification
cell cycle DNA replication
oxidative phosphorylation
DNA replication initiation
respiratory electron transport chain
aerobic electron transport chain
mitochondrial ATP synthesis coupled electron transport
regulation of DNA replication
asymmetric cell division
double−strand break repair via break−induced replication
nuclear DNA replication
glycogen metabolic process
energy reserve metabolic process
0.02
0.04
0.06
GeneRatio
p.adjust
0.001
0.002
0.003
0.004
0.005
Count
50
100

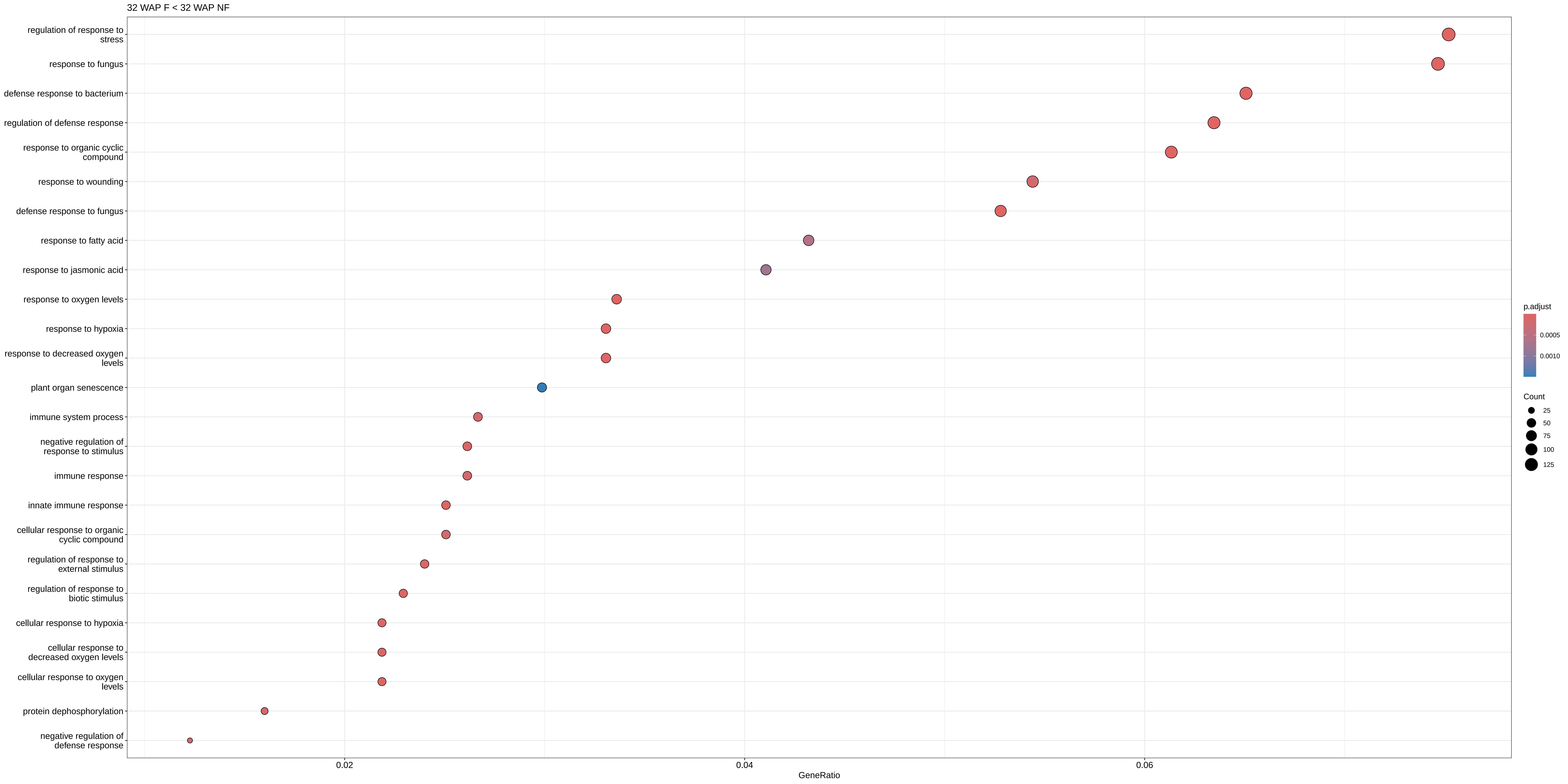

32 WAP F < 32 WAP NF
regulation of response to stress
response to fungus
defense response to bacterium
regulation of defense response
response to organic cyclic compound
response to wounding
defense response to fungus
response to fatty acid
response to jasmonic acid
response to oxygen levels
response to hypoxia
response to decreased oxygen levels
plant organ senescence
immune system process
negative regulation of response to stimulus
immune response
innate immune response
cellular response to organic cyclic compound
regulation of response to external stimulus
regulation of response to biotic stimulus
cellular response to hypoxia
cellular response to decreased oxygen levels
cellular response to oxygen levels
protein dephosphorylation
negative regulation of defense response
0.02
0.04
0.06
GeneRatio
p.adjust
0.0005
0.0010
Count
25
50
75
100
125

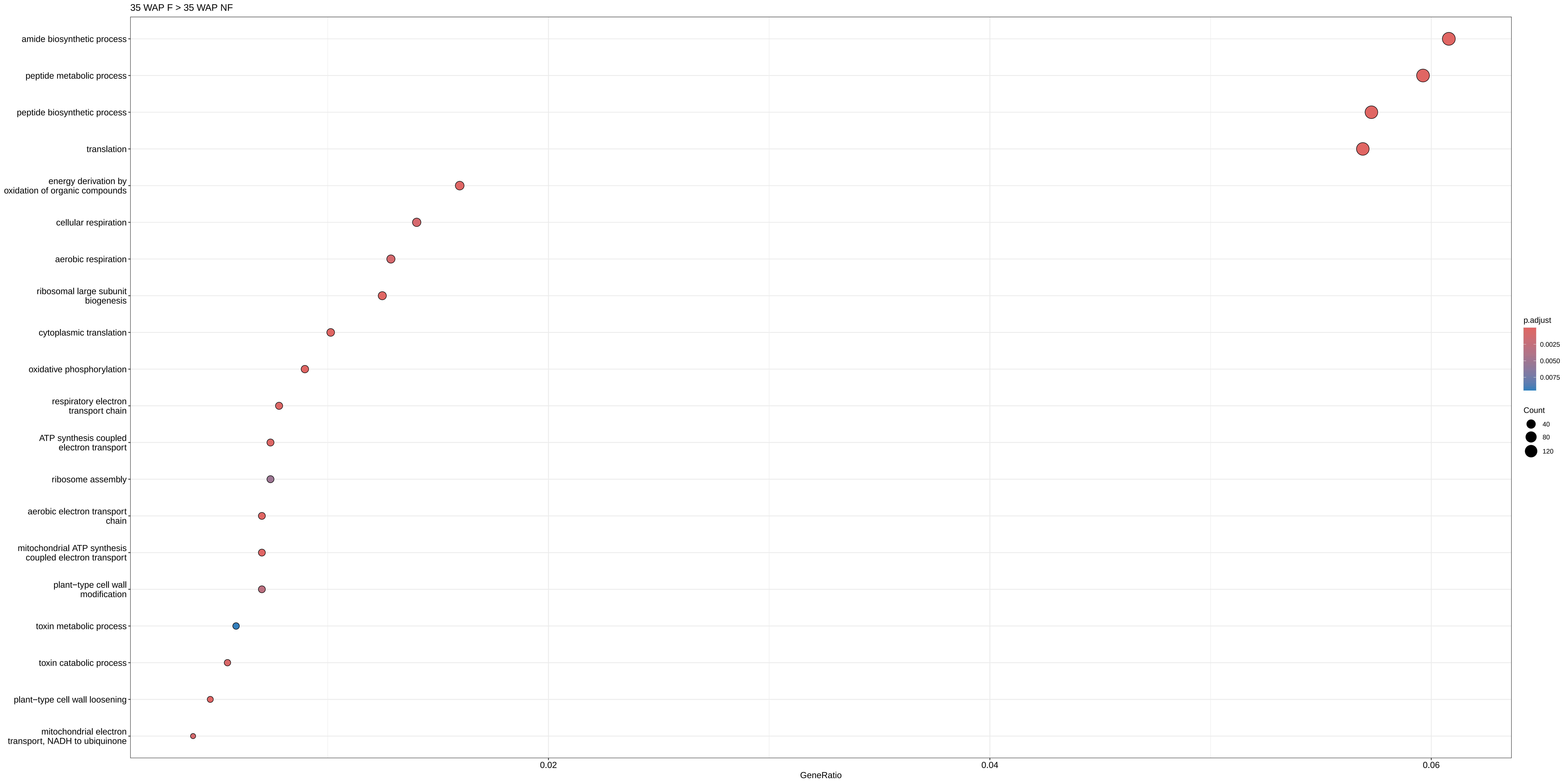
35 WAP F > 35 WAP NF
amide biosynthetic process
peptide metabolic process
peptide biosynthetic process
translation
energy derivation by oxidation of organic compounds
cellular respiration
aerobic respiration
ribosomal large subunit biogenesis
cytoplasmic translation
oxidative phosphorylation
respiratory electron transport chain
ATP synthesis coupled electron transport
ribosome assembly
aerobic electron transport chain
mitochondrial ATP synthesis coupled electron transport
plant−type cell wall modification
toxin metabolic process
toxin catabolic process
plant−type cell wall loosening
mitochondrial electron transport, NADH to ubiquinone
0.02
0.04
0.06
GeneRatio
p.adjust
0.0025
0.0050
0.0075
Count
40
80
120

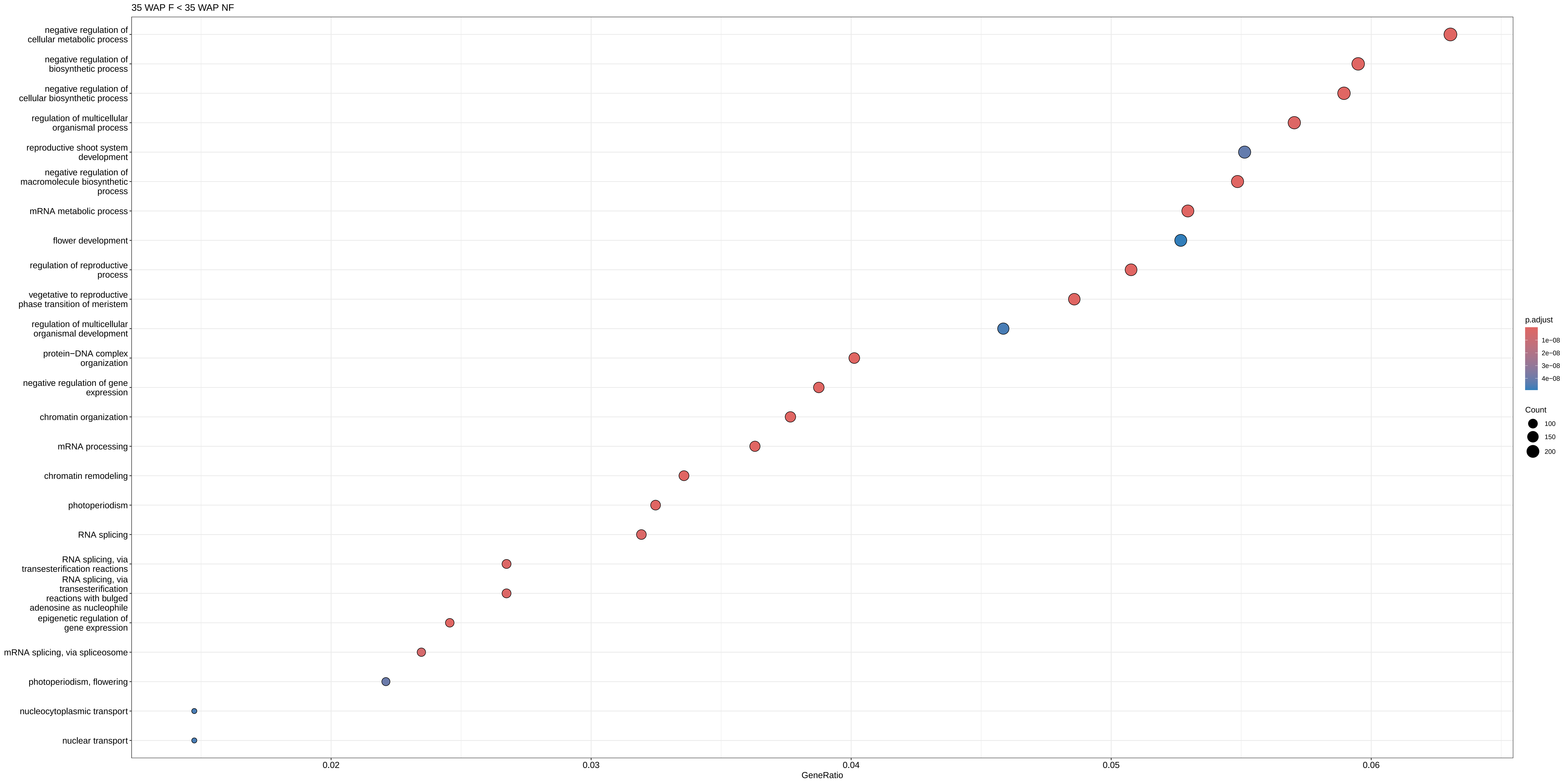

35 WAP F < 35 WAP NF
negative regulation of cellular metabolic process
negative regulation of biosynthetic process
negative regulation of cellular biosynthetic process
regulation of multicellular organismal process
reproductive shoot system development
negative regulation of macromolecule biosynthetic process
mRNA metabolic process
flower development
regulation of reproductive process
vegetative to reproductive phase transition of meristem
regulation of multicellular organismal development
protein−DNA complex organization
negative regulation of gene expression
chromatin organization
mRNA processing
chromatin remodeling
photoperiodism
RNA splicing
RNA splicing, via transesterification reactions
RNA splicing, via transesterification reactions with bulged adenosine as nucleophile
epigenetic regulation of gene expression
mRNA splicing, via spliceosome
photoperiodism, flowering
nucleocytoplasmic transport
nuclear transport
0.02
0.03
0.04
0.05
0.06
GeneRatio
p.adjust
1e−08
2e−08
3e−08
4e−08
Count
100
150
200

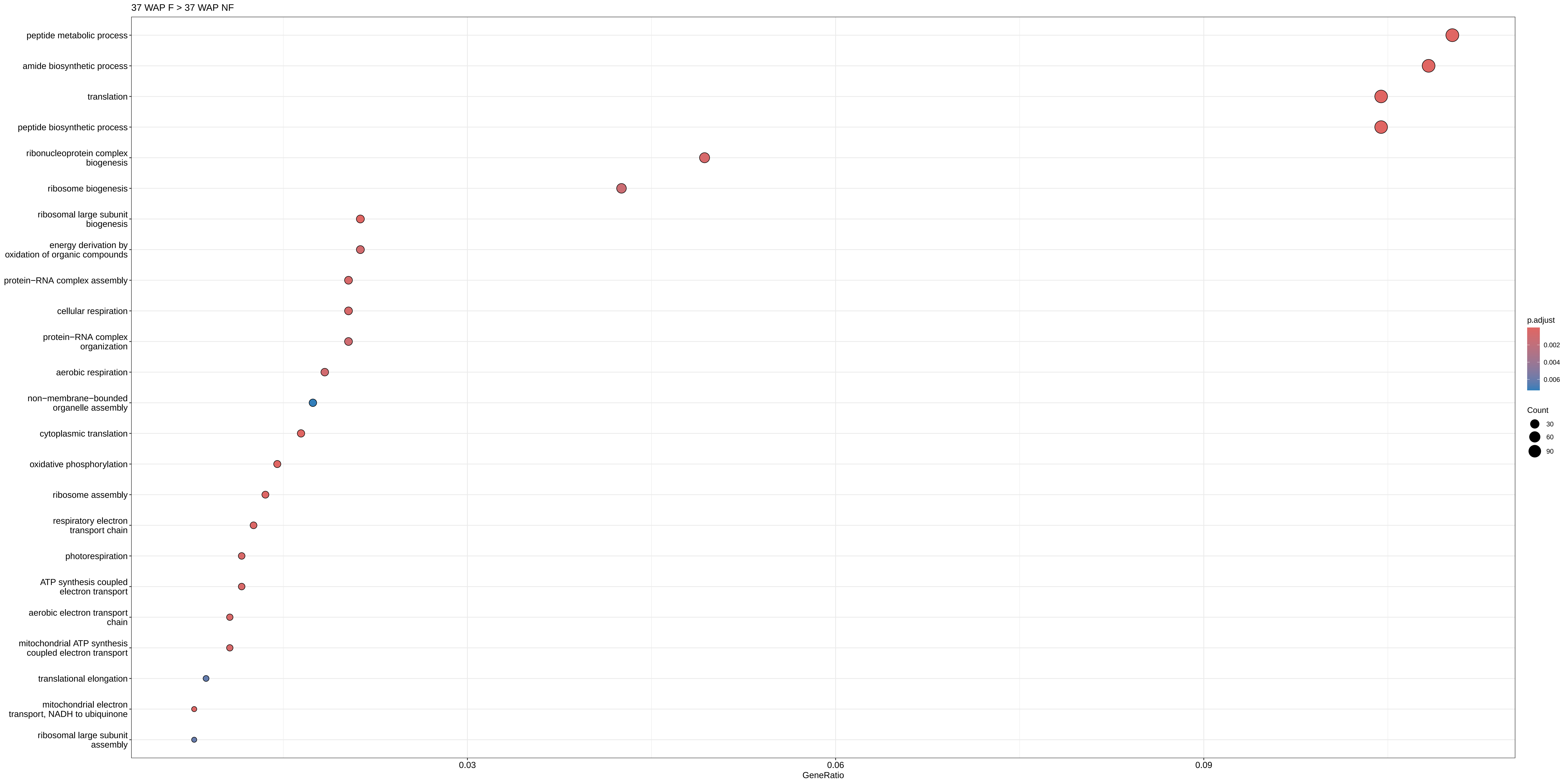

37 WAP F > 37 WAP NF
peptide metabolic process
amide biosynthetic process
translation
peptide biosynthetic process
ribonucleoprotein complex biogenesis
ribosome biogenesis
ribosomal large subunit biogenesis
energy derivation by oxidation of organic compounds
protein−RNA complex assembly
cellular respiration
protein−RNA complex organization
aerobic respiration
non−membrane−bounded organelle assembly
cytoplasmic translation
oxidative phosphorylation
ribosome assembly
respiratory electron transport chain
photorespiration
ATP synthesis coupled electron transport
aerobic electron transport chain
mitochondrial ATP synthesis coupled electron transport
translational elongation
mitochondrial electron transport, NADH to ubiquinone
ribosomal large subunit assembly
0.03
0.06
0.09
GeneRatio
p.adjust
0.002
0.004
0.006
Count
30
60
90

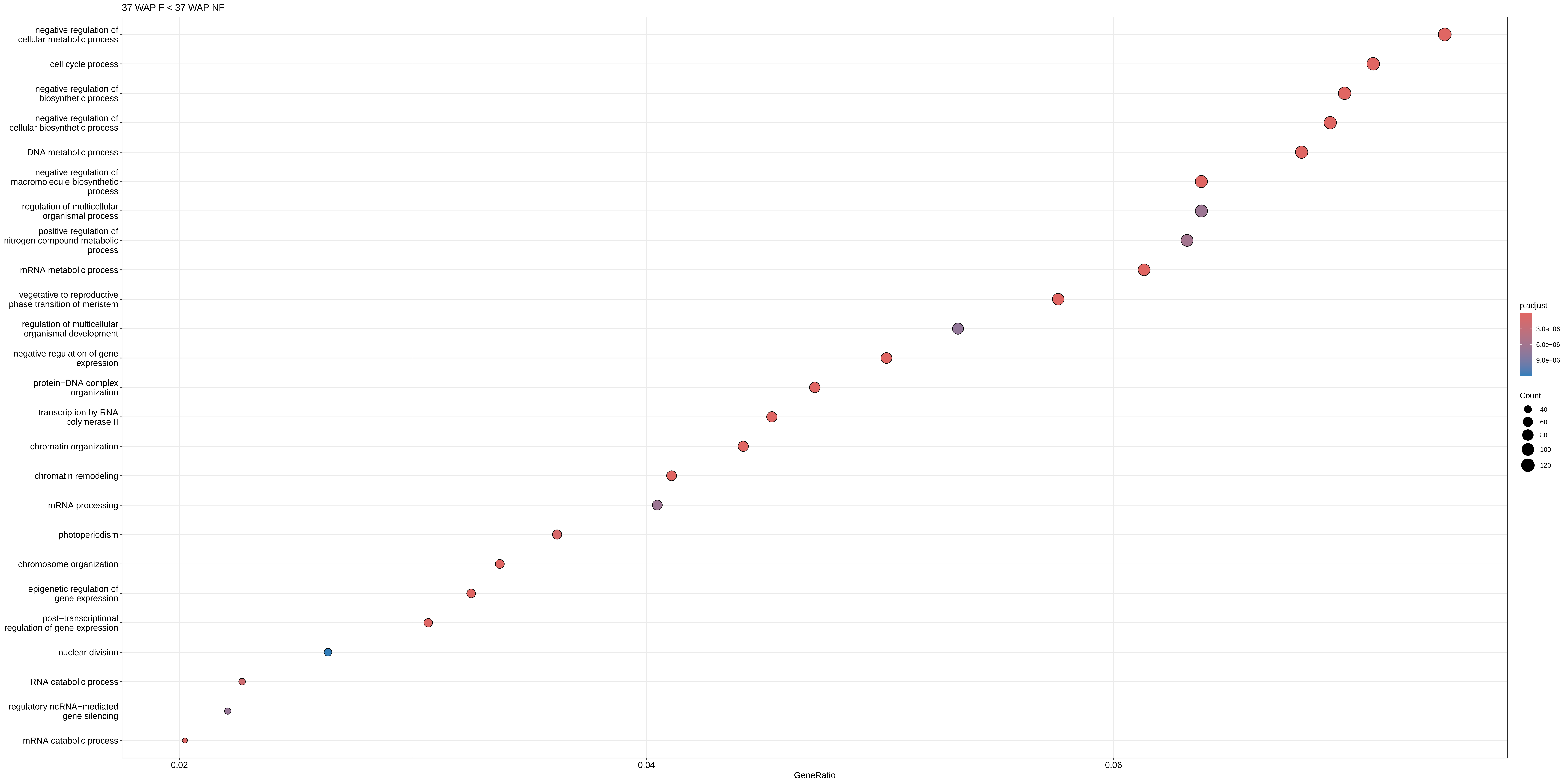
37 WAP F < 37 WAP NF
negative regulation of cellular metabolic process
cell cycle process
negative regulation of biosynthetic process
negative regulation of cellular biosynthetic process
DNA metabolic process
negative regulation of macromolecule biosynthetic process
regulation of multicellular organismal process
positive regulation of nitrogen compound metabolic process
mRNA metabolic process
vegetative to reproductive phase transition of meristem
regulation of multicellular organismal development
negative regulation of gene expression
protein−DNA complex organization
transcription by RNA polymerase II
chromatin organization
chromatin remodeling
mRNA processing
photoperiodism
chromosome organization
epigenetic regulation of gene expression
post−transcriptional regulation of gene expression
nuclear division
RNA catabolic process
regulatory ncRNA−mediated gene silencing
mRNA catabolic process
0.02
0.04
0.06
GeneRatio
p.adjust
3.0e−06
6.0e−06
9.0e−06
Count
40
60
80
100
120
